# Telmisartan *versus* metformin in downregulating myostatin gene expression and enhancing insulin sensitivity in the skeletal muscles of type 2 diabetic rat model

**DOI:** 10.3389/fphar.2023.1228525

**Published:** 2023-07-28

**Authors:** Ahmed Abd-Eltawab Tammam, Waleed Y. Rizg, Amy Fakhry Boushra, Maha Alhelf, Mohammed Alissa, Ghada F. Soliman, Ghada Nady Ouais, Khaled M. Hosny, Hala M. Alkhalidi, Ahmed Magdy Elebiary

**Affiliations:** ^1^ Medical Physiology Department, Faculty of Medicine, Beni-Suef University, Beni-Suef, Egypt; ^2^ Center of Innovation in Personalized Medicine (CIPM), 3D Bioprinting Unit, King Abdulaziz University, Jeddah, Saudi Arabia; ^3^ Department of Pharmaceutics, Faculty of Pharmacy, King Abdulaziz University, Jeddah, Saudi Arabia; ^4^ Medical Physiology Department, Faculty of Medicine, Fayoum University, Fayoum, Egypt; ^5^ Biotechnology School, Nile University, Giza, Egypt; ^6^ Medical Biochemistry and Molecular Biology Department, Faculty of Medicine, Cairo University, Cairo, Egypt; ^7^ Department of Medical Laboratory Sciences, College of Applied Medical Sciences, Prince Sattam bin Abdulaziz University, Al-Kharj, Saudi Arabia; ^**8**^ Medical Pharmacology Department, Faculty of Medicine, Cairo University, Cairo, Egypt; ^9^ Medical Pharmacology Department, Armed Forces College of Medicine, Cairo, Egypt; ^10^ Anatomy and Embryology Department, Faculty of Medicine, Cairo University, Cairo, Egypt; ^11^ Anatomy and Embryology Department, Faculty of Medicine, New Giza University, Giza, Egypt; ^12^ Department of Clinical Pharmacy, Faculty of Pharmacy, King Abdulaziz University, Jeddah, Saudi Arabia

**Keywords:** antidiabetic drugs, insulin resistance, metformin, myostatin, telmisartan, type 2 DM

## Abstract

**Objective:** Telmisartan is an angiotensin receptor blocker (ARB) that specifically blocks angiotensin II type-1 receptors (AT1R). Telmisartan has been proven to have antidiabetic effects via a variety of mechanisms, and it can be utilized in some diabetic patients due to its dual benefit for hypertensive patients with type 2 DM (T2DM) and when the other oral antidiabetic medications are intolerable or contraindicated. However, its precise underlying hypoglycemic mechanism is still obscure.

**Aim of work:** We sought to establish a link between telmisartan administration and myostatin expression in skeletal muscles of T2DM rat model as a potential hypoglycemic mechanism of telmisartan.

**Materials and Methods:** 32 male albino rats were included in the study; 8 rats served as controls (group I). T2DM was inducted in the other 24 rats, which were then randomly subdivided into 3 groups (8 in each): (group II) the Diabetic group and (groups III and IV) which were treated with either telmisartan (8 mg/kg/day) or metformin (250 mg/kg/day) respectively via oral gavage for a 4-week period.

**Results:** Telmisartan administration resulted in a significant improvement in OGTT, HOMA-IR, glucose uptake, and muscle mass/body ratios in Telmisartan group as compared to Diabetic group (*p* < 0.05). Additionally, telmisartan induced a significant boost in adiponectin and IL-10 serum levels with a substantial drop in TNF-α and IL-6 levels in Telmisartan group compared to diabetic rats (*p* < 0.05). Moreover, telmisartan significantly boosted SOD and GSH, and decreased MDA levels in the skeletal muscles of telmisartan group. Furthermore, a significant downregulation of myostatin and upregulation of insulin receptor, IRS-1, and IRS-3 genes in the skeletal muscles of Telmisartan group were also detected. Histologically, telmisartan attenuated the morphological damage in the skeletal muscle fibers compared to diabetic rats, as evidenced by a considerable decrease in the collagen deposition area percentage and a reduction in NF-kB expression in the muscle tissues of group III.

**Conclusion:** Telmisartan administration dramatically reduced myostatin and NF-kB expressions in skeletal muscles, which improved insulin resistance and glucose uptake in these muscles, highlighting a novel antidiabetic mechanism of telmisartan in treating T2DM.

## 1 Introduction

Myostatin is one of the superfamily members of transforming growth factor-β (TGF-β), which has a negative regulatory impact on the skeletal muscle’s growth and protein accretion. Therefore, cattle and mice with genetic myostatin deficiency show a massive increase in muscle bulk (Lee, 2007)**.** Myostatin is mainly produced by skeletal muscles and to a lesser extent by adipose tissues. Then it is released systemically into circulation and it binds to cell membrane receptors, causing muscle wasting, which is characterized by decreased protein content of the myofibrils in addition to a decrease in mass and performance of skeletal muscles as in a variety of catabolic conditions like sarcopenia, chronic diseases, and cancers (Glass and Roubenoff, 2010)**.**

In addition to the impact of myostatin on muscle bulk, this protein is thought to play a crucial role in the communication between the skeletal muscles and adipose tissues, and it appears to be extensively implicated in glucose uptake and in the response of these tissues to insulin. Inhibition of myostatin expression improves insulin sensitivity and enhances adiponectin release from adipocytes (Argilés et al., 2012)**.** In transgenic rats with the suppressed function of myostatin, insulin signaling was significantly activated and high-fat diet failed to cause glucose intolerance or insulin resistance as compared to wild-type rats (Zhao et al., 2005). Decreased insulin resistance in myostatin deficient rats was attributed to a significant activation of the AMPK signaling pathway and increased adiponectin, an insulin sensitizing adipocytokine, secretion which enhances the oxidation of fatty acids in skeletal muscles. This proves the valuable communication between adipose tissues and skeletal muscles, which could prevent the incidence of insulin resistance and obesity (Zhang et al., 2011), (Akpan et al., 2009).

Myostatin also appears to have an impact on the other pathological conditions that depend on the production of insulin as it has been linked also to T1DM. In the first stages of type 1 diabetic rats, the levels of myostatin expression in the skeletal muscles rise. Moreover, the patterns of myostatin expression were neatly correlated with the patterns of body weight loss and the expression of atrogin-1. Additionally, insulin injection has been demonstrated to suppress the expression of myostatin in type 1 diabetic rats (Chen et al., 2009).

Telmisartan is an angiotensin receptor blocker (ARB) that selectively blocks angiotensin II type-1 receptors (AT1R) without affecting the other cardiovascular regulatory receptors (Ayza et al., 2020). Blockage of angiotensin receptors may have a crucial role not merely in the management of atherosclerotic cardiovascular diseases but also in the correction of dyslipidemia, protection against type 2 DM (T2DM), and treatment of metabolic syndrome (Kurtz, 2006). Telmisartan is considered the first-line medication for most mild to moderate hypertensive cases due to its outstanding safety profile, and it can protect against hypertension-induced end-organ failure (Borém et al., 2018)**.** Telmisartan is a partial agonist for the peroxisome proliferator activated receptor-γ (PPAR-γ), stimulating about 25% of the receptor, with a wider safety margin and lesser adverse effects as compared to the full agonists of PPAR-γ such as thiazolidinediones (TZD) (Amano et al., 2012). PPARs are classified into three subclasses: PPAR-α, PPAR-β/δ, and PPAR-γ which is the most investigated receptor and more implicated in maintaining the lipid and glucose homeostasis and energy balance (Rakhshandehroo et al., 2010; Tyagi et al., 2011)**.**

Several studies postulated the antidiabetic effects of telmisartan through diverse mechanisms such as regulation of PPAR- γ phosphorylation with the subsequent genomic effects and upregulation of glucose transporter-4 (GLUT-4) gene expression that potentiated insulin response and glucose uptake in adipocytes (Fang et al., 2018), (Fujimoto et al., 2004)**.** Moreover, telmisartan was demonstrated to increase the secretion of adiponectin which binds to its receptors inducing a potent insulin-sensitizing effect (Watanabe et al., 2010)**.** Additionally, telmisartan has been demonstrated to improve the endothelial functions by stimulating eNOS, thus inducing the production of nitric oxide (NO), increasing the bioavailability of NO, and suppressing oxidative stress, thereby alleviating vascular inflammation which provide renal protection for diabetics (Zhang et al., 2012)**,** and safeguard the hypertensive patients with type 2 DM (T2DM) from atherosclerosis and cardiac hypertrophy, fibrosis and remodeling (Vanitha and Vijayal, 2013), (Maejima et al., 2011) **.** However, the exact mechanism by which telmisartan can lower blood glucose levels is still indefinite.

Metformin is a widely prescribed, affordable, and relatively well-tolerated biguanide. Nowadays, it is considered the first-line oral medication for the management of T2DM, which can be administered singly or in combination with other antidiabetic drugs (Sanchez-Rangel and Inzucchi, 2017)**.** Since metformin has a plant origin and was not initially synthesized to target specific pathways or receptors, its precise mechanism in lowering blood glucose levels is still obscure, however it has been demonstrated to exert its euglycemic effect via several ways, including decreasing glucose absorption from small intestine, inhibiting gluconeogenesis via suppressing the glycerophosphate dehydrogenase enzymatic activity in hepatic mitochondria, inducing glucose uptake by skeletal muscles, inhibiting lipolysis and promoting free fatty acids oxidation through reducing the activity of mitochondrial complex I and activation of adenosine monophosphate activated protein kinase (AMPK) of adipocytes which stimulates insulin signaling indirectly via depressed lipotoxicity (Baker et al., 2021)**.**

Despite the broad safety profile of metformin, it may have considerable adverse effects that may enforce a significant percentage of patients to discontinue this drug like, GIT troubles such as loss of appetite, nausea, abdominal discomfort, vomiting, and diarrhea, which are considered the commonest metformin side effects and about 5% of patients discontinue using it due to severe GIT manifestations (Shurrab and Arafa, 2020)**.** Moreover, metformin can induce lactic acidosis especially in patients with other comorbidities that cause a disturbance in the oxidative pathway of lactate like congestive heart failure, septicemia, and hepatic or renal insufficiency (DeFronzo et al., 2016)**.** Additionally, metformin has been reported to induce less frequent side effects such as acute pancreatitis (Alsubaie and Almalki, 2013)**,** hepatotoxicity (Kutoh, 2005)**,** vitamin B12 malabsorption and the subsequent development of pernicious anemia (Sanchez-Rangel and Inzucchi, 2017), (Katsaros et al., 2003)**,** and eventually producing disturbances in coagulation/fibrinolytic systems (Akinci et al., 2008)**.**

Suppression of myostatin expression in skeletal muscles has been identified as a potential factor in improving insulin sensitivity and protecting against the development of T2DM, according to previous literature (Allen et al., 2011; Dong et al., 2016). To be mentioned, myostatin treatment significantly activated the extracellular signal-regulated kinase 1/2 (ERK1/2) pathway and thus key adipogenic transcription factor peroxisome proliferator-activated receptor-gamma (PPAR-γ), was inhibited in a study done by (Pan et al., 2021). On the other hand, Telmisartan has been shown to exert euglycemic effects via diverse mechanisms including regulation of PPAR-γ phosphorylation where telmisartan is a structurally unique ARB that acts as a partial PPAR-γ agonist (Fujimoto et al., 2004; Watanabe et al., 2010; Fang et al., 2018). At the same time it could be a promising choice due to its fewer side effects compared to metformin and its dual benefit for hypertensive with T2DM patients. However, the precise underlying hypoglycemic mechanism of telmisartan is still obscure.

### 1.1 Aim of work

To the best of our knowledge, this is the first study to correlate between telmisartan administration and myostatin expression in the skeletal muscles of type 2 diabetics. Therefore, the present study aimed to elucidate whether telmisartan can improve insulin sensitivity through downregulating myostatin expression in skeletal muscles, and to compare its effect to metformin as a widely used antidiabetic drug. This novel point of study may propose a potential antidiabetic mechanism of telmisartan in type 2 DM.

## 2 Materials and methods

### 2.1 Chemicals

Streptozotocin (STZ) was purchased from Sigma Chemical Company (St. Louis, United States). Telmisartan 40 mg tablets were obtained from Boehringer Ingelheim Pharma GmbH & Co. KG, for: Boehringer Ingelheim International GmbH, Germany, and imported by the Egyptian Company for Pharmaceutical Trade. Metformin 500 mg tablets were purchased from Minapharm Pharmaceuticals, Egypt.

### 2.2 Experimental animals and ethical consideration

32 male Albino rats, 13–14 weeks old and weighing 190–210 g were obtained from the Experimental Animal Research Center of Faculty of Science, Fayoum University and housed in the Animal House of Faculty of Medicine, Fayoum University. The animals underwent acclimatization for 10 days before to the experiment to rule out any concomitant infection. Rats were kept in wire mesh crates under conventional housing circumstances of 23 ± 2°C, 44% ± 4% humidity, and normal light/dark cycle. Chow and water were offered to the rats *ad libitum*. The protocol of this study was endorsed by The Scientific Research Ethical Committee, Faculty of Medicine, Fayoum University, Egypt (Approval No: R199, session (88), August 2021).

### 2.3 Study design

After the 10 days of acclimatization, the rats were randomly distributed into four groups: one control group and three diabetic groups (8 rats per each).

• Control group (group I): the rats were fed a standard normal diet and received an i.p single dose of citrate buffer.
• Diabetic groups (groups II, III and IV): The rats were fed with high fat diet (HFD): (60% fat, 20% protein, and 20% carbohydrates) for 14 days, then one low dose of streptozotocin (STZ) was intraperitoneally injected (45 mg/kg, in 0.01 M citrate buffer, pH 4.3) to induce T2DM (Si et al., 2012), (Khan et al., 2017). After STZ injection, free access to water and food was maintained for rats according to their relevant type of diet until the end of experiment. One week after STZ administration blood samples were taken randomly from the diabetic groups to measure serum glucose to confirm the occurrence of T2DM. Rats with fasting glucose level over 13 mmol/L were considered diabetic (Yang et al., 2011).

After confirmation of T2DM, we started the treatment in the third and fourth groups as follows.

• Diabetic group receiving telmisartan (group III): 8 mg/kg/day of telmisartan suspended in carboxymethyl cellulose (CMC) 0.5% was given via oral gavage for 4 weeks (Cheng et al., 2018).
• Diabetic group receiving metformin (group IV): 250 mg/kg/day of metformin was administered orally for 4 weeks (Bahriz et al., 2020).

At the end of the study and after an overnight fasting, the oral glucose tolerance was done (as shown below). Rats were weighed then anesthetized with an i.p injection of 90 mg/kg ketamine. 3 mL of blood samples were withdrawn from each rat’s tail vein and centrifuged. The collected sera were kept at −80°C for later serological biochemical assays of insulin, adiponectin, TNF α, IL-6 and IL-10. The rats were then executed via cervical dislocation for collection of tissue samples.

### 2.4 Tissue samples collection and storage

After scarification, the gastrocnemii, solei and plantaris muscles were dissected and weighed for assessment of muscle mass. The histological and immunohistochemical examinations were carried out on the right gastrocnemius muscle. The biochemical and genetic testing was done on the left gastrocnemius muscle which was inserted in liquid nitrogen then kept at −80°C for later homogenization and analysis of insulin-dependent glucose uptake in muscle, and the expressions of myostatin, insulin receptor, insulin receptor substarte-1 (IRS-1), and insulin receptor substrate-3 (IRS-3) genes. Colorimetric measurement of MDA, GSH and SOD was also done.

### 2.5 Oral glucose tolerance test

OGTT was performed at the end of the experiment, and following an overnight fasting (12 h), a 2-gm oral glucose load per kg body weight was given via oral gavage. For measuring blood glucose levels, blood was taken from tail vein of rats at times 0 (before the glucose loading), 30, 60, 90, and 120 min after the glucose loading. The procedure was described by (Sornalakshmi et al., 2016). Glucose was then measured using colorimetric assay kit (Invitrogen™, *Catalog No. EIAGLUC*).

### 2.6 Assessment of serum insulin, adiponectin, TNF-α, IL-6, and IL-10

Using the Elabscience^®^ Rat Insulin ELISA Kit, *Catalog No. E-EL-R3034*, serum insulin was quantitatively evaluated according to the manufacturer’s instructions.

Rat ELISA kits (*CUSABIO, TX, United States*) for adiponectin (*Catalog No. CSB-E07271r*), TNF-α (*Catalog No. CSB-E11987r),* IL-6 (*Catalog No. CSB-E04640r*), and IL-10 (*Catalog No. CSB-E04595r*) were utilized for the quantitative determination of the mentioned parameters in the serum of the rats according to the instructions from the manufacturer.

### 2.7 Calculation of homeostasis model assessment of insulin resistance

HOMA-IR index was assessed by the following formula:

HOMA-IR={Fasting insulin (μIU/ml)×fasting glucose (mmol/L)}/ 22.5

Lower index means greater insulin sensitivity. Values > 4 were considered as having insulin resistence (Bowe et al., 2014).

### 2.8 Colorimetric assessment of tissue oxidative stress biomarkers

50 mg of the left gastrocnemius muscle was homogenized and centrifuged for 10 minutes at 1,200 rpm while being preserved in 0.1 mol phosphate buffer saline (PBS) solution. The supernatant was used to determine the tissue levels of Malondialdehyde (MDA), Reduced Glutathione (GSH) and Superoxide dismutase (SOD).

Malondialdehyde (MDA) concentration in muscle tissue homogenate was measured colorimetrically using a lipid peroxidation (MDA) assay kit (*Biodiagnostic, Giza, Egypt*), according to the manufacturer’s instructions.

Utilizing the reduced glutathione colorimetric kit (*Biodiagnostic, Giza, Egypt*), reduced glutathione (GSH) content was determined in line with the manufacturer’s guidelines.

Superoxide dismutase (SOD) activity in muscle tissue was measured by Superoxide Dismutase Activity Colorimetric Assay Kit (Abcam, *Catalog No. ab65354*) following the manufacturer’s instructions.

### 2.9 Assessment of muscle glucose uptake

Following isolation, the left gastrocnemius muscle was immersed into Krebs Henseleit solution (KHS) and ventilated with carbogen (95% O_2_ and 5% CO_2_) as outlined by (Ueyama et al., 2000). The buffer had a total volume of 100 cm^3^, to which 100 mg glucose was added, along with soluble porcine insulin with a concentration of 250 µU/1 cm^3^ buffer. The pH of this incubation medium was kept at 7.4 using a pH meter.

Next, the muscle was placed in a glass flask containing 3 cm^3^ of the incubation liquid. Another flask containing only 3 cm^3^ of the same incubation liquid without adding muscle tissues was utilized as a control sample. Then, the two flasks were transferred into a metabolic shaker and shaken at a rate of 100 cycle/min for 1 h at 37^°^C under a tent of carbogen gas; the constant shaking altered the layers of incubation liquid, which are in contact with the muscle and gas phase. After incubation, muscles were quickly dried on filter paper and weighed. The glucose level was measured in 1 cm^3^ of each sample and in 1 cm^3^ of the control liquid. Glucose uptake by each muscle was assessed in mg/gram of muscle/1 h of incubation.

$$Glucose\;uptake=\frac{\left.Glucose\;concentration\;in\;Control\;sample-Sample\;glucose\;concentration\;after\;1\;hour\right.X\;3\;\left(Medium\;volume\right)}{\left(100\;X\;Muscle\;weight\;in\;gm\right)}$$

### 2.10 Assessment of muscle mass

After scarification, skeletal muscles were carefully resected from the hind limb by extending the leg by hanging it with the use of a burette clamp. The skin was removed gently to expose the underlying muscle tissues. Multiple muscles were removed; soleus (SOL), gastrocnemius (GSN), and plantaris muscles. They were blotted dry with filter paper and weighed on an analytical scale and the body weights of the rats were also measured as mentioned in 2.3 section (Bonetto et al., 2015), (Horii et al., 2016).

### 2.11 Quantitative real time PCR analysis

50 mg of the left gastrocnemius muscle was homogenized and centrifuged for 15 minutes at 5,000 rpm while being kept in 300 micro liter Lysis buffer. The supernatant was used for later RNA extraction.

Total RNA from isolated gastrocnemius muscle tissue homogenate was extracted according to the manufacturer’s instruction using RNeasy Mini Kits (Qiagen, Germany). RNA samples were analyzed for purity and quantification using the NanoDrop^®^ (ND)-1,000 spectrophotometer (NanoDrop Technologies, Inc. Wilmington, United States). Through a high-capacity cDNA reverse transcription kit (Fermentas, United States), cDNA was synthesized from extracted RNA (0.5–2 g). The fold change of insulin receptor, insulin receptor substarte-1 (IRS-1), insulin receptor substrate-3 (IRS-3) and myostatin mRNAs were quantified and amplified using Real-time qPCR with an Applied Biosystem and software version 3.1 (StepOneTM, United States of America). At the annealing temperature, the qPCR assay including the primer sets was optimized. β-Actin was utilized as the housekeeping gene. The gene-specific primer sequences were shown in Table 1.

**TABLE 1:** The primer sets used in the study.

	Forward primer	Reverse primer	GenBank accession number
**Insulin Receptor**	5′-GTG​CTG​CTC​ATG​TCC​TTA​GA-3′	5′-AAT​GGT​CTG​TGC​TCT​TCG​TG-3′	M29014
**IRS-1**	5′-GCC​AAT​CTT​CAT​CCA​GTT​GC-3′	5′- CAT​CGT​GAA​GAA​GGC​ATA​GG-3′	X58375
**IRS-3**	5′-GAG​ACC​GTC​CTG​GCT​GCC​AT-3′	5′-ATC​CAC​ATG​TAC​TCA​GCA​GC-3′	U93880
**Myostatin**	5′-ACG​CTA​CCA​CGG​AAA​CAA​TC-3′	5′-CCG​TCT​TTC​ATG​GGT​TTG​AT-3′	NM_019151
**β-Actin**	5′-GGT​ATG​GAA​TCC​TGT​GGC​ATC​CAT​GAA​A-3′	5′- GTG​TAA​AAC​GCA​GCT​CAG​TAA​CAG​TCC​G-3′	V01217J00691

Following the PCR cycles, melting curve analyses were performed to ensure the accurate generation of the anticipated PCR product. The data were assessed by applying the threshold cycle (2^−ΔΔCT^) method of comparative PCR.

### 2.12 Histopathological examination

The gastrocnemii muscles from the rats of each group were immersed in 10% neutral buffered formalin for 48 h, embedded in paraffin, and cut into 4–7 mm sections. Tissue sections were then exposed to dimethylbenzene for dewaxing and an ethanol gradient for rehydration prior to the stain with hematoxylin and eosin (H&E) for the general histological features and Masson’s trichrome stain for collagen fibers examination (Bancroft and Gamble, 2008)**.** Slides were observed under a light microscope (BX53, Olympus, Japan).

### 2.13 Immunohistochemical staining

4 μm thick sections were prepared from the right gastrocnemii muscles of the different animal groups. Sections were deparafinized in xylene. Then, washed in descending grades of ethanol (100%, 95%, 70%) two changes 5 min each, rehydrated in phosphate buffer saline (PBS) by using a steamer in citrate buffer with pH 6.0 for 15 min and the activity of the endogenous peroxidase was blocked by H_2_O_2_ in methanol. Sections have been pre-treated with citrate buffer in a microwave. Sections have been incubated with rabbit monoclonal anti-nuclear factor-kB (NF-kB) at the room temperature. Sections were treated with biotinylated goat anti-polyvalent, streptavidin peroxidase, and then DAB with chromogen. Slides were coated with neutral balsam after being counterstained with hematoxylin dehydrated in xylene and alcohol. Under a light microscope, the slides were observed, and the degree of cell immune positive was evaluated.

N.B: Blue coloration: negative reaction, Brown coloration: positive reaction.

### 2.14 Histomorphometric study

ImageJ analysis software was used to carry out the quantitative study. Ten non-overlapping microscopic areas were randomly selected from each slide to be used for the quantitative analysis of the rat muscle specimen. They were examined within the standard measuring frame of a known area equal to 11694.91 µm^2^.After that, these parameters were measured: 1) Area percent of collagen fibers in Masson trichrome stained sections 2) Area percent of NF-kB immune-reactivity in NF-kB**—**immunostained sections.

### 2.15 Statistical analysis

The statistical software for the social sciences (SPSS) version 28 (IBM Corp., NY, United States) was used to code and enter the data. The mean and standard deviation were used to summarize the data. Analysis of variance (ANOVA) with multiple comparisons *post hoc* test was used to compare the groups (Chan, 2003). Statistical significance was considered when *p*-values < 0.05.

## 3 Results

### 3.1 Oral glucose tolerance test

**FIGURE 1:**
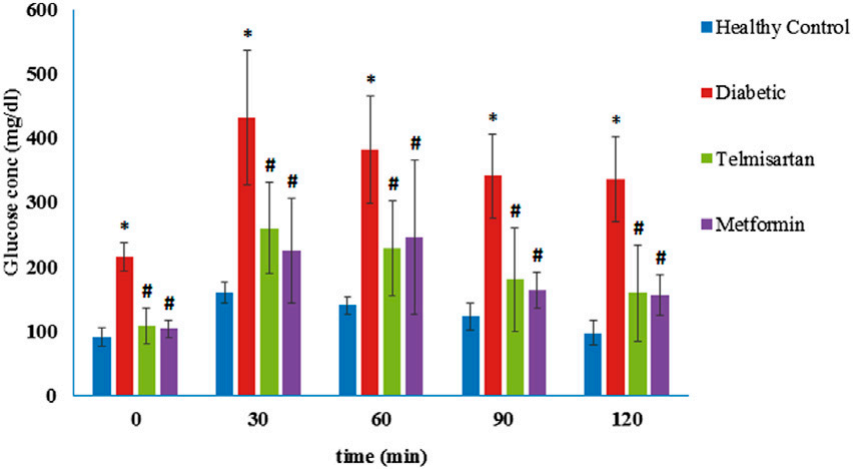
The oral glucose tolerance curve (OGTT) in the different groups of study and the effect of telmisartan and metformin treatment in groups III and IV respectively. Values are represented as mean ± SD (n = 8/group). *: *p < 0.05* compared to the Control group, #: *p < 0.05* compared to the Diabetic group, and $: *p < 0.05* compared to the Telmisartan group.

As shown in Figure 1, blood glucose levels at times 0 (before glucose loading), 30, 60, 90, and 120 min following the glucose loading for the diabetic rats were significantly elevated compared to the Controls (*p* < 0.05) (Figure 1). These levels were markedly improved and significantly decreased compared to the Diabetic group after treatment with either telmisartan or metformin in groups III and IV respectively. No statistically significant difference was noticed between the treated groups or between the treated groups and the Control group (*p* > 0.05).

### 3.2 Serum insulin, adiponectin, TNFα, IL-6 and IL-10

**FIGURE 2:**
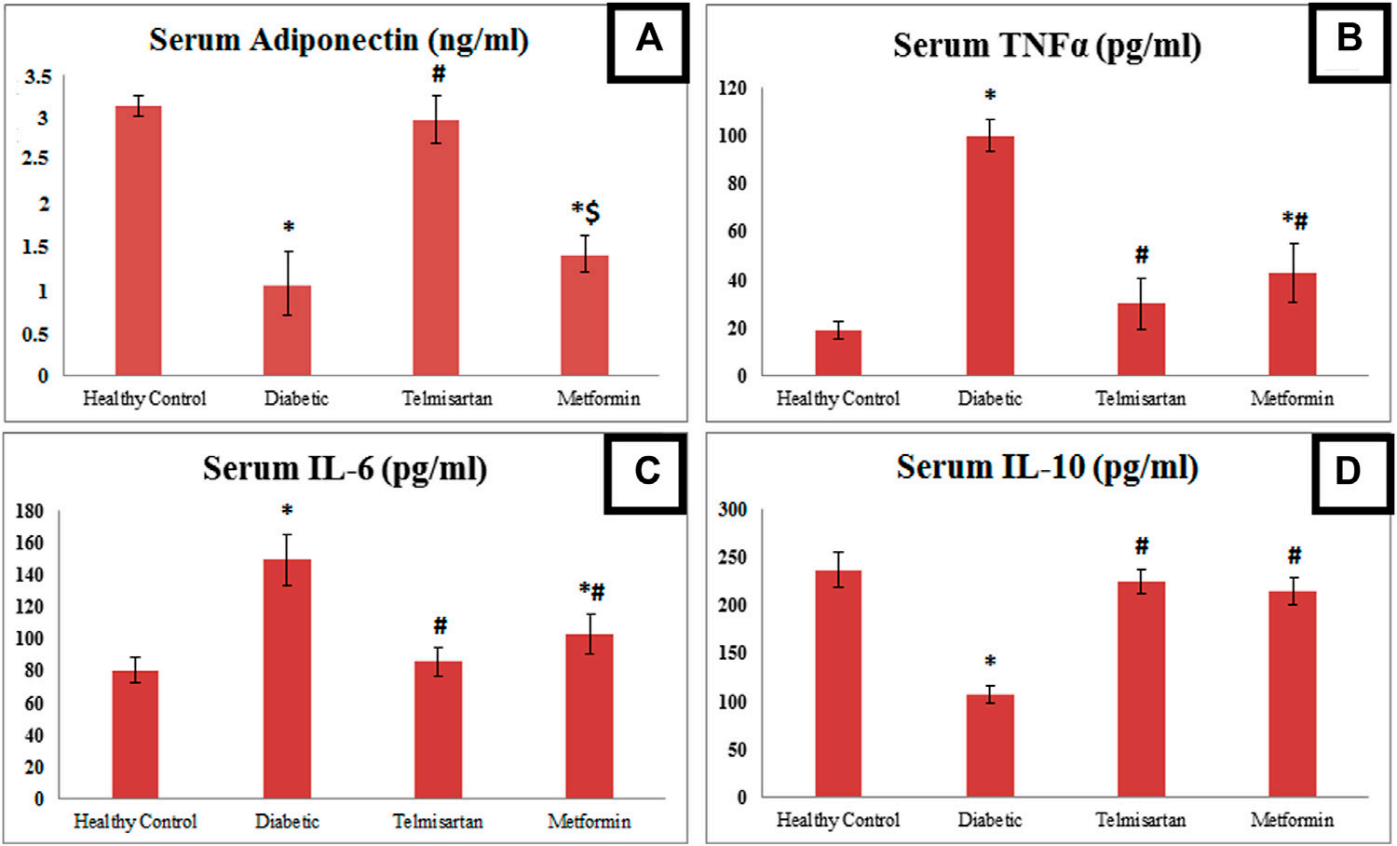
The effect of telmisartan and metformin treatment on serum adiponectin **(A)**, TNF-α **(B)**, IL-6 **(C)**, and IL-6 **(D)** in comparison to the Control and Diabetic groups. Values are represented as mean ± SD (n = 8/group). *: *p < 0.05* compared to the Control group, #: *p < 0.05* compared to the Diabetic group, and $: *p < 0.05* compared to the Telmisartan group.

The Diabetic group showed a significant elevation of insulin and reduction of adiponectin serum levels compared to the Control group (*p* < 0.05) (Table 2; Figure 2). After treatment with Telmisartan, serum insulin was reduced, and adiponectin was elevated significantly compared to the Diabetic rats, while no considerable difference observed between the Telmisartan and Control groups in both parameters. The Metformin group showed a significant reduction of insulin level compared to the Diabetic group, with a significant reduction of adiponectin still noticed when compared to both the Control and Telmisartan groups with no significant difference between this group and the Diabetic group (*p* > 0.05).

**TABLE 2:** Comparison between the serum insulin level and HOMA-IR in the different groups.

	Healthy control	Diabetic	Telmisartan	Metformin
**Serum Insulin**	0.86 ± 0.12	2.46 ± 0.44*	1.18 ± 0.32#	1.45 ± 0.3*#
**HOMA-IR**	1.33 ± 0.35	9.02 ± 1.12*	2.04 ± 0.61#	2.35 ± 0.4#

Values are represented as mean ± SD (n = 8/group). *: *p < 0.05* compared to the Control group, #: *p < 0.05* compared to the Diabetic group, and $: *p < 0.05* compared to the Telmisartan group.

Concerning inflammatory markers; TNFα, IL-6 were significantly elevated in the Diabetic group (group II) compared to the Control group (group I). These levels were significantly reduced compared to the Diabetic group in both Telmisartan and Metformin groups. However, results of both inflammatory markers in the Metformin group still showed a significant difference compared to the Control group (*p* < 0.05).

On the contrary for IL-10 as anti-inflammatory markers; it was markedly and significantly declined in the Diabetic rats compared to the Controls, with significant elevation after treatment with telmisartan or metformin compared to the Diabetic group with no substantial statistical difference between the treatment groups (groups III and IV) or between the treatment groups and the Control group (*p* > 0.05).

### 3.3 Homeostasis model assessment of insulin resistance (HOMA-IR)

The insulin resistance was determined by the HOMA index. Compared to the Control group (group I), the diabetic group showed a statistically significant elevation in HOMA-IR but was decreased significantly compared to the Diabetic group after treatment with telmisartan even more than Metformin (Table 2). No significant difference observed between the treated groups (groups III and IV) and the Control group (*p* > 0.05).

### 3.4 Reduction of diabetic-oxidative stress in the skeletal muscles of the treated groups

**FIGURE 3:**
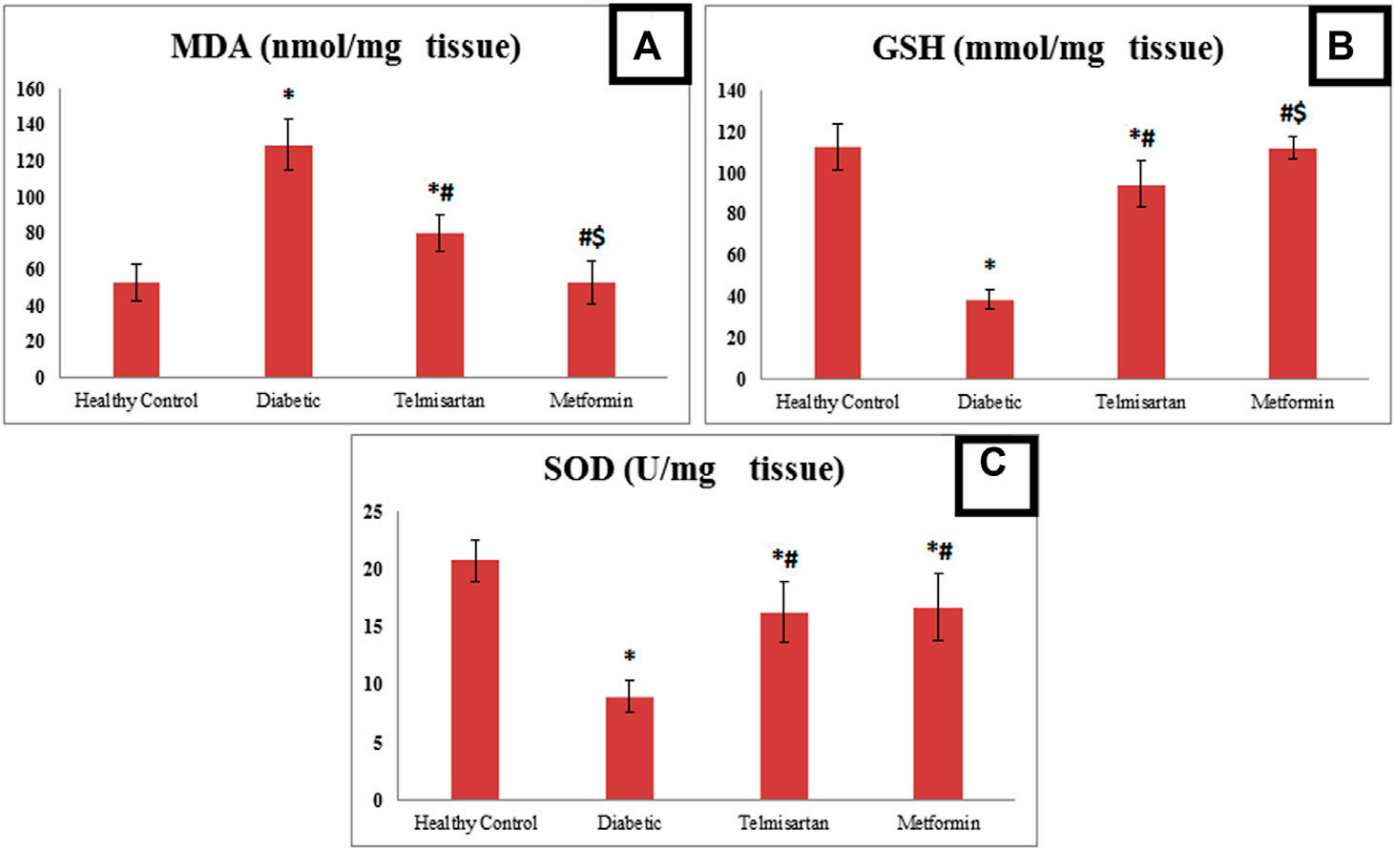
The effect of telmisartan and metformin treatment on the oxidative stress biomarkers; MDA **(A)**, GSH **(B)**, and SOD **(C)** in comparison to the Control and Diabetic groups. Values are represented as mean ± SD (n = 8/group). *: *p < 0.05* compared to the Control group, #: *p < 0.05* compared to the Diabetic group, and $: *p < 0.05* compared to the Telmisartan group.

Diabetes showed disequilibrium between oxidants/antioxidants parameters as indicated by a significant rise in MDA with a significant decline in GSH and SOD in the tissue of the left gastrocnemii muscles of the Diabetic group compared to the Control group (Figure 3).

However, treatment with either telmisartan or metformin successfully improved these levels. This was shown by the significant decline in MDA level in Telmisartan treated group compared to diabetic group, but a significant difference still noticed between the Telmisartan and Control groups. Metformin treated group showed a significant decrease compared to both the Diabetic and Telmisartan groups but not to the Control group (*p* < 0.05).

Treatment with either telmisartan or metformin increased GSH significantly compared to Diabetic group. A significant difference still noticed between Telmisartan group and both the Control and Metformin groups, while no significant difference existed between the Metformin and Control groups (*p* > 0.05).

Concerning SOD, Both Telmisartan or Metformin treatment increased significantly the SOD levels compared to diabetic group, but significant difference still existed between the treatment groups and the Control group (*p* < 0.05).

### 3.5 Gene expression of myostatin, insulin receptor, IRS-1, and IRS-3

**FIGURE 4:**
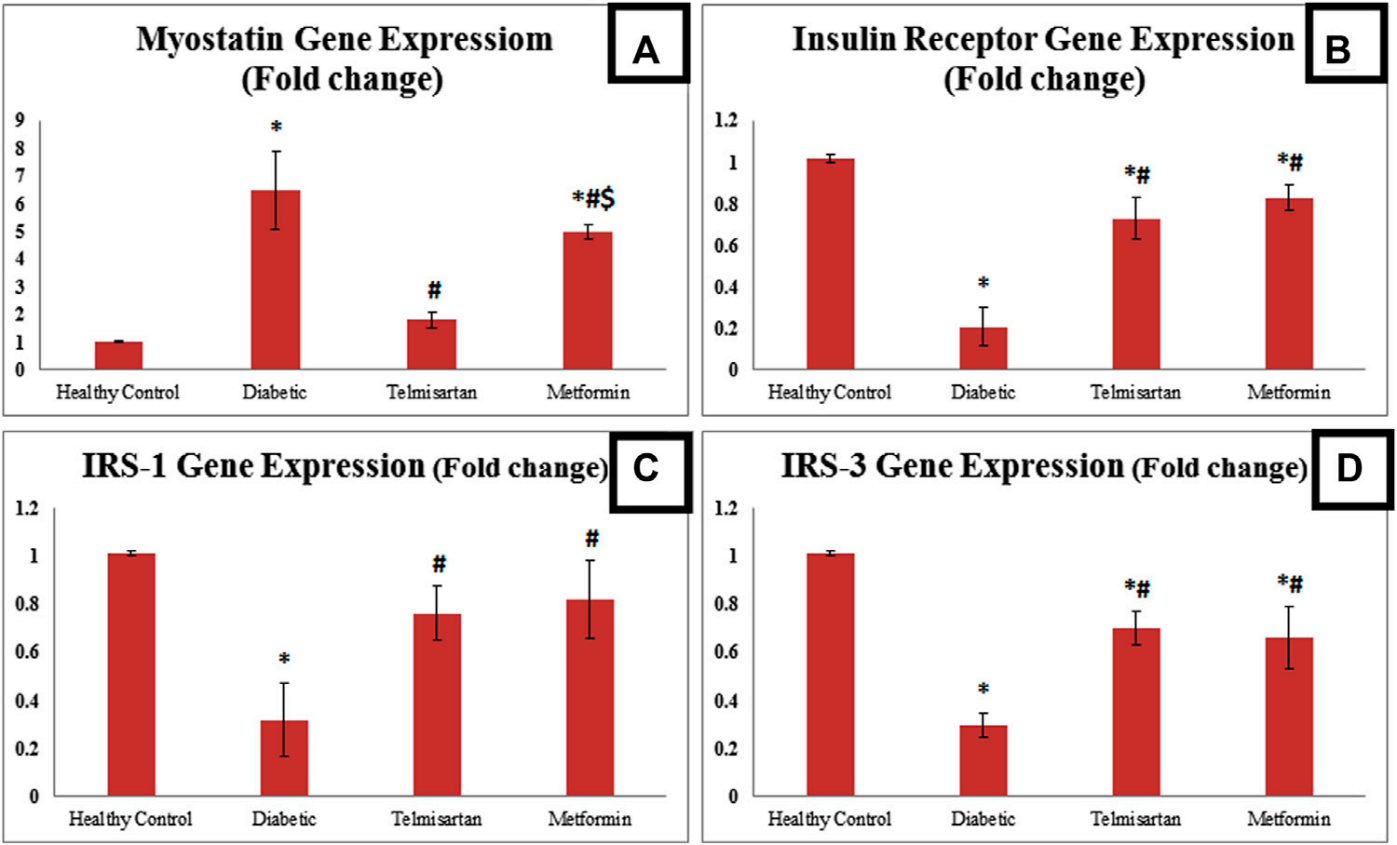
The effect of telmisartan and metformin treatment on the gene expression of myostatin **(A)**, insulin receptor **(B)**, IRS-1 **(C)**, and IRS-3 **(D)** in comparison to the Control and Diabetic groups. Values are represented as mean ± SD (n = 8/group). *: *p < 0.05* compared to the Control group, #: *p < 0.05* compared to the Diabetic group, and $: *p < 0.05* compared to the Telmisartan group.

Concerning the myostatin gene expression, it was significantly upregulated among the Diabetic rats compared to the Control group. Telmisartan and Metformin groups showed a significant downregulation in myostatin gene expression compared to the Diabetic group (*p* < 0.05) Figure 4. To be mentioned that, the myostatin gene expression in Telmisartan group showed more improvement as no significant difference existed when compared to the Control group, while Metformin group showed a statistically significant difference (*p* < 0.05) when compared to both the Control and Telmisartan groups.

Regarding insulin receptor gene expression, there was a statistically significant downregulation among the diabetic rats compared to the Control. Telmisartan and metformin treatment resulted in a significant upregulation in Insulin receptor gene expression compared to diabetic group (*p* < 0.05). However, a significant difference still noticed between the treatment groups and the control group (*p* < 0.05).

As for insulin receptor substarte-1 (IRS-1) gene expression, it was significantly downregulated among the diabetic group compared to the normal healthy rats. On the other hand, there was a significant statistical upregulation (*p* < 0.05) between the treated rats with either telmisartan or metformin compared to diabetic rats. No significant difference existed between the treatment groups and control rats.

Insulin receptor substrate-3 (IRS-3) gene expression was significantly downregulated among the diabetic rats compared to the Control rats. Both Telmisartan and Metformin groups showed a significantly upregulated IRS-3 gene expression compared to diabetic group, yet a significant difference still noticed between the treatment groups and the control group (*p* < 0.05).

### 3.6 Muscle glucose uptake

**FIGURE 5:**
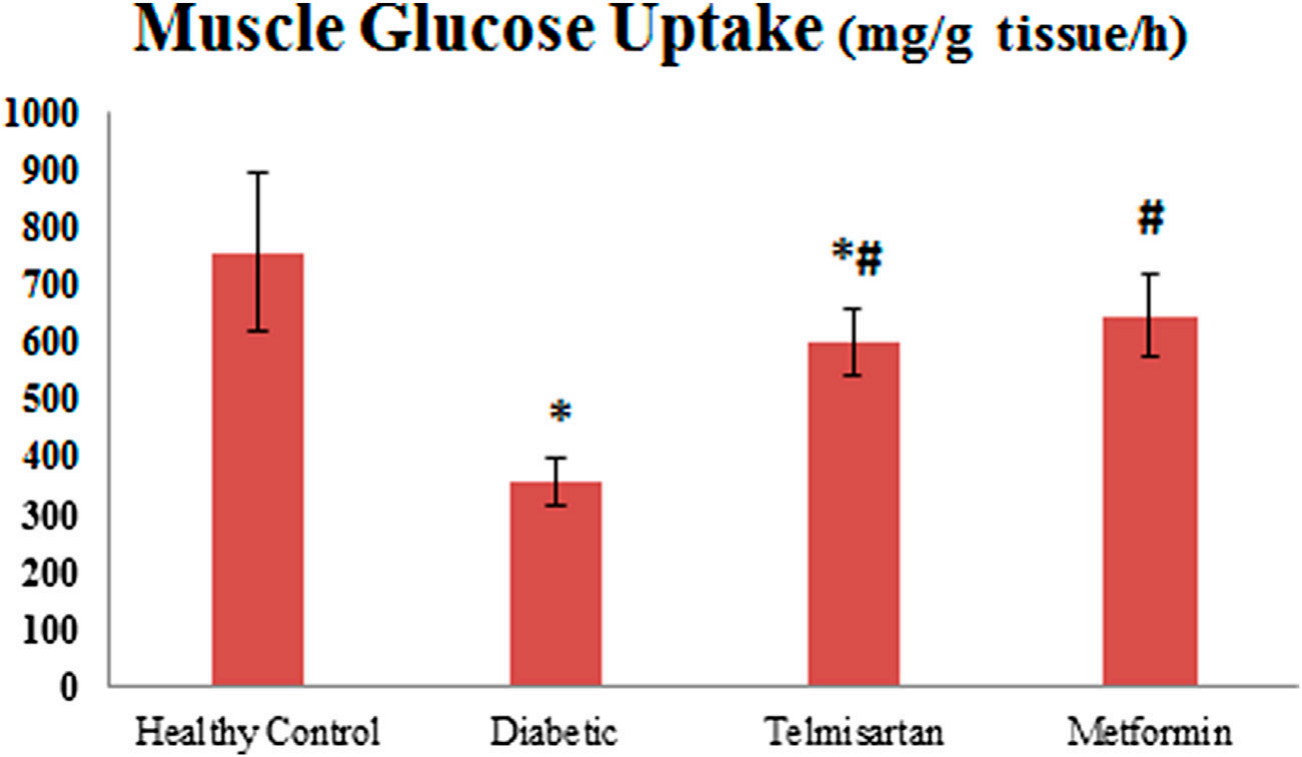
The effect of telmisartan and metformin treatment on the muscle glucose uptake in comparison to the Control and Diabetic groups. Values are represented as mean ± SD (n = 8/group). *: *p < 0.05* compared to the Control group, #: *p < 0.05* compared to the Diabetic group, and $: *p < 0.05* compared to the Telmisartan group.

The Diabetic group showed a significant decline of gastrocnemius muscle glucose uptake compared to the Control group (*p* < 0.05) Figure 5. Both the Telmisartan and metformin showed a significant improvement in muscle glucose uptake compared to the Diabetic group (*p* < 0.05) with no significant difference between the Metformin and Control groups (*p* > 0.05).

### 3.7 Muscle mass and body weight

Muscle mass ratio was obtained by dividing the muscle weight over the body weight. It was significantly lower in the diabetic group regarding the three assessed muscles (Soleus, Gastrocnemius and Plantaris) compared to the control group. Both the Telmisartan and Metformin groups showed a significant increase in the muscle mass ratio compared to the Diabetic group; however more improvement in the muscle mass was noticed in the Telmisartan group shown by a significant difference between the two treatment groups. Yet, there is still a significant difference between the treated groups (groups III and IV) and the normal rats (*p* < 0.05) (Table 3).

**TABLE 3:** Comparison between different muscle mass/body weight ratios in the different groups.

	Control	Diabetic	Telmisartan	Metformin
**Soleus muscle/Body Weight (mg/gm BW)**	0.37 ± 0.01	0.29 ± 0.01*	0.35 ± 0.01*#	0.33 ± 0.01*#$
**Gastrocnemius muscle/BW (mg/gm BW)**	4.51 ± 0.14	2.92 ± 0.07*	3.84 ± 0.07*#	3.20 ± 0.11*#$
**Plantaris muscle/BW (mg/gm BW)**	0.81 ± 0.02	0.60 ± 0.02*	0.74 ± 0.02*#	0.67 ± 0.02*#$

Values are represented as mean ± SD (n = 8/group). *: *p < 0.05* compared to the Control group, #: *p < 0.05* compared to the Diabetic group, and $: *p < 0.05* compared to the Telmisartan group.

### 3.8 Histological examination of stained sections

**FIGURE 6:**
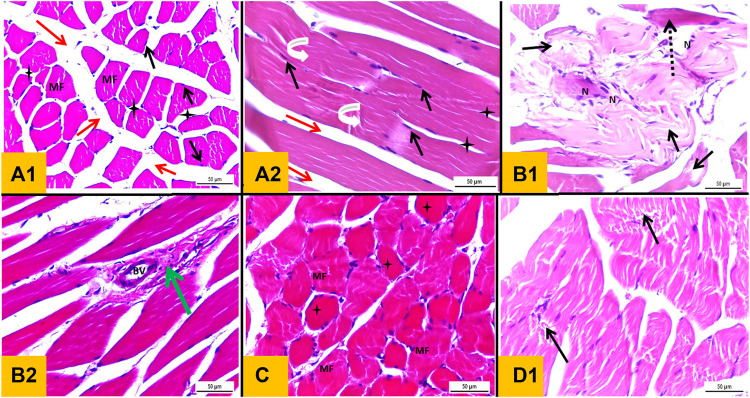
**(A1)**: Haematoxylin and eosin stained section from gastrocnemius muscle (T.S) of a Control group showing polyhedral muscle fibers (MF) with acidophilic sarcoplasm and multiple peripheral oval nuclei (black arrow) separated by narrow C.T. endomysium (star). A wide C.T. perimysium (red arrow) is present between skeletal muscle bundles. **(A2)**: L.S section of gastrocnemius muscle of the Control group showing elongated cylindrical unbranched muscle fibers with acidophilic sarcoplasm and transverse striations (curved white arrow) with multiple oval peripheral nuclei (black arrow). **(B1** and **B2)**: transverse section from gastrocnemius muscle of diabetic group showing morphological damage and a reduction in the number of muscle fibers, wide splitting of the skeletal muscle fibers in association with fibrillolysis (black arrow). Nuclei were internal in position rather than peripheral with crowdies (nuclear clump) (N). Loss of striation in some fibers (interrupted arrow), congested dilated blood vessel (BV) and mononuclear cellular infiltration were observed (green arrow). **(C)**: T.S section from Telmisartan group showing normal muscle fibers with peripheral nuclei (MF) with Loss of striation in some fibers were observed (star). **(D)**: L.S sections from metformin group showing ameliorative effect with minimal splitting of the skeletal muscle fibers with fibrillolysis were detected (black arrow).

Sections of the Control group (group I) stained with H&E showed normal histological architecture of skeletal muscle with preserved fascicular pattern and clear striation. It was made up of muscle fiber bundles separated by connective tissue (perimysium) (Figure 6). A connective tissue network held the fibers together (endomysium). Skeletal muscle fibers appeared polygonal in cross section. But at the other hand, the skeletal muscle fibers in the longitudinal section appeared long, multinucleated, cylindrical, and parallel with a slight variance in fiber size. The sarcoplasm was cross-striated and had an acidophilic appearance and under the sarcolemma, the nuclei were elongated and positioned peripherally (Figures 6A1, A2). Sections of skeletal muscle from Diabetic group (group II) exhibited observable histological alterations in the form of morphological damage, a decline in the number of muscle fibers, fibrillolysis, nuclear internalization, and extensive splitting of the skeletal muscle fibers. Some nuclei looked to be rounded rather than oval in shape and some were internal rather than peripheral in position. Vascular congestion and dilation were seen. Cellular infiltration, splitting of some fibers and loss of striations in others were detected (Figures 6B1, B2). The section obtained from Telmisartan group (group III) elaborated histological picture relatively similar to that of Control group. Normal muscle fibers with peripheral nuclei and some fibers showed loss of striation, minimal fibrillolysis and cellular infiltration (Figure 6C). In sections obtained from Metformin group (group IV) showed ameliorative effect with minimal splitting and fibrillolysis in the skeletal muscle fibers in some sections (Figure 6D).

### 3.9 Masson trichrome stained sections

**FIGURE 7:**
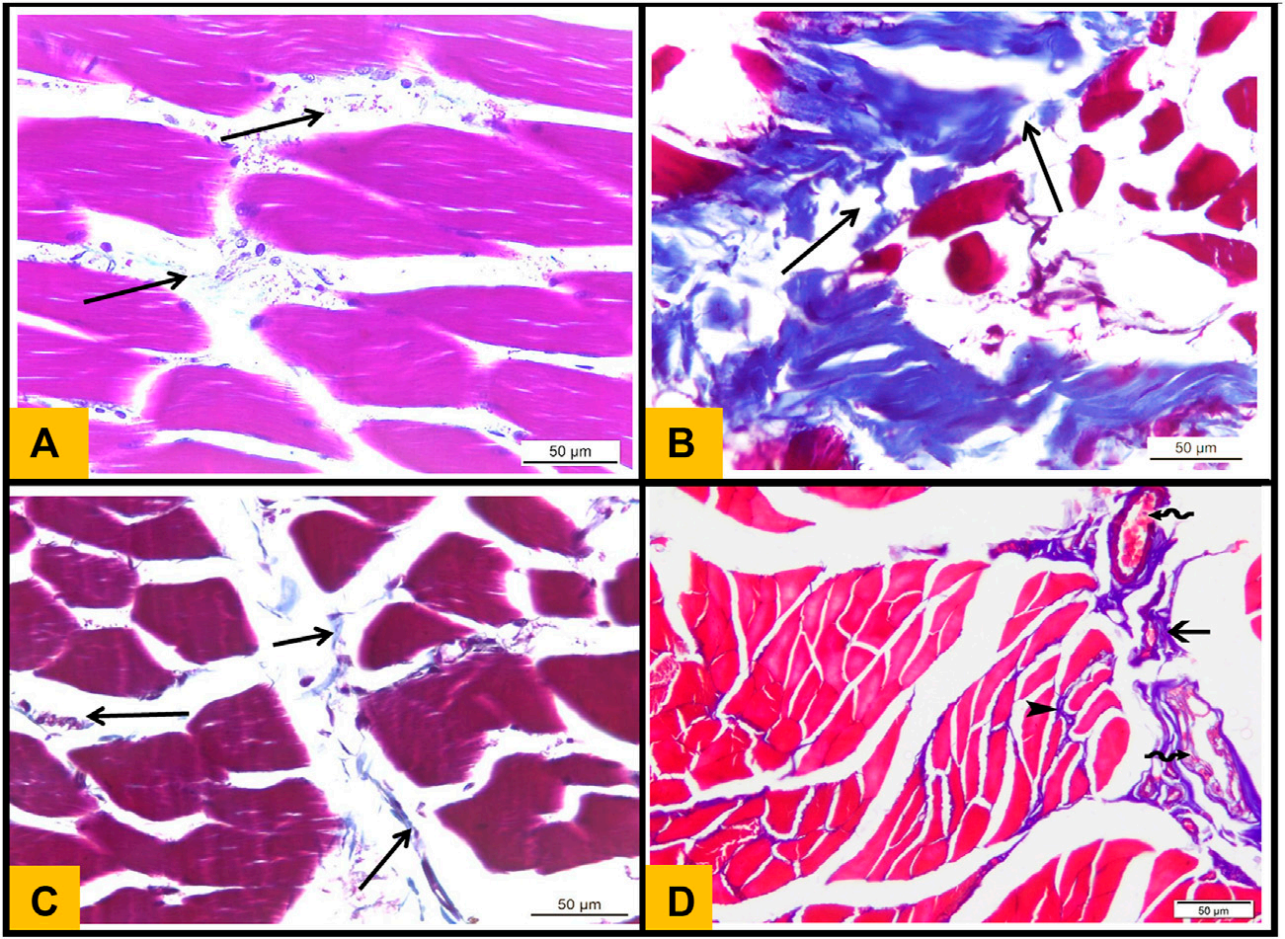
**(A)**: Masson's trichrome stained section (T.S) from Control group showing minimal collagen fibers distribution in perimysium between skeletal muscle bundles (arrows). **(B)**: T.S section from diabetic group showing strong deposition of collagen fibers between the muscle bundles (arrows). **(C)**: T.S section from Telmisartan group showing minimal collagen fibers distribution in between the muscle bundles (arrows). **(D)**: T.S section from the Metformin group showing minimal distribution of collagen fibers in between the muscle fibers (arrowhead) and moderate distribution of collagen fibers between the muscle bundles (arrow)and around multiple congested blood vessels (wavy arrows).

The interstitial connective tissue between the muscle bundles in the control group was very minimal (Figure 7A) (Figure 7). However, the interstitial connective tissue located between muscle fibers and around blood vessels significantly increased in the Diabetic group (Figure 7B). Moreover, Telmisartan and Metformin treated groups showed mild to moderate increase in the interstitial connective tissue surrounding blood vessels and between muscle bundles (Figures 7C,D respectively).

### 3.10 Immunohistochemical examination of (NF-kB)

**FIGURE 8:**
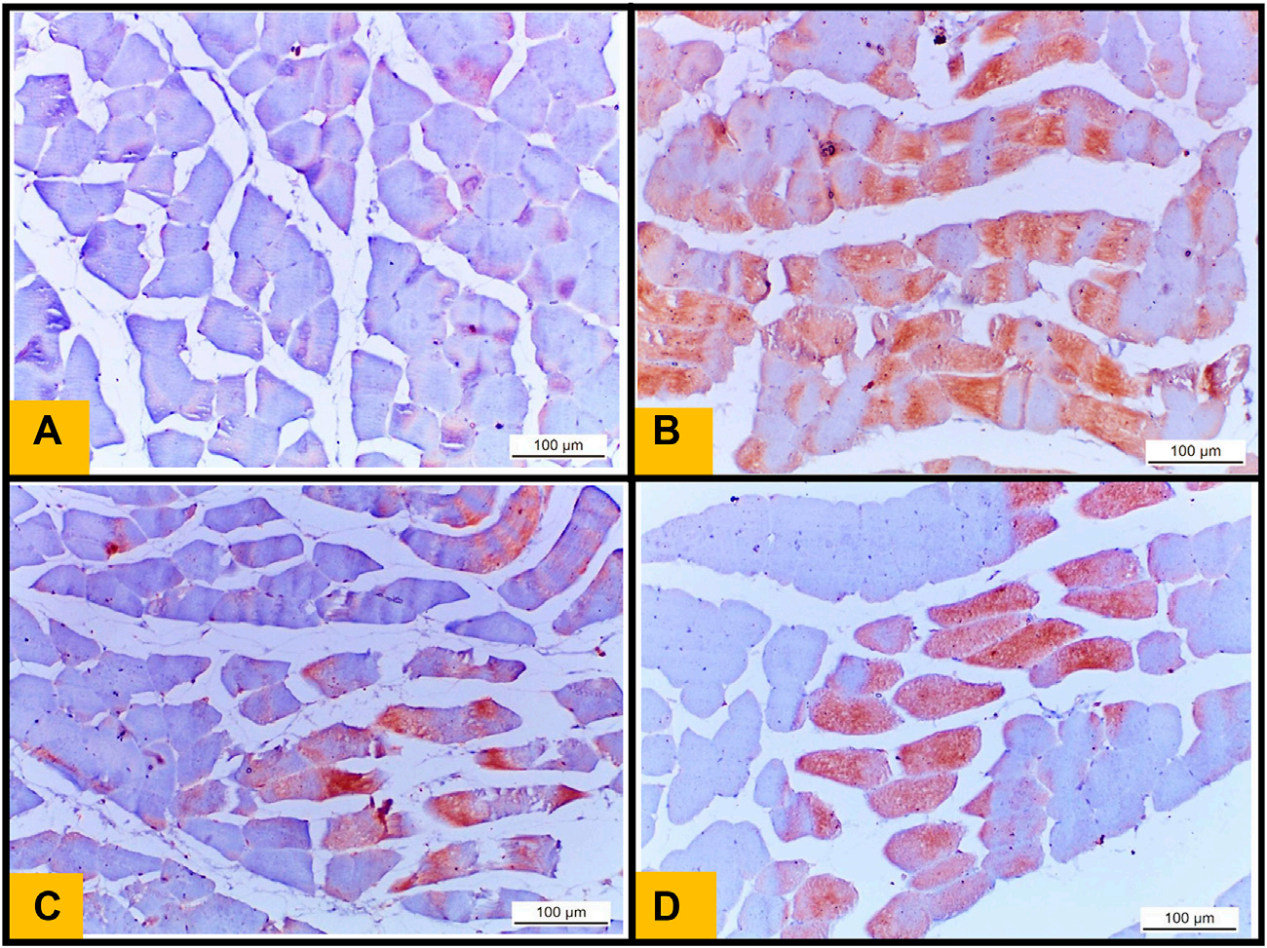
**(A)**: Immunohistochemical staining of nuclear factor kB (NF kB) from the Control group showing no expression of NF kB in the sarcoplasm of muscle fibers. **(B)**: section from diabetic group showing increase in NF kB immunoreactivity in the sarcoplasm of muscle fibers (Brown color) indicates NF kB positivity. **(C)**: section from Telmisartan group demonstrating mild expression of NF kB in the sarcoplasm of some muscle fibers (brown color). **(D)**: section from Metformin group demonstrating reduction in NF kB expression (mild to moderate positivity) in the sarcoplasm of muscle fibers (brown color).

Control group showed no expression of (NF-kB) in the sarcoplasm of muscle fibers (Figure 8A) (Figure 8). While the sections obtained from Diabetic group showed strong positive immunoreactivity in the sarcoplasm of muscle fibers (Figure 8B). Telmisartan group showed mild expression of NF-kB in some sections (Figure 8C). While Metformin group demonstrated mild to moderate NF-kB expression in the sarcoplasm of muscle fibers (Figure 8D).

### 3.11 Area percentage of collagen fibers deposition and NF-kB immunoreactivity

**FIGURE 9:**
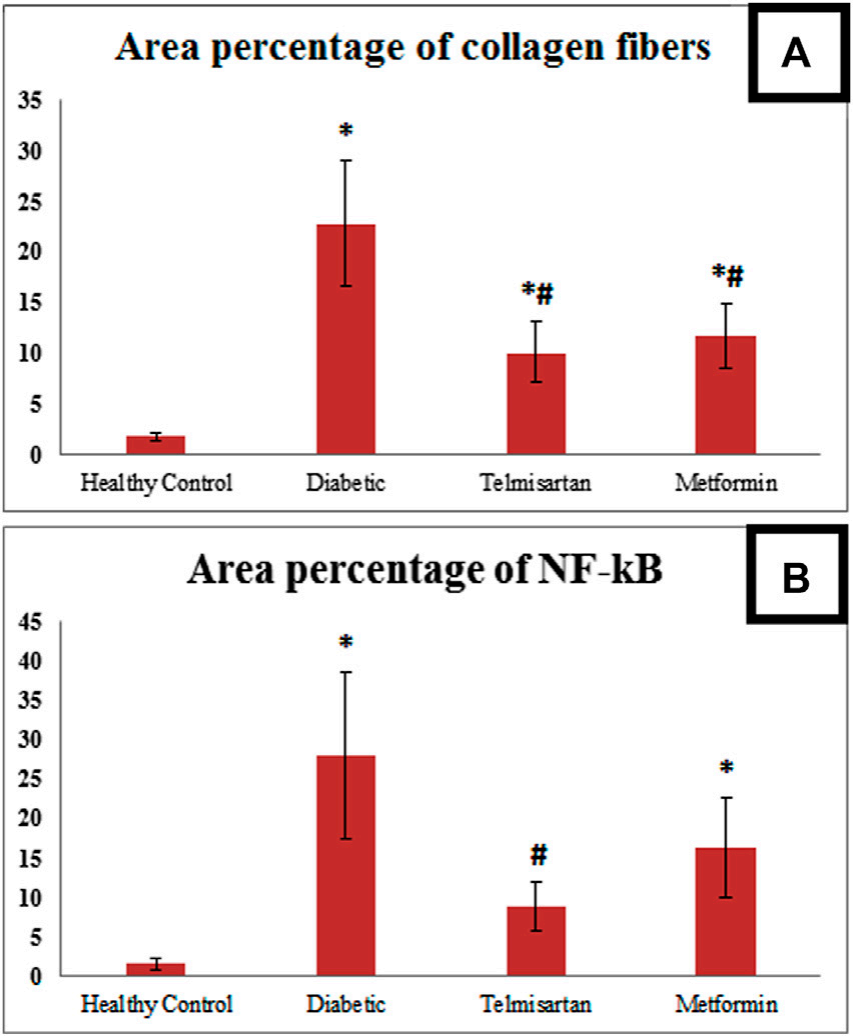
**(A)**: Area percentage of collagen fibers in muscle tissue of all studied groups. **(B)**: Area percentage of NF kB expression in muscle tissue of all studied groups. Values are represented as mean ± SD (n = 8/group). *: *p < 0.05* compared to the Control group, #: *p < 0.05* compared to the Diabetic group, and $: *p < 0.05* compared to the Telmisartan group.

The Diabetic group showed a statistically significant increase of the area percentage of collagen fibers deposition compared to the Control group (Figure 9). While the treated groups either with telmisartan or metformin showed a statistically significant reduction of the area percentage of collagen fibers deposition compared to the Diabetic group. Nonetheless, a substantial difference still existed between the treated groups (groups III and IV) and Control group (*p* < 0.05) (Figure 9A).

Area percentage of NF-kB immunoreactivity in the Diabetic group significantly enhanced compared to the Control group. Telmisartan treatment caused a statistically substantial decline in the area percentage of NF-kB immunoreactivity compared to the Diabetic group with no statistically significant difference as compared to Control rats (*p* > 0.05). Metformin treated group showed an improvement of the area percentage of NF-kB immunoreactivity with a significant difference still noticed between this group and Control group (*p* < 0.05) (Figure 9B).

## 4 Discussion

Telmisartan is a potential candidate to be an effective oral hypoglycemic drug for the management of prediabetes and T2DM, especially when associated with other comorbidities such as hypertension, atherosclerosis, and diabetic nephropathy or when there are severe side effects or contraindications to the other oral hypoglycemic drugs like metformin and other biguanides (Ayza et al., 2020). We should pay a special interest in determining the precise mechanism by which telmisartan can lower blood glucose levels. Despite a dozen studies elucidating the anti-diabetic properties of telmisartan, to the best of our knowledge, the current work is the first to inspect the impact of telmisartan administration on myostatin gene expression in the skeletal muscles of type 2 diabetic rats.

Our findings revealed that both telmisartan and metformin therapy in Groups III and IV, respectively, significantly declined (*p < 0.05*) the serum level of insulin and improved (*p < 0.05*) HOMA-IR, as well as both the fasting and the excursion of blood glucose levels following the oral loading glucose dosage in OGTT as compared to Diabetic rats (Group II) with no significant differences between Groups III and IV. (Salama et al., 2013). confirmed our findings by demonstrating a significant increase in insulin responsiveness, improvement in HOMA-IR, and reduction in the blood levels of glucose in OGTT in T2DM rats treated with telmisartan. However, contrary to our results, they showed that the Telmisartan group had a significantly higher level of serum insulin than the diabetic group did. Another study also showed that telmisartan therapy increased the insulin level in blood (Fouad et al., 2010), which could be explained by suppressing the mRNA expression of the components of renin-angiotensin system (RAS), NADPH oxidase, transforming growth factor-β1 (TGF-β1), and vascular endothelial growth factor (VEGF) in pancreas of type 2 diabetic animals, and all of these changes may increase the insulin release and its serum levels (Hasegawa et al., 2009).

In agreement with our findings, (Goyal et al., 2011), elucidated that telmisartan was able to attenuate the hyperinsulinemia and hyperglycemia in STZ-induced type 2 diabetic rat models. They also demonstrated that Ang-II can inhibit the initial cascades in the insulin signaling pathway, thus the blocking of AT1 receptors can prevent the potential decrease in GLUT-4. ARBs can also upregulate the expression of GLUT-4 gene in the myocardium and skeletal muscles. Moreover, telmisartan is similar in structure to pioglitazone; hence its hypoglycemic effect may be attributed to several mechanisms including increasing the insulin sensitivity and affecting the expression of PPAR-γ target gene.

According to (Fujisaka et al., 2011) telmisartan has several mechanisms that make it more effective than other ARBs in terms of improving glucose metabolism and enhancing insulin sensitivity. The dual action of telmisartan as AT1R blocker and PPAR-γ partial agonist potentiates its efficacy in terms of improving glucose metabolism and enhancing insulin sensitivity. It can retain only the positive benefits of PPAR-γ activation, avoiding salt and water retention and the subsequent weight gain that can be caused by full receptor agonism. Additionally, telmisartan improves insulin sensitivity by a thiazolidinedione-like action, via alterations in adipocyte differentiation and enhancing adiponectin secretion following the stimulation of PPAR-γ beside its ability to reduce the overall adipose tissue mass hence limiting the body weight gain (Zanchi et al., 2007; Fujisaka et al., 2011).

Our results demonstrated that telmisartan administration in group III significantly reversed (*p < 0.05*) the lowered serum adiponectin level in the Diabetic group (group II), with no significant difference between its level in group III and Control group. Nevertheless, metformin failed to increase the adiponectin level in group IV, with no significant difference between its serum levels in groups II and IV. These findings were consistent with previous studies (Salama et al., 2013), (Zhao et al., 2013) which reported that telmisartan administration reduced the average size of adipose tissue while increasing adiponectin secretion and expression in both subcutaneous abdominal and omental fat due to its selective PPAR-γ activity. Moreover, telmisartan considerably boosted the gene expression of AdipoR1 and AdiopR2, the receptors that mediate the anti-diabetic effect of adiponectin, in the liver and adipose tissues. Hence, the low serum level of adiponectin, which is commonly detected in type 2 DM, hypertension, and metabolic syndrome, can be considered as a good indicator for adipocyte malfunction, which precedes glucose intolerance and insulin resistance. Adiponectin has been suggested to act as an “adipostat” with anti-inflammatory and insulin sensitizing properties (Esteve et al., 2009).

Regarding the proinflammatory cytokines, interleukin-6 (IL-6) is a crucial adipocytokine that is released from several tissues, such as adipose tissue, liver cells, and skeletal muscles (Andersson et al., 2008). Obese individuals have a higher serum level of IL-6, which is correlated with the development of metabolic syndrome, insulin resistance, and T2DM (Juge-Aubry et al., 2005) due to the capability of IL-6 to downregulate the gene expression of GLUT-4 and IRS-1 (Rotter et al., 2003). Tumor necrosis factor-α (TNF-α) is another key adipocytokine implicated in the development of insulin resistance in obesity. It plays a significant role in boosting the gene expression of IL-6 and regulating the liver’s synthesis of C-reactive protein (Blake and Ridker, 2001). Interleukin-10 (IL-10) is a crucial anti-inflammatory cytokine used by immune cells to inhibit inflammation. It is thought to be an pivotal cytokine in the cross-talk between the immune system and adipose tissue. Several studies have shown that IL-10 has a vital role in suppressing the development of insulin resistance as well as regulating the transcription of thermogenic genes in white adipose tissue (Clementi et al., 2009), (den Boer et al., 2006), (Rajbhandari et al., 2018).

The current study showed that telmisartan administration in group III has a potent anti-inflammatory effect, significantly lowering (*p* < 0.05) the serum levels of the proinflammatory biomarkers TNF-α and IL-6 while significantly increasing (*p* < 0.05) the serum levels of the anti-inflammatory IL-10 in Telmisartan group (group III) compared to the Diabetic group (group II). Telmisartan was shown to be more effective than metformin in ameliorating the changes in the serum levels of the aforementioned inflammatory cytokines. Our results matched those of (Schaalan, 2012) who elucidated that telmisartan significantly suppressed TNF-α and IL-6 expression in subcutaneous abdominal and omental fat. Hence, they concluded that telmisartan can exert its anti-inflammatory action through downregulating the inflammatory cytokines and upregulating adiponectin in adipocytes. The anti-inflammatory actions of telmisartan can be attributed to the partial stimulation of PPAR-γ and blocking of AT1R. Besides the favorable remodeling impact of telmisartan on adipose tissues that improves the serum patterns of the inflammatory adipocytokines as they are primarily generated from adipocytes. Moreover, telmisartan can alter the storage profile of adipocytes as well as normalize the size of adipose tissue. All of these mechanisms work in tandem with the activation of PPAR-γ, which causes an increase in adiponectin expression and, as a result, an improvement in insulin sensitivity (Schaalan, 2012)**.**

Concerning the oxidative stress indicators, malondialdehyde (MDA) is a lipid peroxidation and tissue injury biomarker, and its high content in tissues indicates enhanced activity of phospholipase enzyme and excessive generation of lipid peroxyl radicals (Abd-Eltawab Tammam et al., 2022). Glutathione (GSH) is a substantial non-enzymatic antioxidant which works with the other antioxidant enzymes in the cells like superoxide dismutase (SOD) to remove the reactive oxygen species (ROS) and to safeguard the tissues against oxidative damage (Noshy et al., 2022), (Alaa et al., 2018).

Our results revealed that telmisartan has a potent antioxidant effect, as evidenced by a substantial decline (*p* < 0.05) in MDA concentration in the Telmisartan group and a significant rise (*p* < 0.05) in the GSH content and SOD activity in the skeletal muscle tissue homogenate of the Telmisartan group (group III) compared to the Diabetic group (group II). These findings can be explained by the proven ability of angiotensin II to enhance the formation of superoxide radicals, which combine with nitric oxide to generate peroxynitrite, resulting in a decrease in the level of free nitric oxide, which contributes to the development of an oxidative stress state that increases MDA content, suppresses SOD activity, and consumes GSH content in tissues (Goyal et al., 2011). Hence, the ability of telmisartan to diminish the oxidative damage and to improve the oxidative stress indicators in tissues can be referred to its AT1R blocking activity and interfering with the action of angiotensin II (Pacher et al., 2005).

Although glucose homeostasis is a multifactorial process in which many items are involved, including the dietary ingredients, GI microbiota, adipose tissue and hepatic metabolic conditions, alterations in skeletal muscle dynamics remain the cornerstone of the early metabolic perturbations that lead to the development of type 2 DM (Roden, 2015). Skeletal muscles’ bulking up and contraction can attenuate the resistance to insulin, resulting in massive glucose uptake through the phosphatidylinositol-3-kinase (PI3K)/protein kinase B (AKT) insulin signaling pathway that plays a crucial role in the phosphorylation of ISR-1, translocation of GLUT-4 into the cell membranes, moving blood glucose into skeletal muscles and Controlling blood glucose levels (Mohamed et al., 2018), (Gandhi et al., 2014).

The elevated levels of myostatin were proved to be associated with obesity and T2DM as it initiates insulin resistance in both human and mouse models. It has been found to reduce basal and insulin-induced IRS-1 tyrosine (Tyr495) phosphorylation, and the expression and provocation of PI3K, associated with declined AKT phosphorylation. Moreover, it inhibits the expression of GLUT-4 mRNA and protein and decreases the translocation of GLUT-4 to the membrane, thus decreasing the glucose uptake (Liu et al., 2018). Our findings revealed diminished gastrocnemius muscle glucose uptake in the Diabetic group that was significantly improved following the treatment with both telmisartan and metformin. This was in line with (Li et al., 2013) who demonstrated that telmisartan could improve insulin sensitivity and enhanced insulin-stimulated glucose uptake both *in vivo* in the skeletal muscles of mice and *in vitro* in cultured myotubes.

We also revealed a decline in muscle mass to body weight ratio in skeletal muscles that was in line with decrease in gastrocnemius glucose uptake in the Diabetic group compared to Control rats, while both telmisartan and metformin treatment have significantly improved the ratio with superior role of telmisartan over metformin. Moreover, we demonstrated enhanced gastrocnemius myostatin gene expression in diabetic group which was significantly downregulated after telmisartan treatment even more than metformin treatment. An accumulating body of evidence proposes that the serum level of circulating myostatin reflects the level of myostatin in the muscles and has several pathophysiological impacts. (Ju and Chen, 2012). Previous research works have demonstrated that myostatin suppression has diverse therapeutic influences on muscle atrophy, reduced adiposity with enhanced response to insulin (LeBrasseur et al., 2009; Camporez et al., 2016). Previously reported that a rise in the serum level of myostatin in obese individuals is correlated to the aggravation of their resistance to insulin in a way that is independent of their skeletal muscle mass (Tanaka et al., 2018).

The current study found a significant downregulation of insulin receptor, IRS-1, and IRS-3 genes expression in skeletal muscles with induced diabetes while treatment with either telmisartan or metformin significantly enhanced their expression. These findings may be explained by what has been reported previously by who found a statistically significant boost in the levels of insulin receptor and IRS-1 proteins as well as their phosphorylated forms in skeletal muscle of pigs with a myostatin loss-of-function mutation (Mstn −/− pigs), which then stimulating the signaling pathway of insulin (Cai et al., 2017). Also demonstrated elevated myostatin levels in muscle and liver of mice as well as circulating myostatin in response to high caloric intake which resulted in increased expression of Casitas B-lineage lymphoma b (Cblb) in a Smad3-dependent manner. Cblb is an ubiquitin E3 ligase that specifically degrades IRS1 protein and induces insulin resistance (Bonala et al., 2016).

Based on the previous findings we hypothesized that the telmisartan has a downregulating effect on myostatin expression resulted in enhancement of the insulin sensitivity in skeletal muscles via upregulating the expression of insulin receptor, IRS-1, and IRS-3 leading to activation of the insulin signaling pathway that increases the glucose uptake by muscles.

Our study showed obvious histological abnormalities in the diabetic group, including damage, decreased number, and fragmentation of the skeletal muscle fibers with nuclear internalization and fibrillolysis. Dilated and congested blood vessels with cellular infiltration were also found. These findings concur with those of (Mukundwa et al., 2016)**,** who examined the impact of streptozotocin-induced diabetes and its impact on the insulin signaling system in rats. (Youssef et al., 2022). reported mononuclear cell infiltration in diabetic skeletal muscles, which is consistent with our findings. In accordance with our results Elsy et al., 2017 reported that reduced myocyte protein synthesis, blood vessel alterations, and motor end plate degradation all contribute to insulin depletion in diabetes.

According to previous studies (Ding et al., 2013; Hameed et al., 2015; Oguntibeju, 2019) these changes may be brought on by oxidative stress, overproduction of reactive oxygen species caused by hyperglycemia, and an imbalance between protein synthesis and breakdown (Reddy et al., 2018)**.**

In the current study, fibrosis has been detected using Masson’s trichrome stained sections. The interstitial connective tissue around blood vessels and between muscle fibers significantly increased in the Diabetic group. This is explained by the reduced activity of the metabolic regulator and cellular bioenergetic sensor AMP activated protein kinase (AMPK) in diabetes mellitus, which causes collagen deposition to accelerate in several organs (Yang et al., 2019; Wang et al., 2021)**.** The prior findings support the existence of skeletal muscle fibrosis due to the inflammatory processes in diabetic rats (Li et al., 2017)**.**

Our findings showed that the NF-kB immunohistochemistry expression was statistically significantly higher in the muscle fiber sarcoplasm of the diabetic group which can be explained by lipid peroxidation. In addition to triggering inflammation in the skeletal muscles, oxidative stress also induces the NF-kB pathway, which results in the generation of iNOS.

Owing to the fact that NF-kB could influence the insulin signaling system and play a role in producing insulin resistance, it is possible that inhibiting NF-kB activity could be a new approach to alleviate this condition (Zhong et al., 2011)**.** A study conducted on rats found that diabetes raised the expression of NF-kB in the liver tissue (Coşkun et al., 2017)**.** In the current study, NF-kB immunohistochemistry in the Diabetic group was found to be increased, after Telmisartan group in particular, a significantly reduced expression of NF-kB in the sarcoplasm of muscle fibers was obvious while Metformin group showed no statistically significant difference compared to the Diabetic group.

Several studies have shown how NF-kB inhibition is important in preventing the atrophy and wasting of the skeletal muscles (Mourkioti and Rosenthal, 2008). Hence we assumed that telmisartan might also inhibit myostatin expression via inhibiting NF-kB signaling. In agreement with our findings, Xiao et al., 2012 demonstrated that liver cirrhotic patients have transcriptionally increased levels of Myostatin by activating an NF-kB-dependent pathway.

## 5 Conclusion

Telmisartan is a widely used antihypertensive medication due to its outstanding safety profile. It has been shown to exert euglycemic effects, so it could be a promising choice due to its dual benefit for hypertensive patients with T2DM. It also has fewer adverse effects compared to metformin and other oral antidiabetic drugs which gives it the superiority over the other drugs. Based on our findings, we concluded that one of telmisartan euglycemic effects could be by downregulating the myostatin gene expression in the skeletal muscles even more effectively than metformin resulting in enhancment of adiponectin release (the insulin-sensitizing adipocytokine). Telmisartan could also improve insulin sensitivity in the skeletal muscles by upregulating the expression of insulin receptors, IRS-1 and IRS-3, leading to activation of the insulin signaling pathway thus increasing glucose uptake by the skeletal muscles. These findings may highlight novel antidiabetic mechanisms of telmisartan in treating T2DM.

In addition, telmisartan administration was able to minimize the activity of pro-inflammatory cytokines, the oxidative stress state, the muscle wasting associated with diabetes, the morphological damage of muscle fibers, collagen deposition, and NF-kB expression as compared to diabetic rats. All of these results confirm our hypothesis that telmisartan’s suppression effect on myostatin may enhance insulin sensitivity, glucose homeostasis, and overall muscle health.

### 5.1 Limitations of the study

More research is needed to confirm the impact of telmisartan on myostatin expression throughout the different tissues, particularly adipose tissue. Furthermore, studies on humans of various races, who may have genetic and environmental variances, are required to corroborate these mechanisms in human patients.

## Data Availability

All data in this study are available from the corresponding author on reasonable request.
