# Supplementary material for: Telmisartan versus metformin in downregulating myostatin gene expression and enhancing insulin sensitivity in the skeletal muscles of type 2 diabetic rat model
Source: Front Pharmacol. 2023 Jul 28;14:1228525. doi: 10.3389/fphar.2023.1228525 (PMC10416801; doi:10.3389/fphar.2023.1228525)
Supplement: Supplementary file 1 [file DataSheet1.PDF]

CONTROL

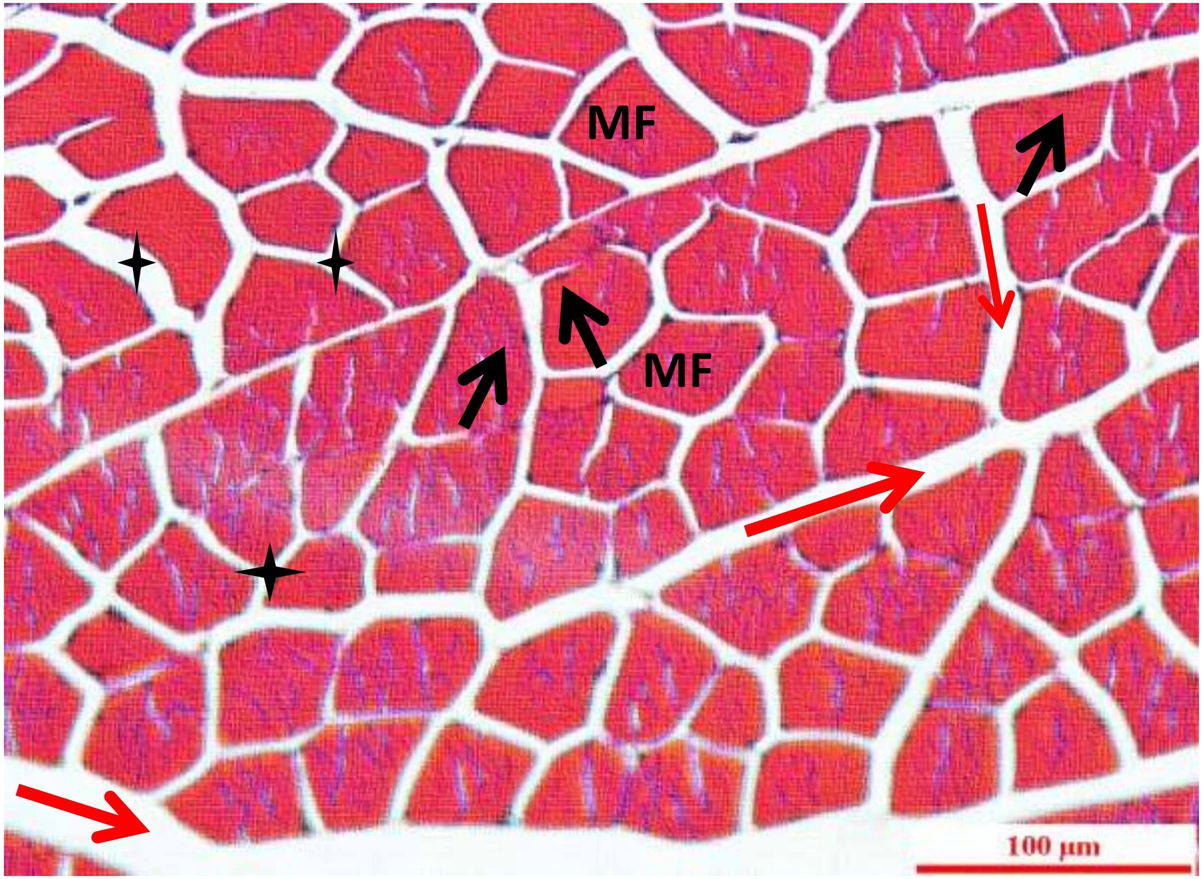
MF
MF
100 µm

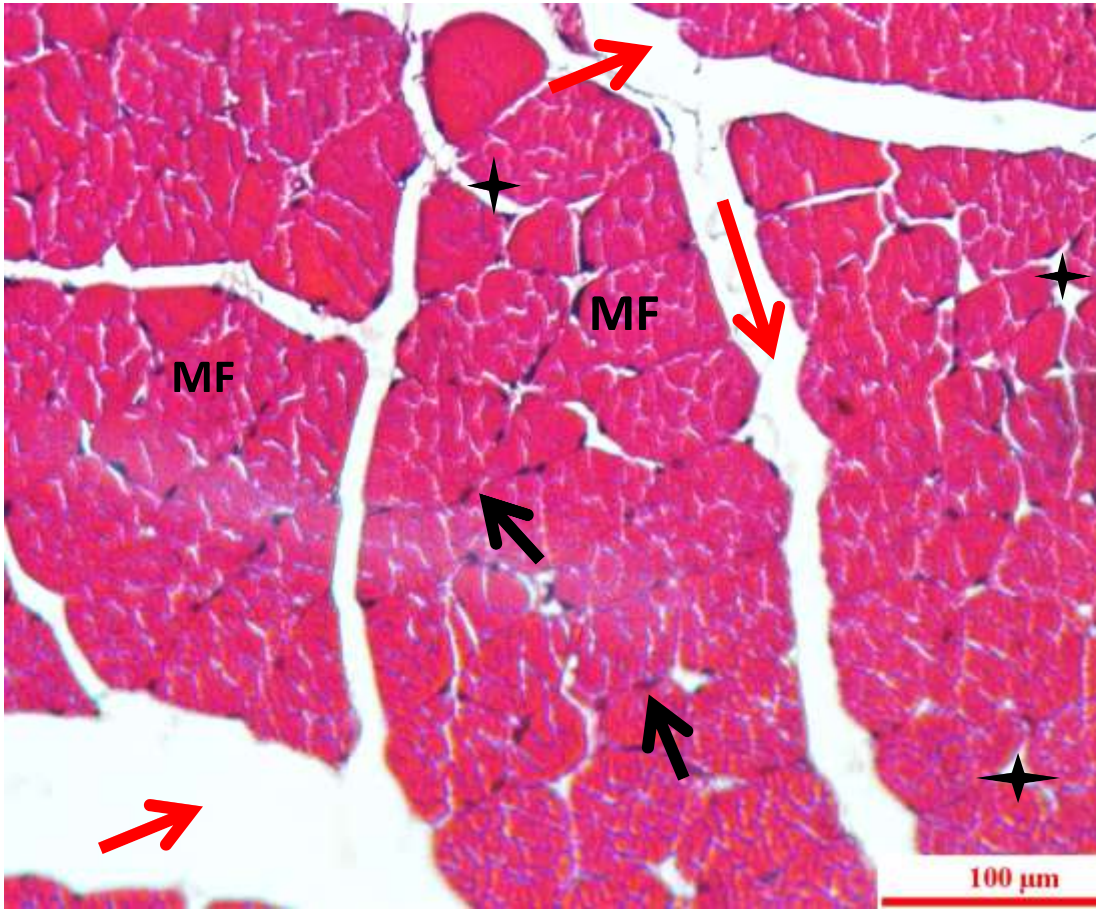
MF
MF
100 µm

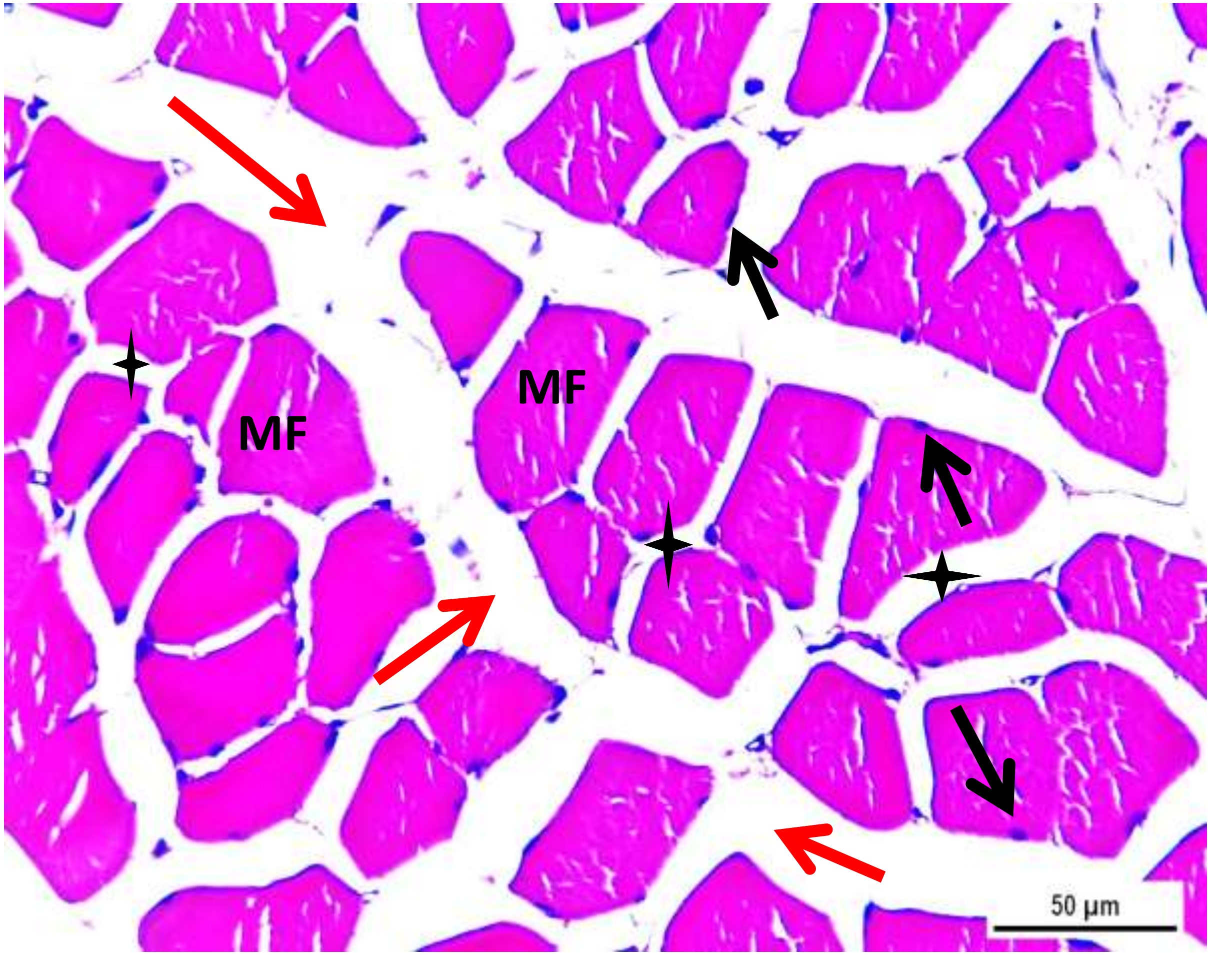
MF
MF
50 µm

Micrograph of a transverse section from gastrocnemius muscle of a control rat (group I) showing polyhedral muscle fibers **(MF)** with acidophilic sarcoplasm and multiple peripheral oval nuclei **(black arrow)** separated by narrow C.T. endomysium **(star).** A wide C.T. perimysium **(red arrow)** is present between skeletal muscle bundles.

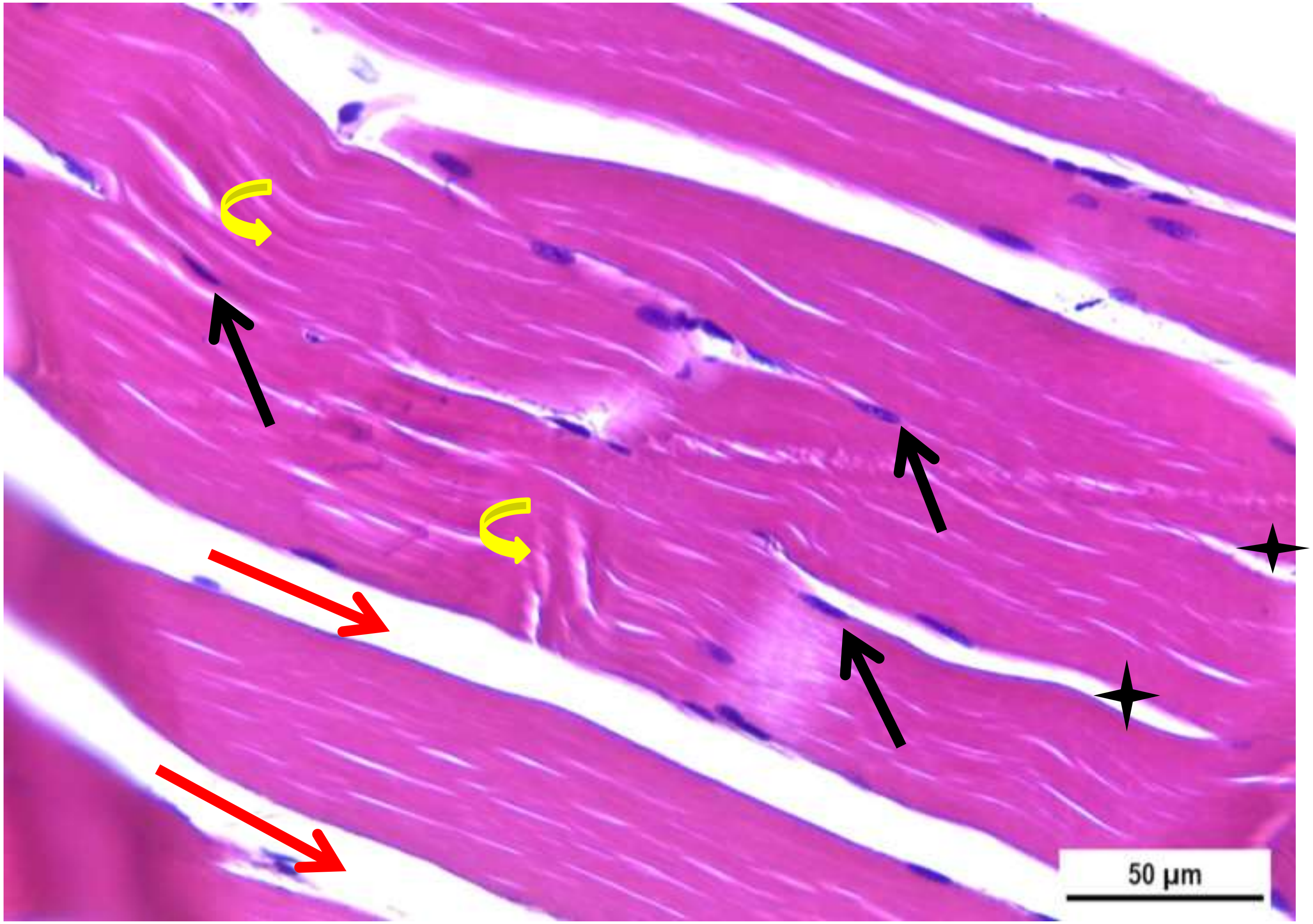
50 µm

Micrograph of a longitudinal section of gastrocnemius muscle of a control rat (group I) showing elongated cylindrical unbranched muscle fibers with acidophilic sarcoplasm and transverse striations **(curved yellow arrow)** with multiple oval peripheral nuclei **(black arrow).** The muscle fibers are separated by narrow C.T. endomysium **(star).** A wide C.T. perimysium **(red arrow)** is present between skeletal muscle bundles.

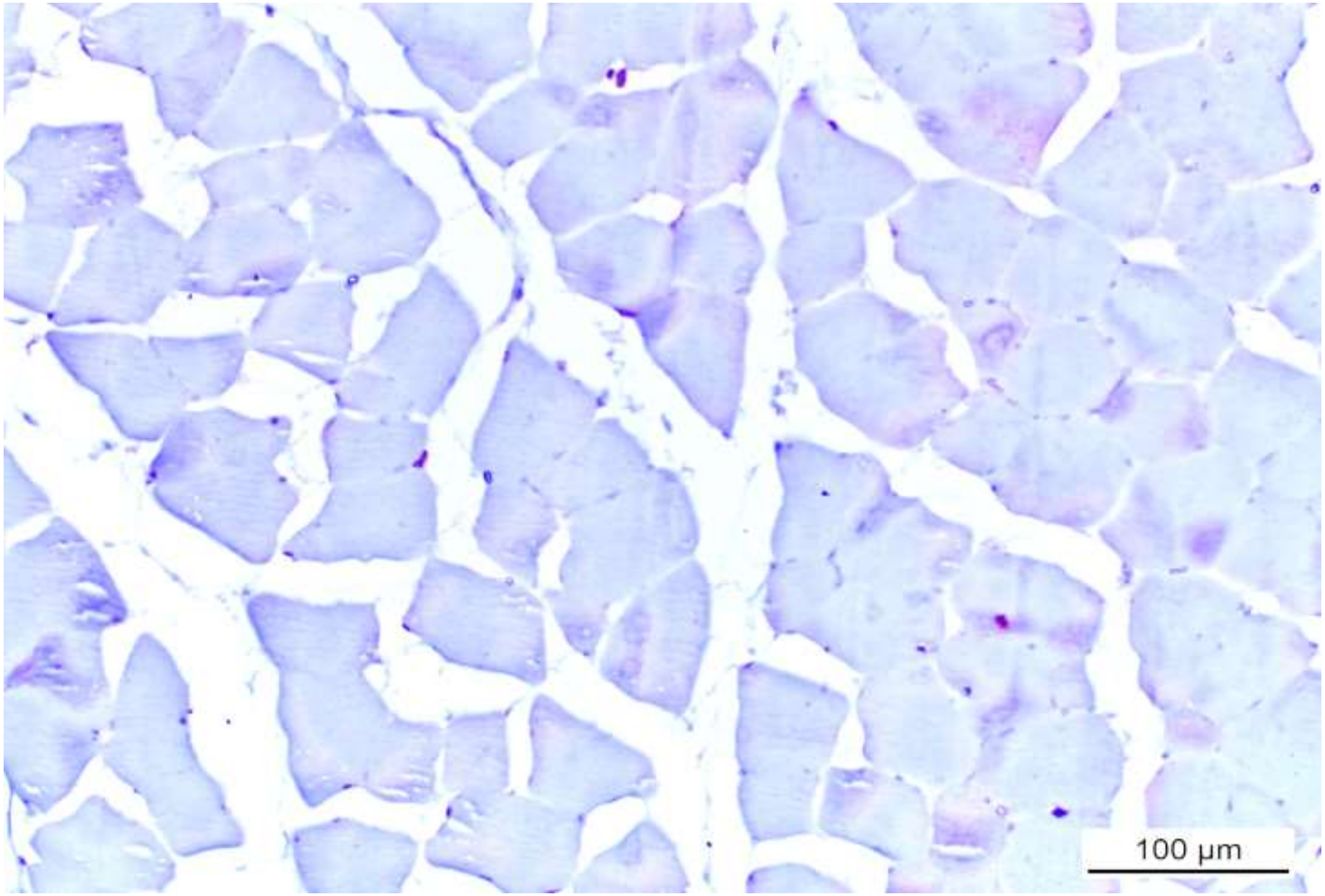
100 µm

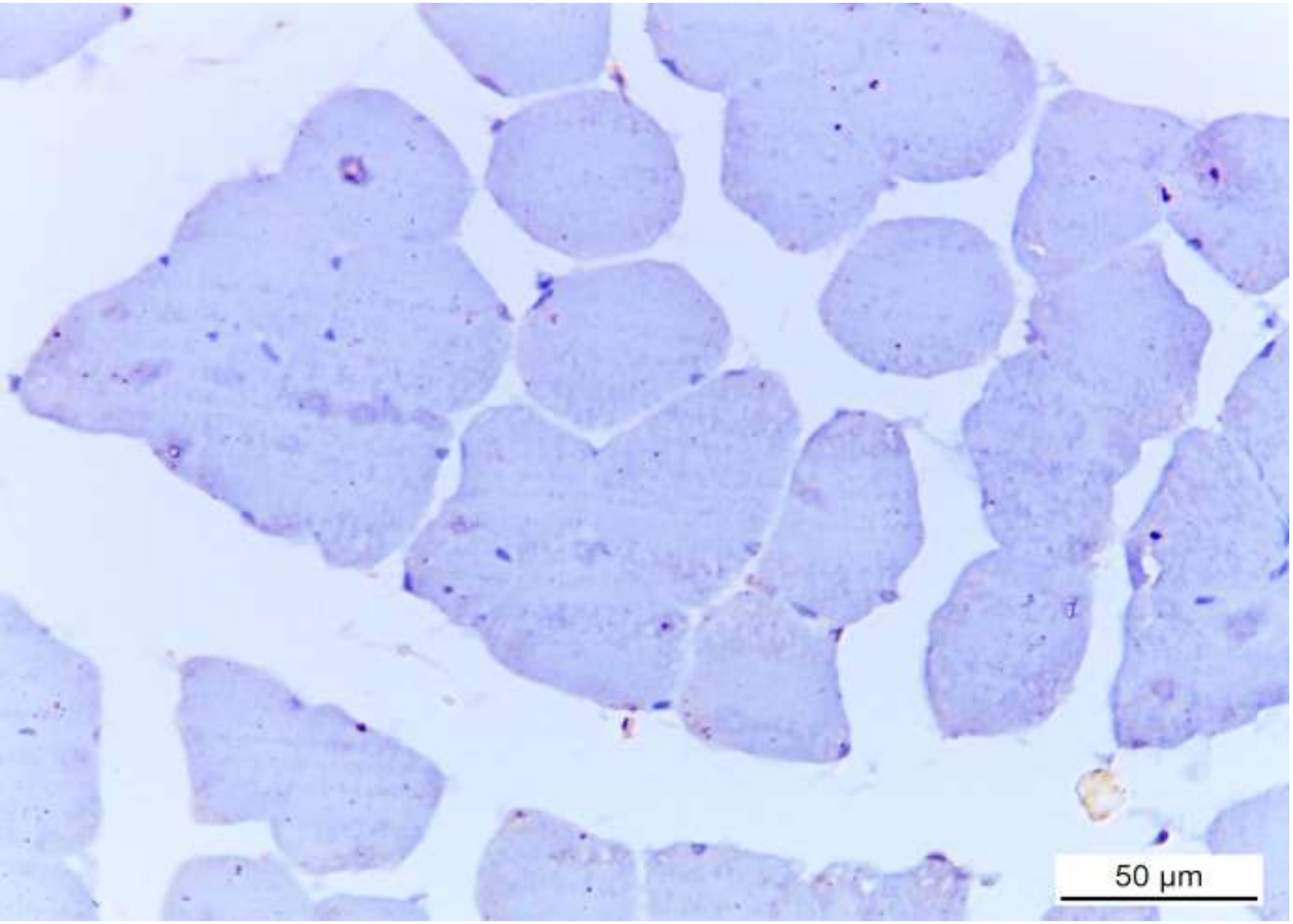
50 µm

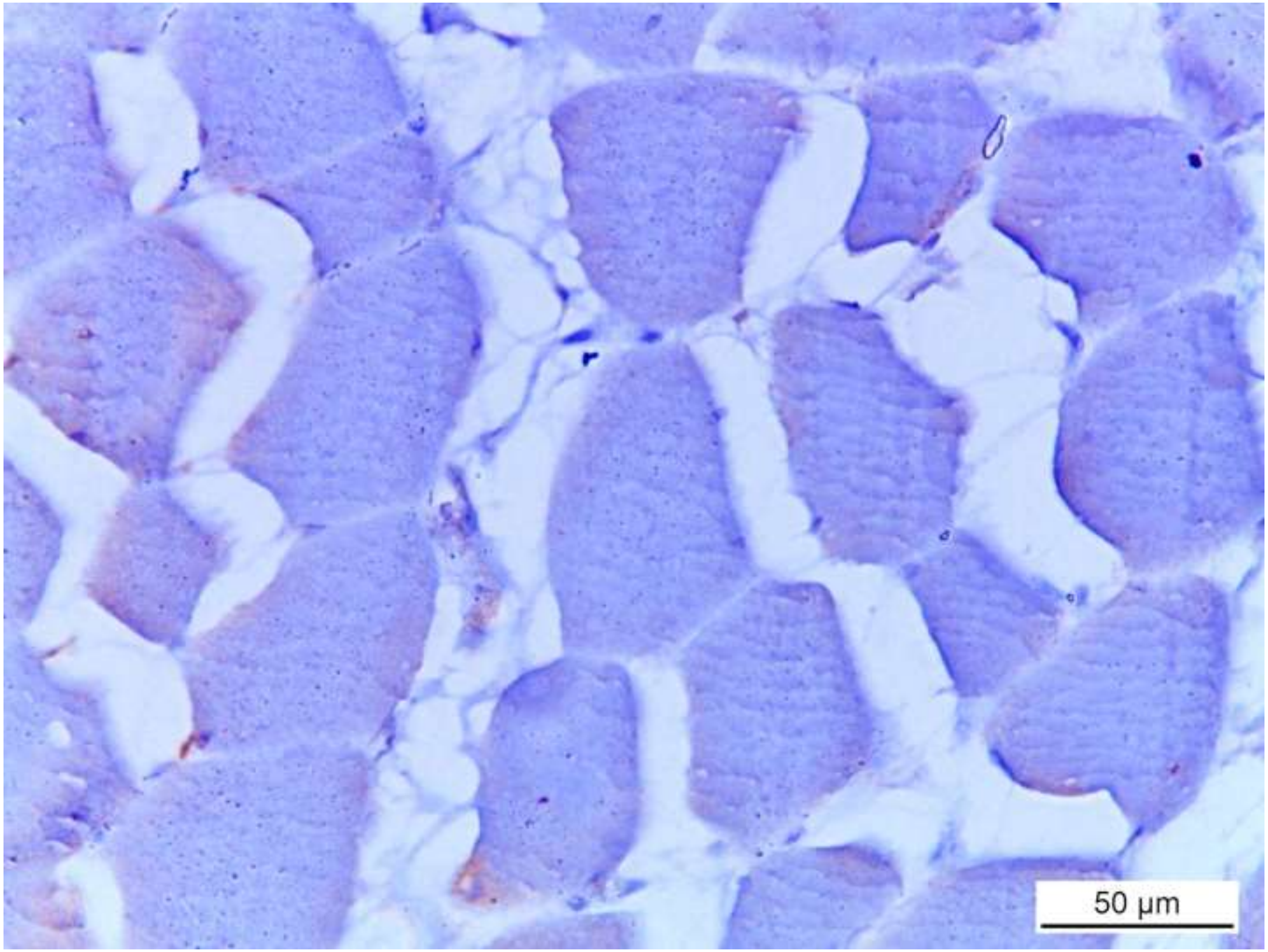
50 µm

Micrograph of a transverse section from gastrocnemius muscle of a control rat (group I) showing Immunohistochemical staining of nuclear factor-kB (NF-kB) showing no expression of (NF-kB) in the sarcoplasm of muscle fibers .

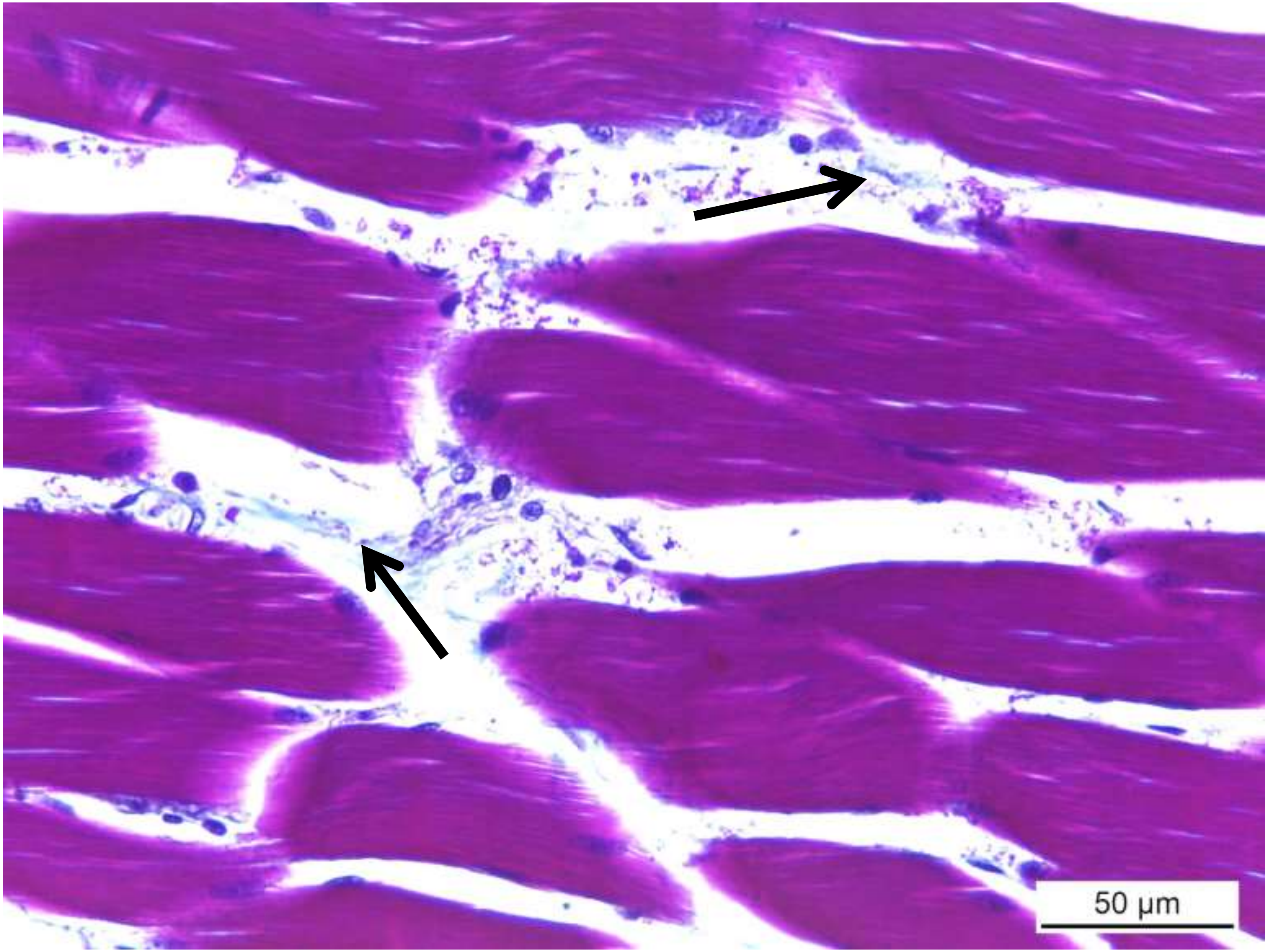

50 µm

Micrograph of transverse section of gastrocnemius muscle of of a control rat (group I) showing minimal collagen fibers distribution in perimysium between skeletal muscle bundles **(arrows).**

Diabetic

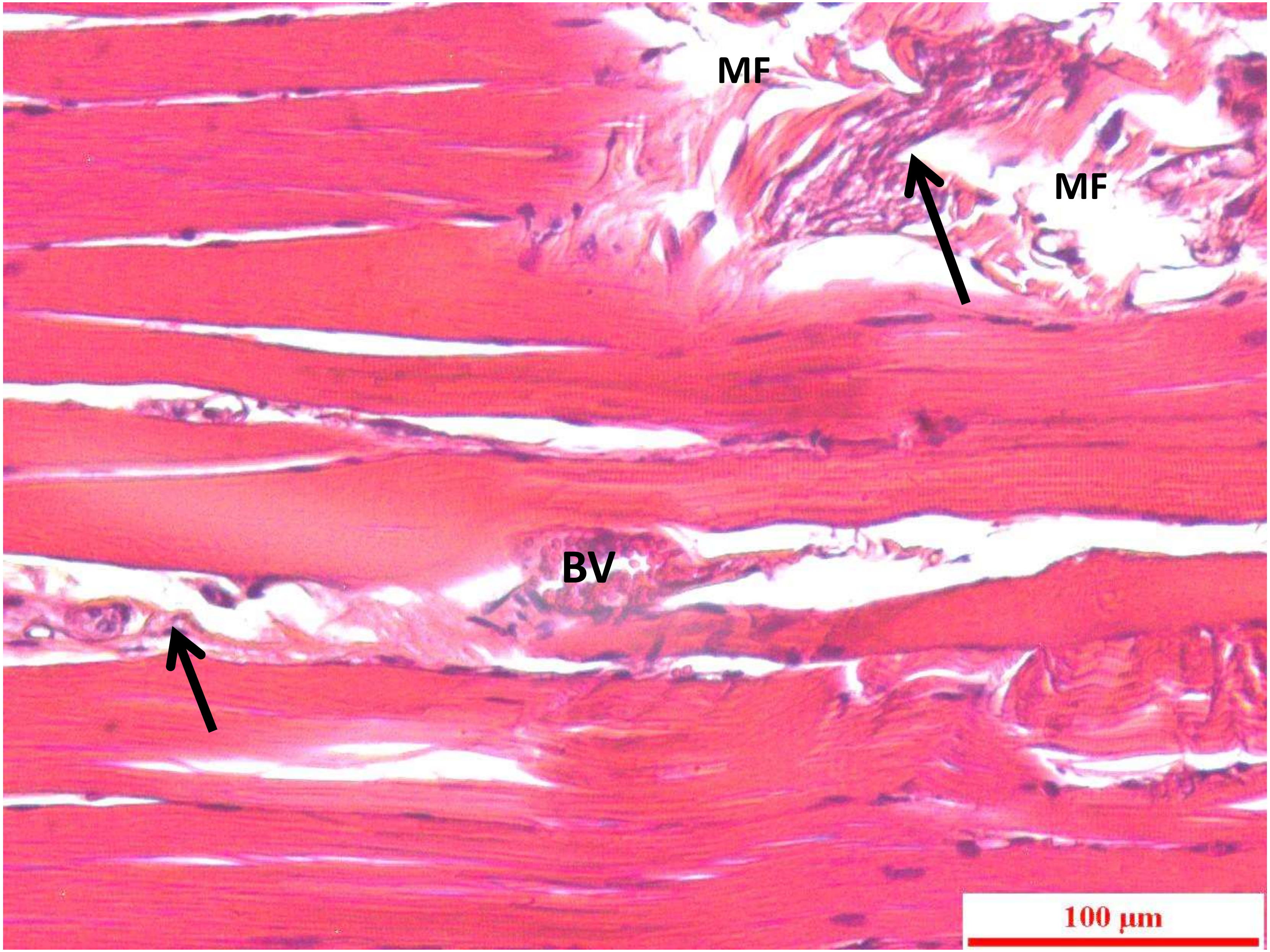
MF
MF
BV
100 µm

Micrograph of longitudinal sections from gastrocnemius muscle of a diabetic rat (group II) showing splitting of muscle fibers **(MF).** Mononuclear cellular infiltration can be noted in CT in-between muscle fibers **(black arrow)** with congested blood vessel **(BV).**

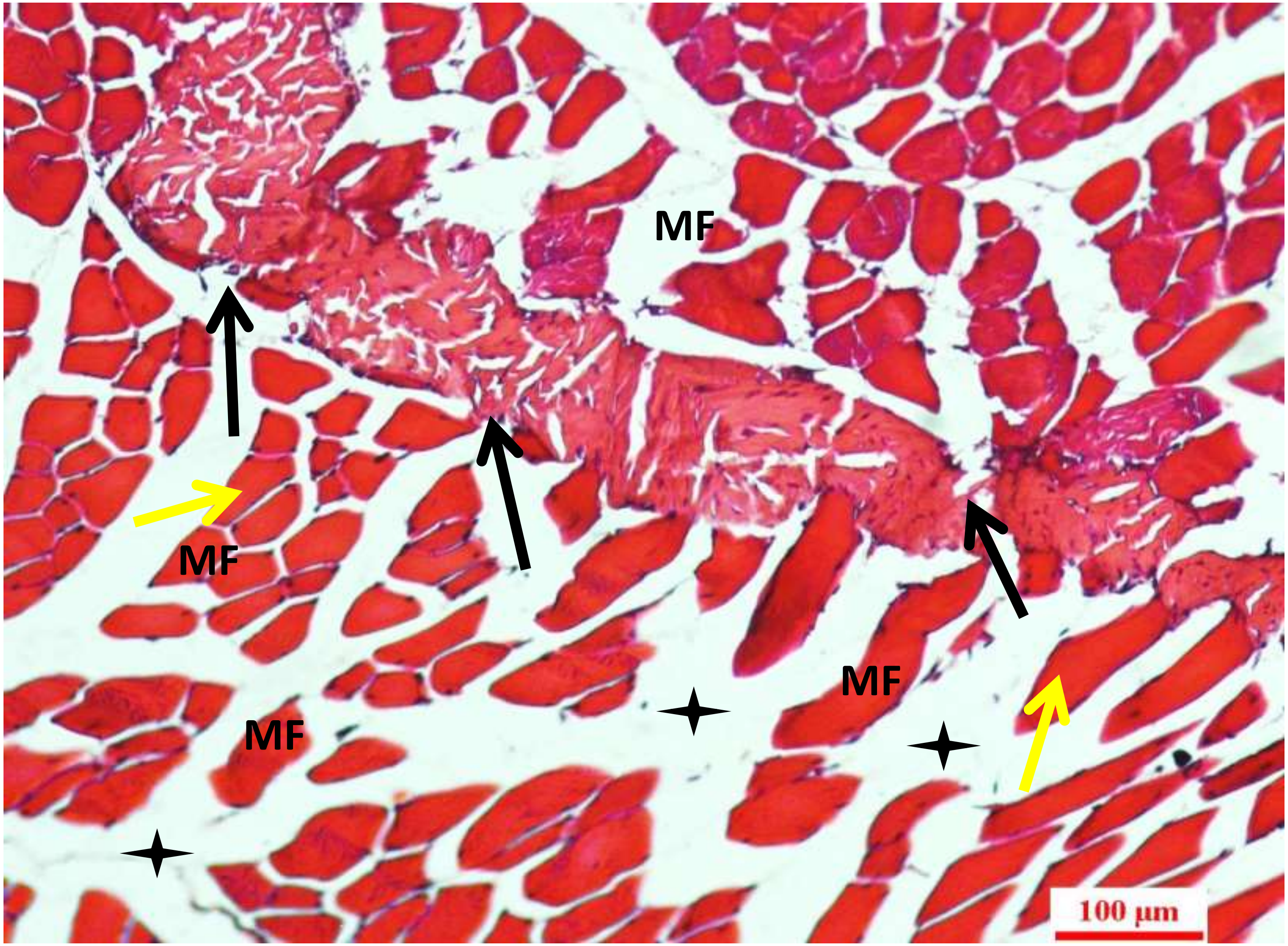
MF
MF
MF
MF
100 μm

Micrograph of transverse section from gastrocnemius muscle of a diabetic rat (group II) showing splitting of the skeletal muscle fibers in association with fibrillolysis **(black arrow).** Muscle fibers showed irregular variation and reduction in size **(MF)** . Loss of striation in some fibers **(yellow arrow).** A wide C.T. perimysium **(star)** is present between skeletal muscle bundles.

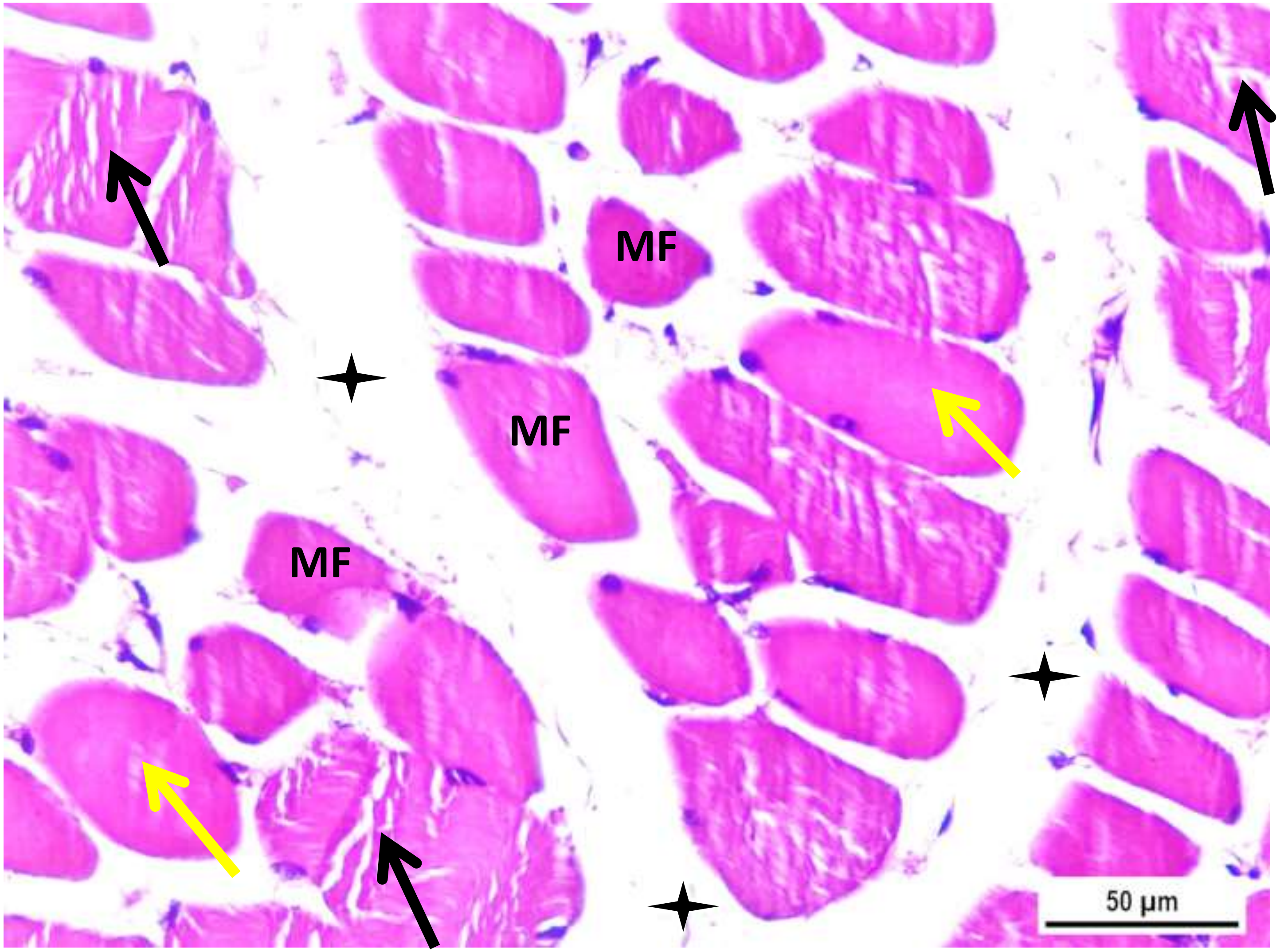
MF
MF
MF
50 µm

Micrograph of transverse section from gastrocnemius muscle of a diabetic rat (group II) showing splitting of the skeletal muscle fibers **(black arrow).** Muscle fibers showed irregular variation in size **(MF)** . Loss of striation in some fibers **(yellow arrow).** wide C.T. perimysium **(star)** is present between skeletal muscle bundles.

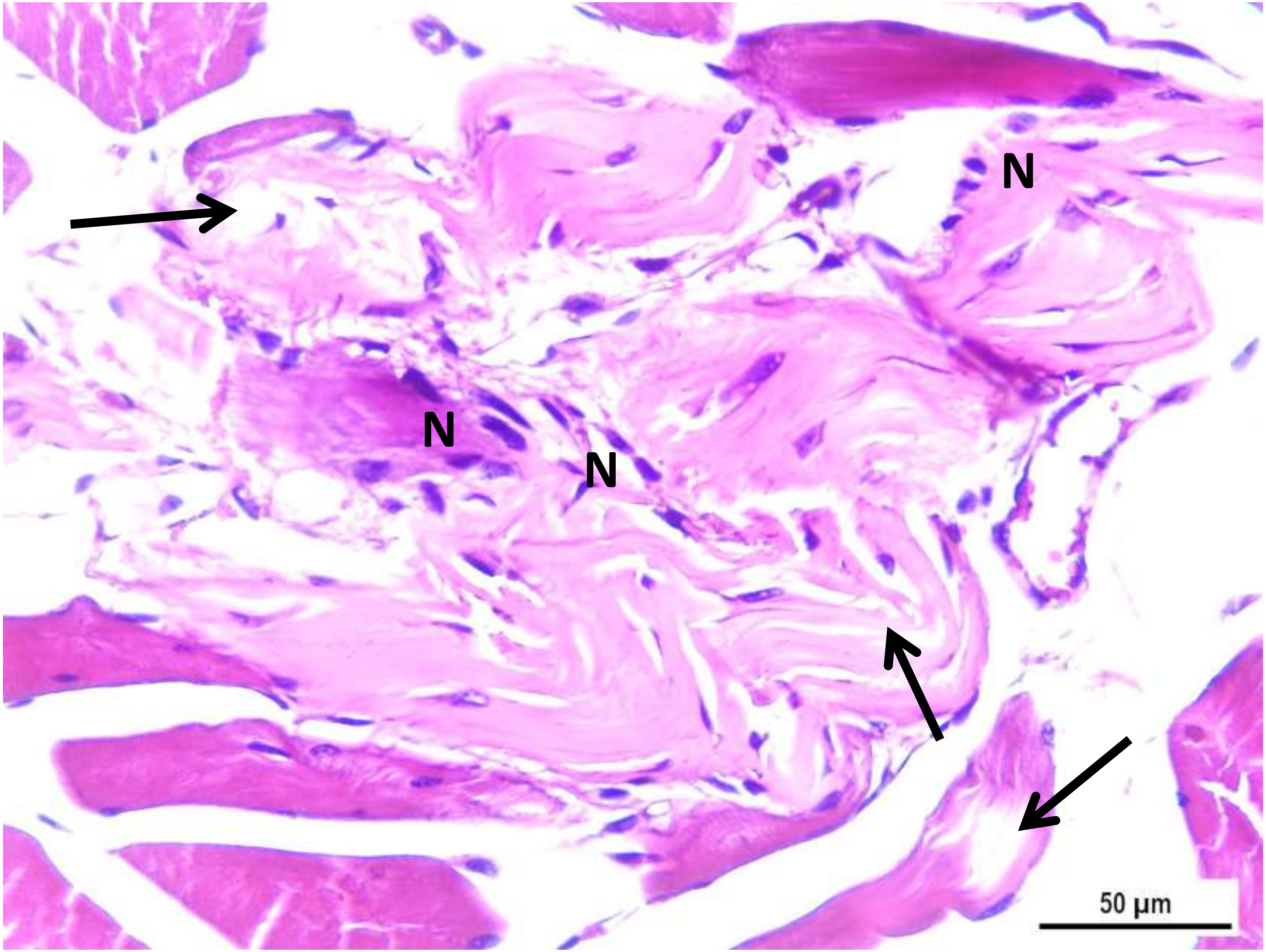
N
N
N
50 µm

Micrograph of transverse section from gastrocnemius muscle of a diabetic rat (group II) showing morphological damage and a reduction in the number of muscle fibers, wide splitting of the skeletal muscle fibers in association with fibrillolysis **(black arrow**). Nuclei were internal in position rather than peripheral with crowdies (nuclear clump) **(N).**

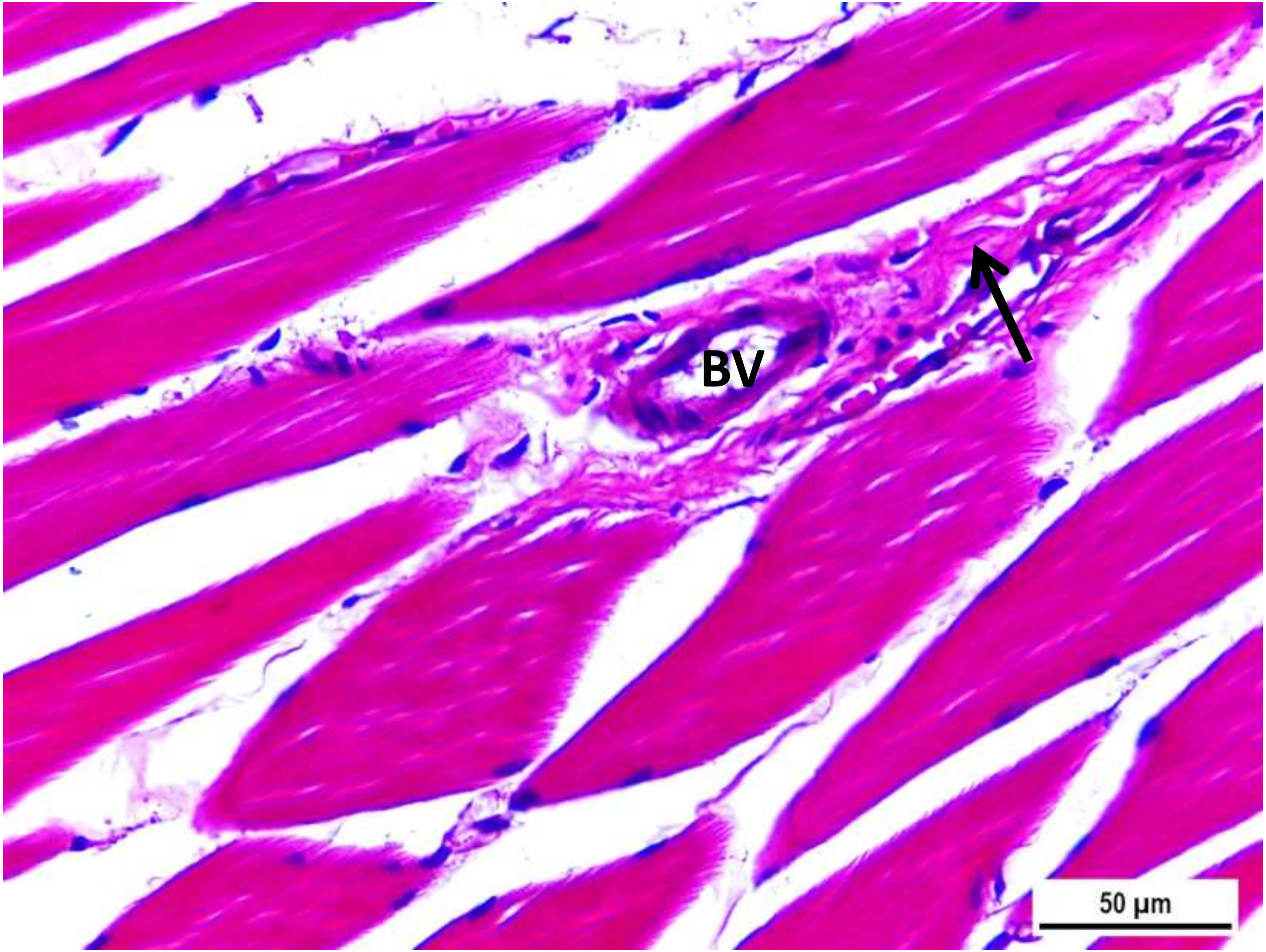
BV
50 µm

Micrograph of transverse section from gastrocnemius muscle of a diabetic rat (group II) showing congested dilated blood vessel **(BV).** Mononuclear cellular infiltration was observed **(black arrow).**

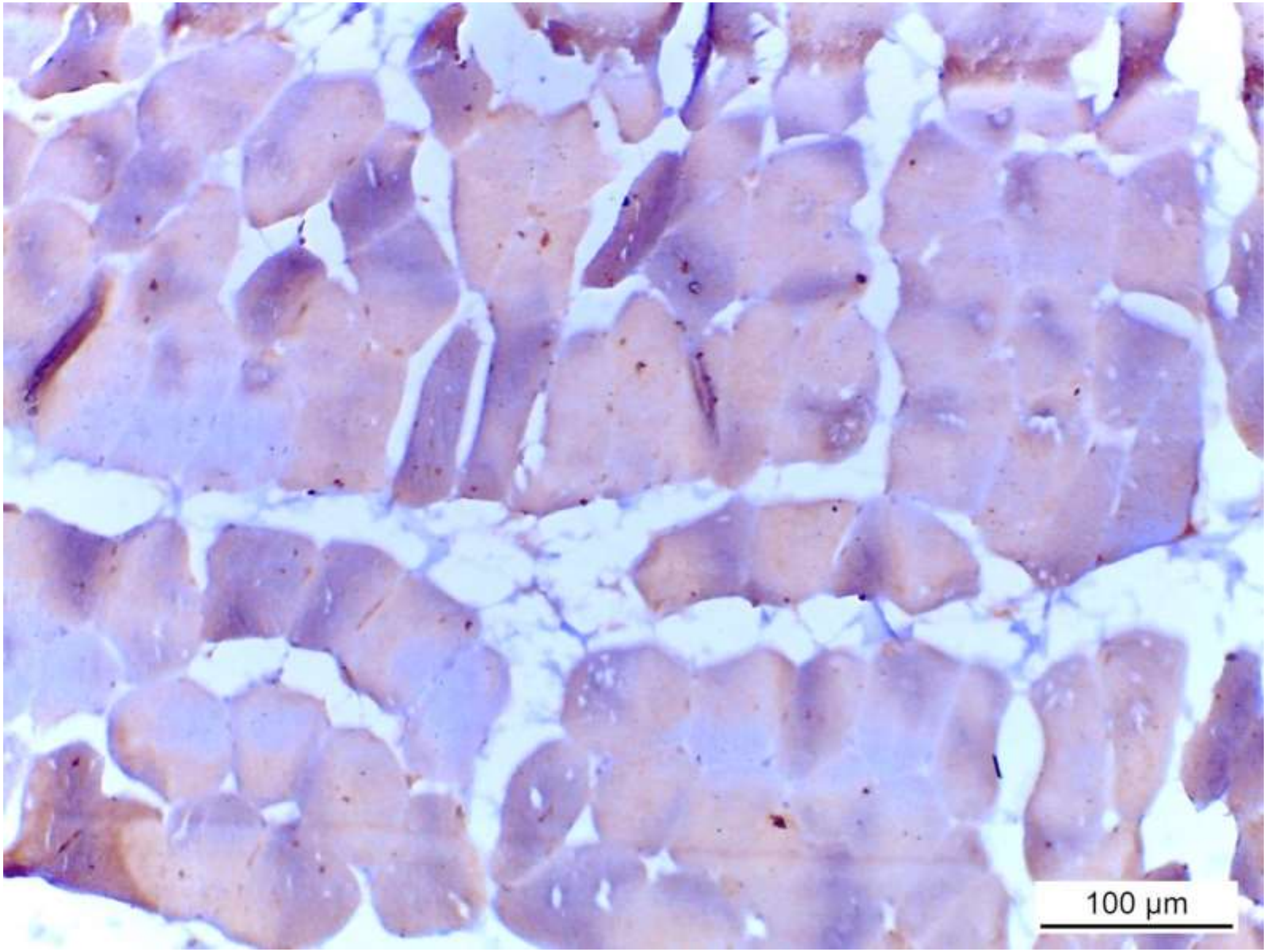
100 µm

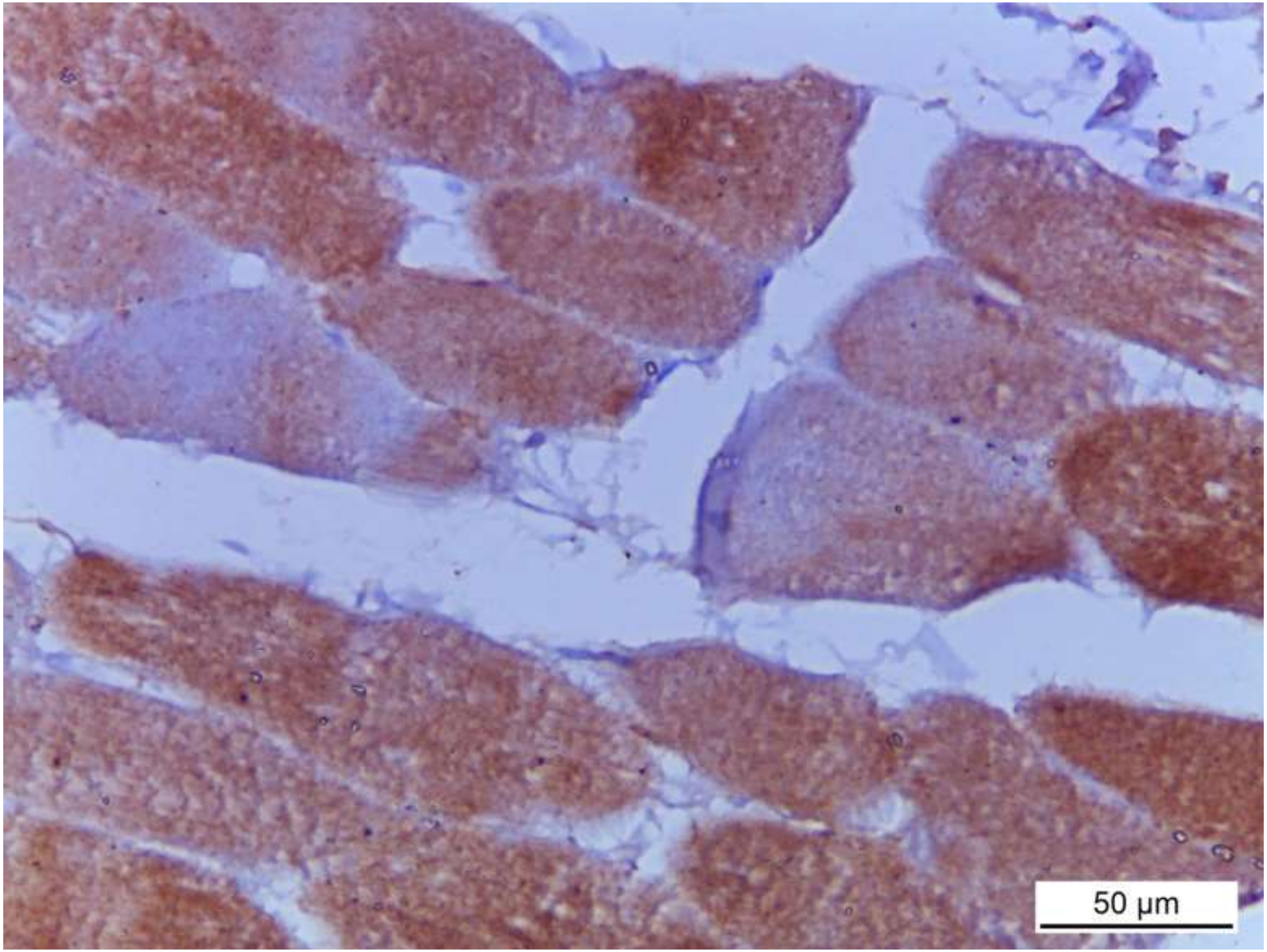
50 µm

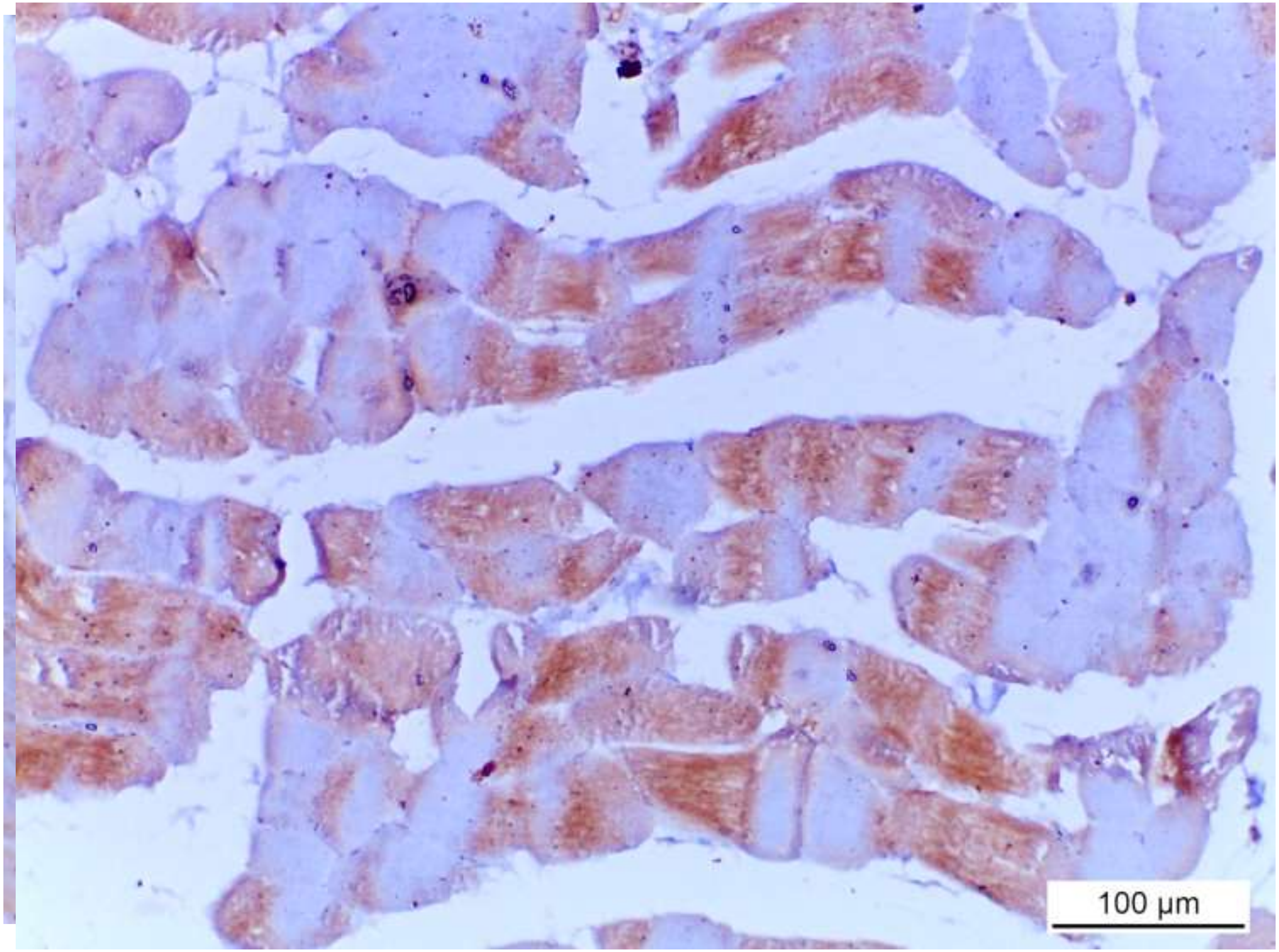
100 µm

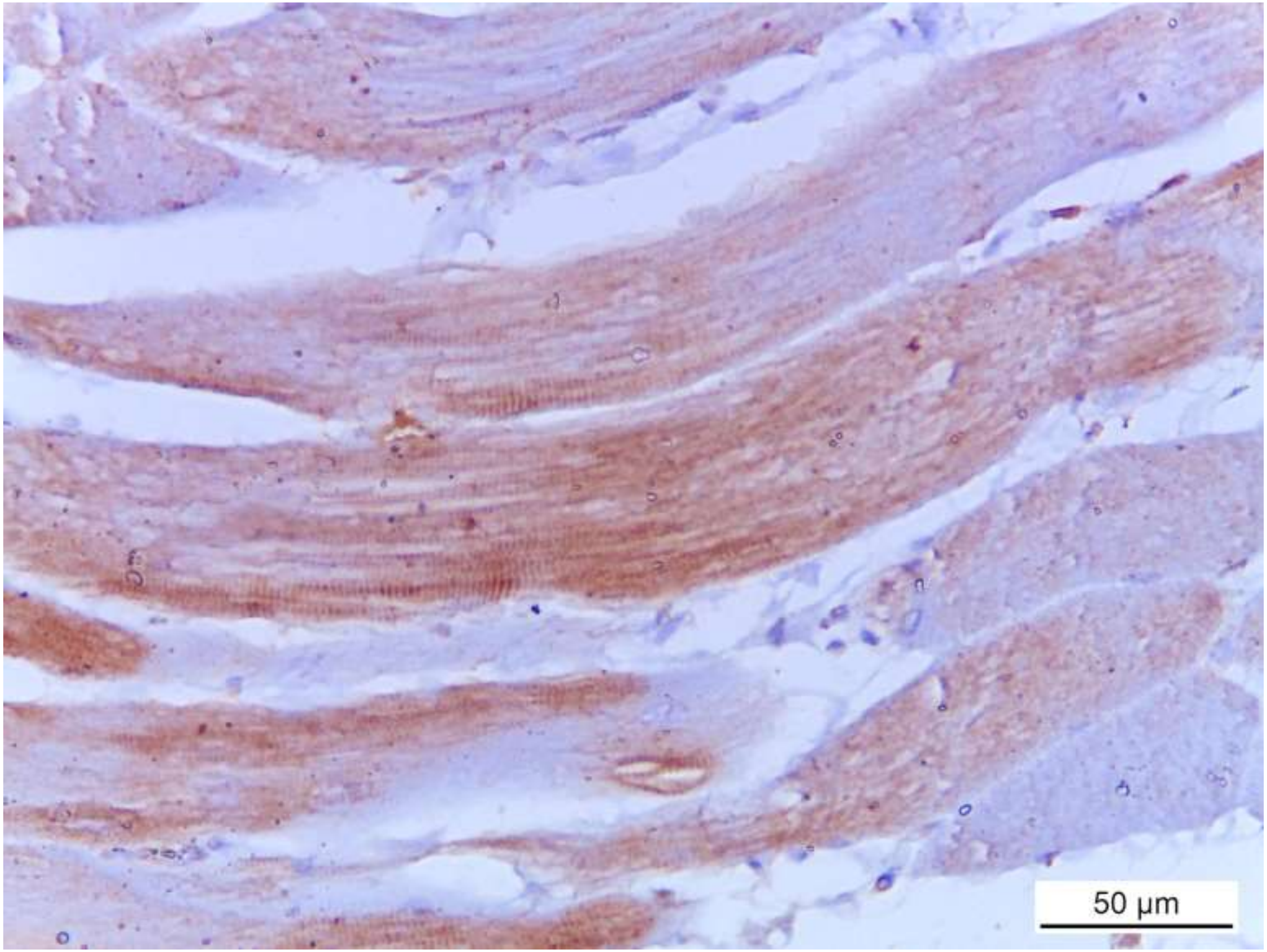
50 µm

Micrograph of transverse and longitudinal sections from gastrocnemius muscle of a diabetic rat (group II) showing a significant increase in NF-kB immunoreactivity in the sarcoplasm of muscle fibers. **Brown color** indicates NF-kB positivity.

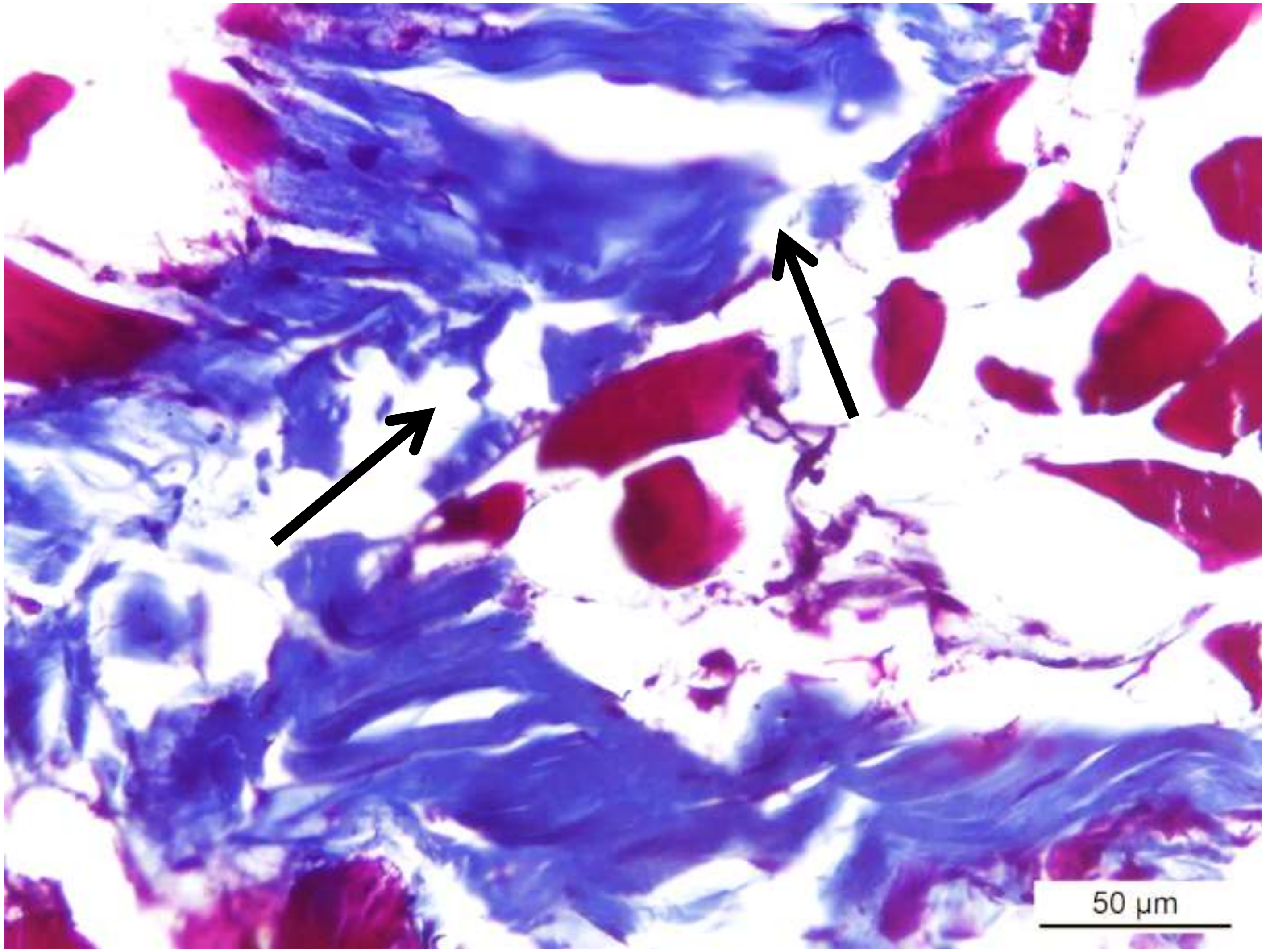
50 µm

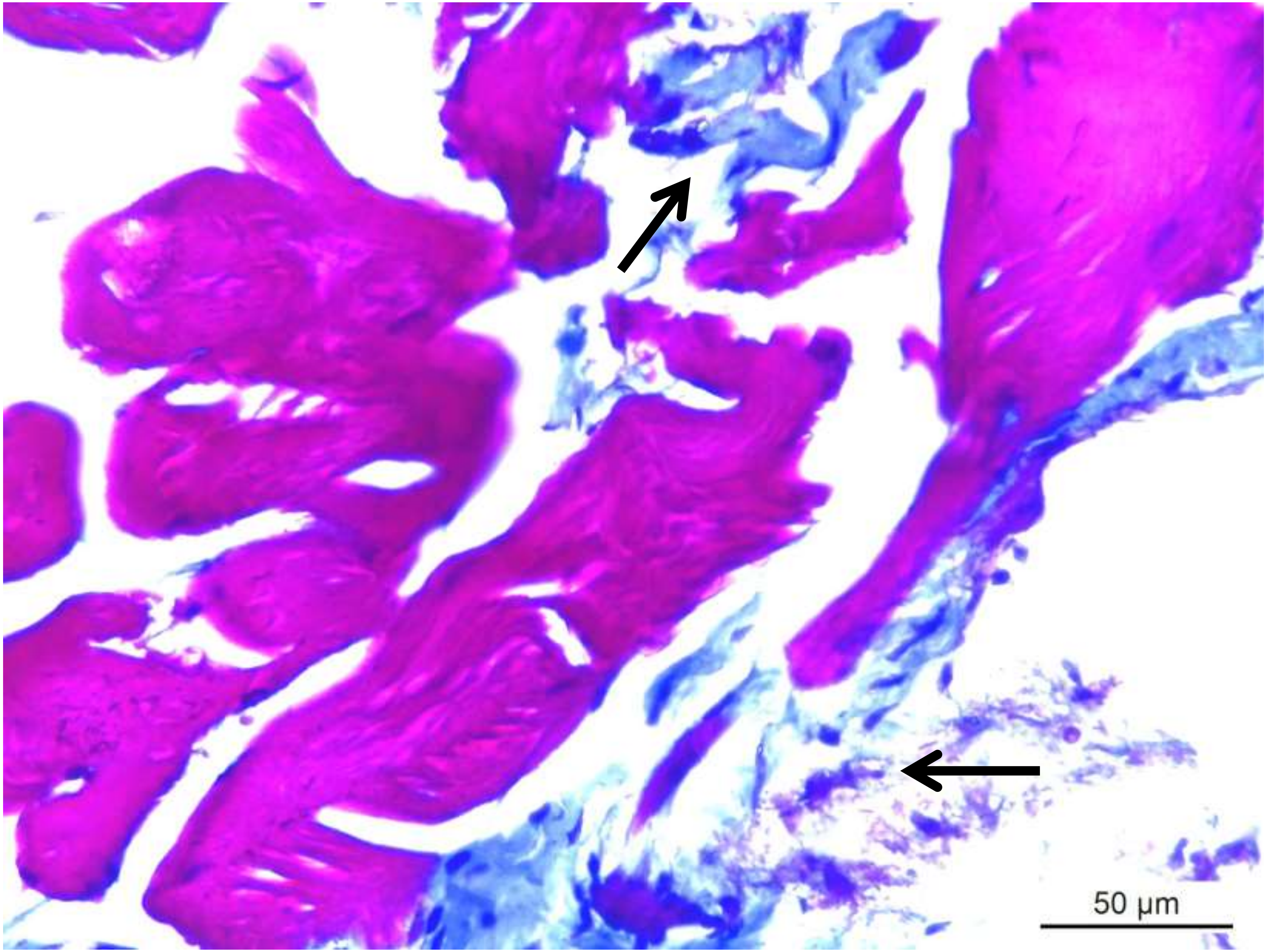
50 µm

Micrograph of transverse section of gastrocnemius muscle of group II (diabetic rats) wide distribution of collagen fibers between the muscle bundles **(arrow).**

Metformin

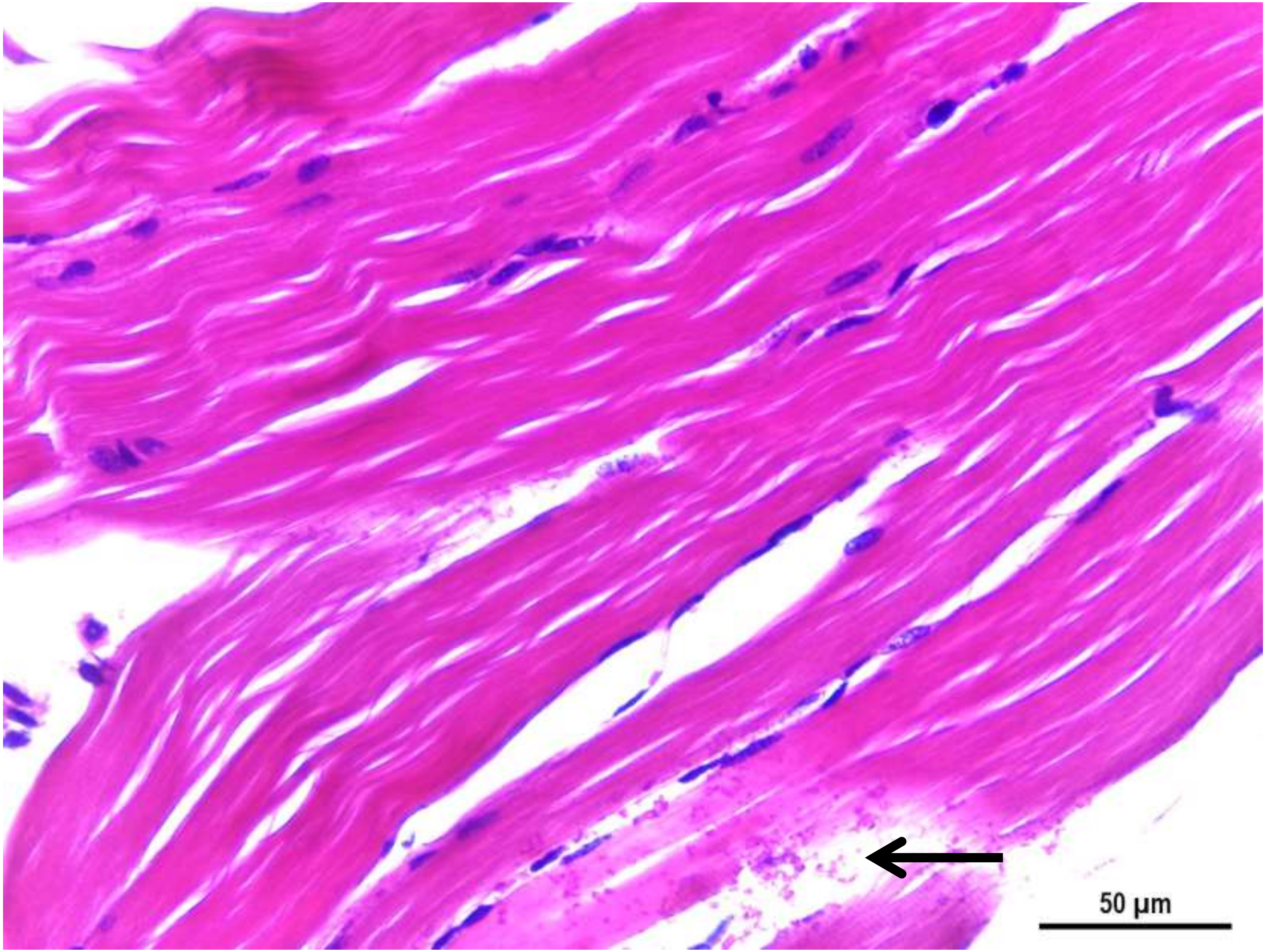
50 µm

Micrograph of longitudinal section from gastrocnemius muscle of a diabetic rat treated with metformin (group III) showing ameliorative effect exhibited histological picture nearly similar to its control group. Splitting of some fibers were also observed **(arrow).**

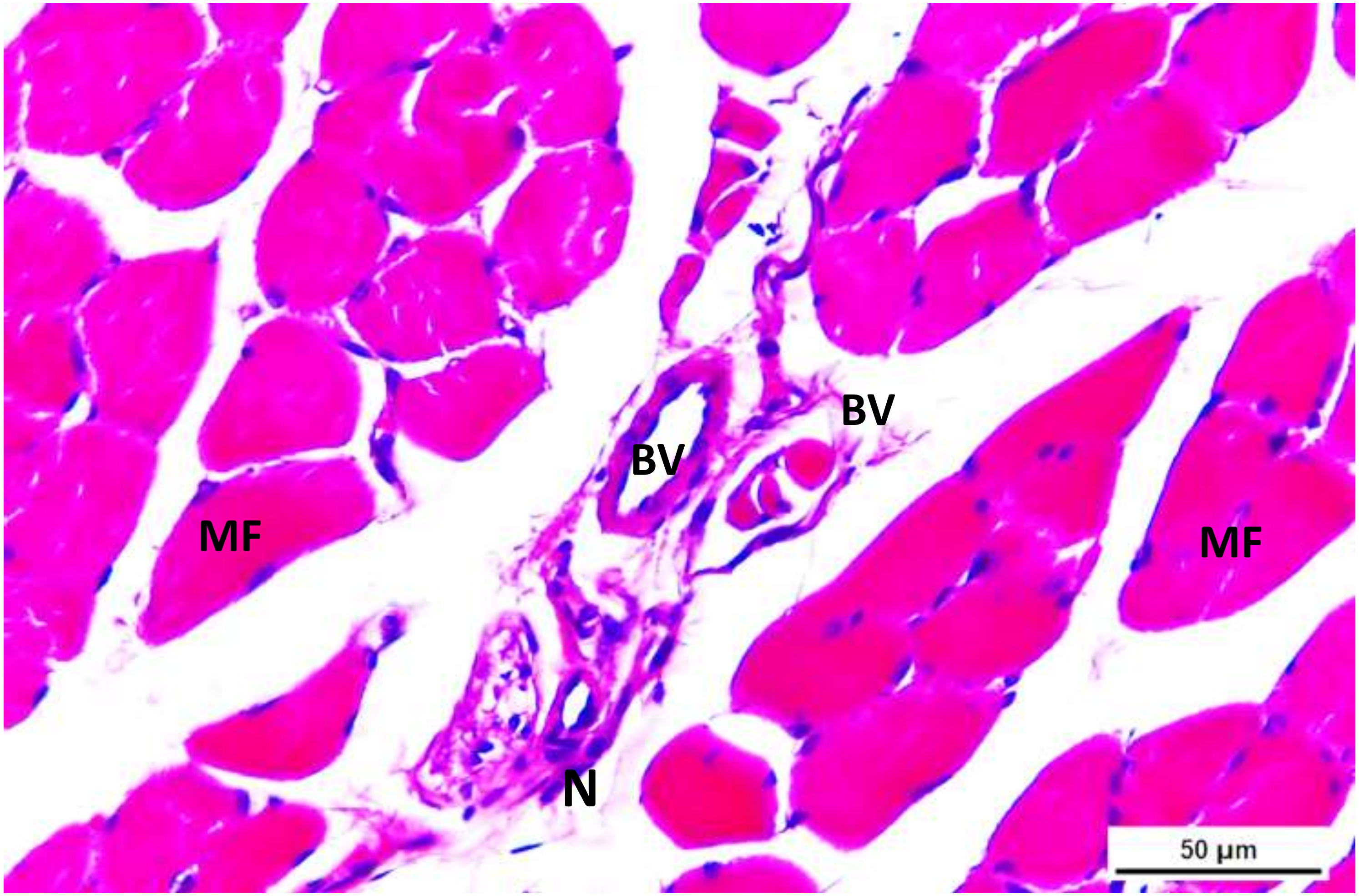
BV
BV
MF
MF
N
50 µm

Micrograph of transverse section from gastrocnemius muscle of a diabetic rat treated with metformin (group III) showing the muscle fibers appeared polyhedral with flattening of adjacent cells and peripheral location of nuclei **(MF).** congested dilated blood vessels were also observed **(BV).** Nuclear crowdies (nuclear clump) also detected **(N).**

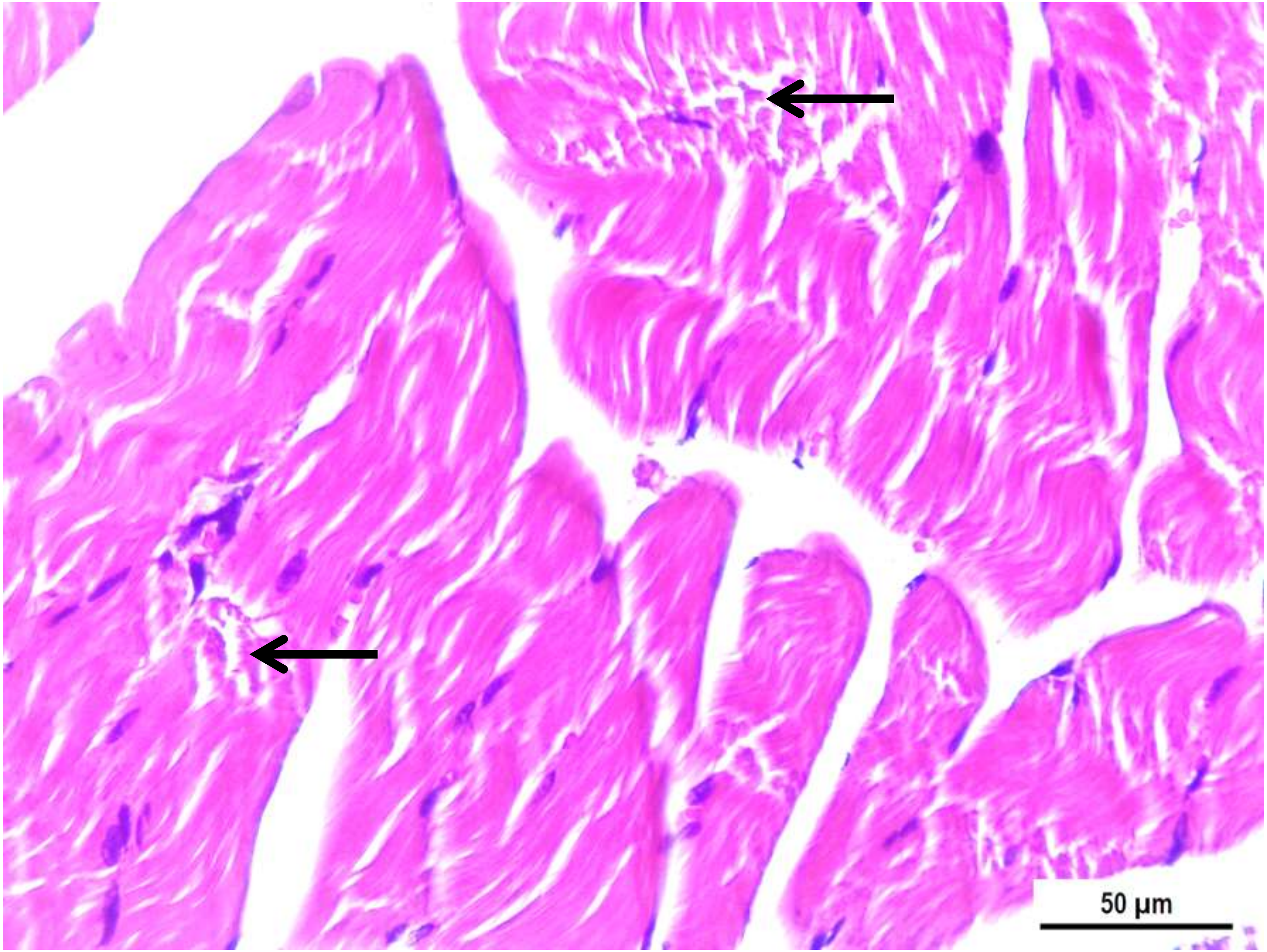
50 µm

Micrograph of longitudinal section from gastrocnemius muscle of a diabetic rat treated with metformin (group III) showing ameliorative effect exhibited histological picture nearly similar to its control group. splitting of the skeletal muscle fibers in association with fibrillolysis were also seen **(black arrow).**

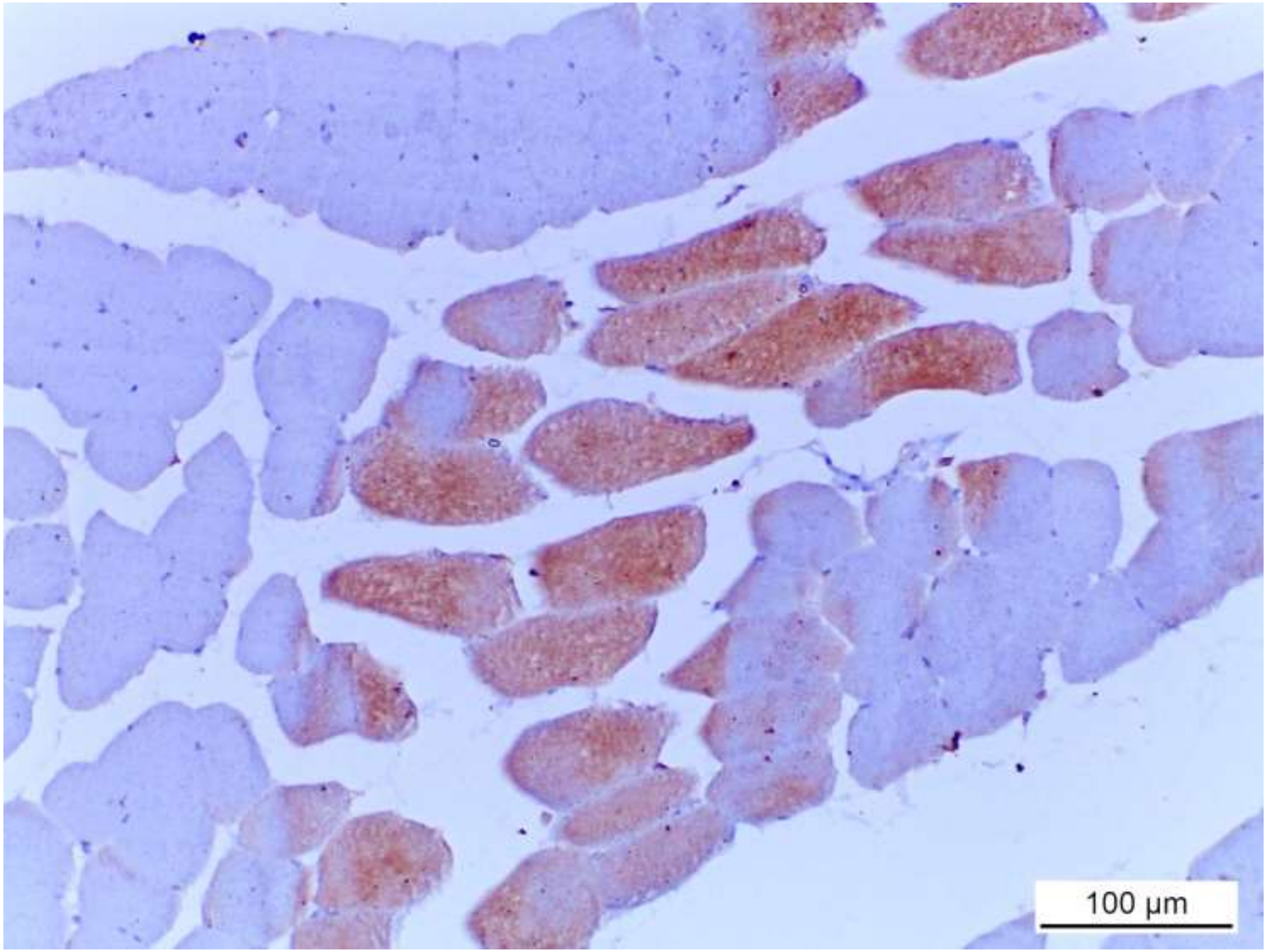
100 µm

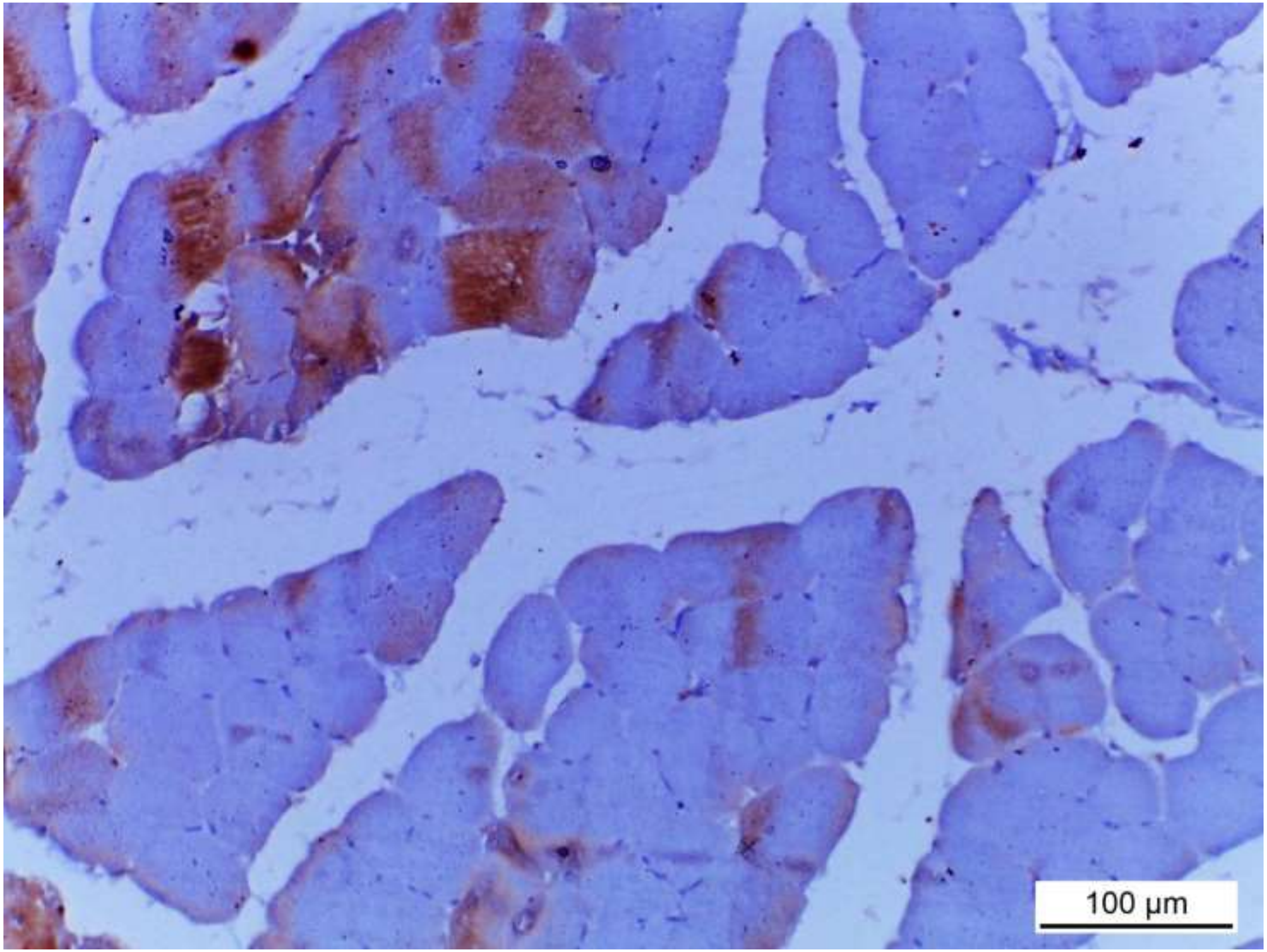
100 µm

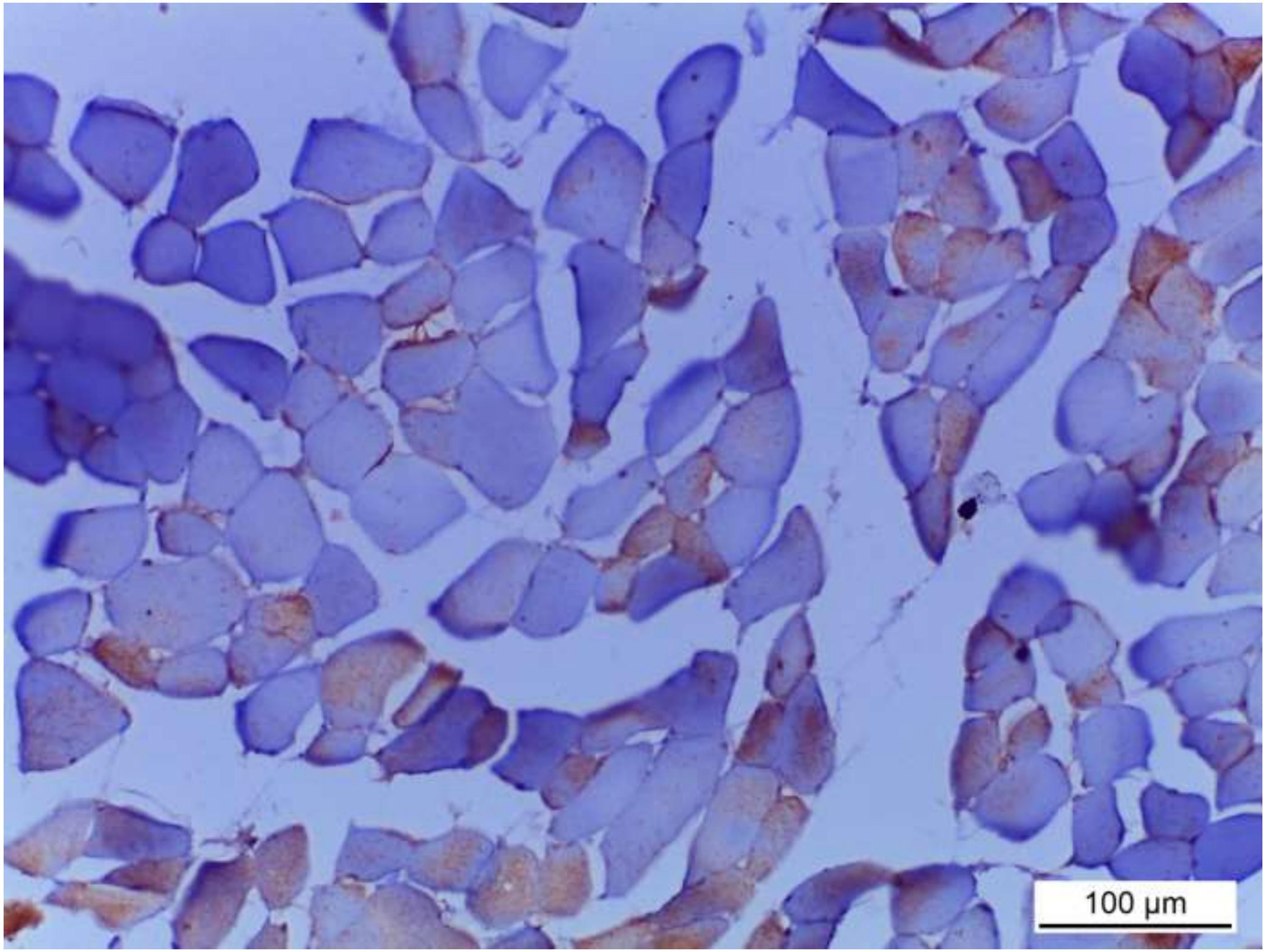
100 µm

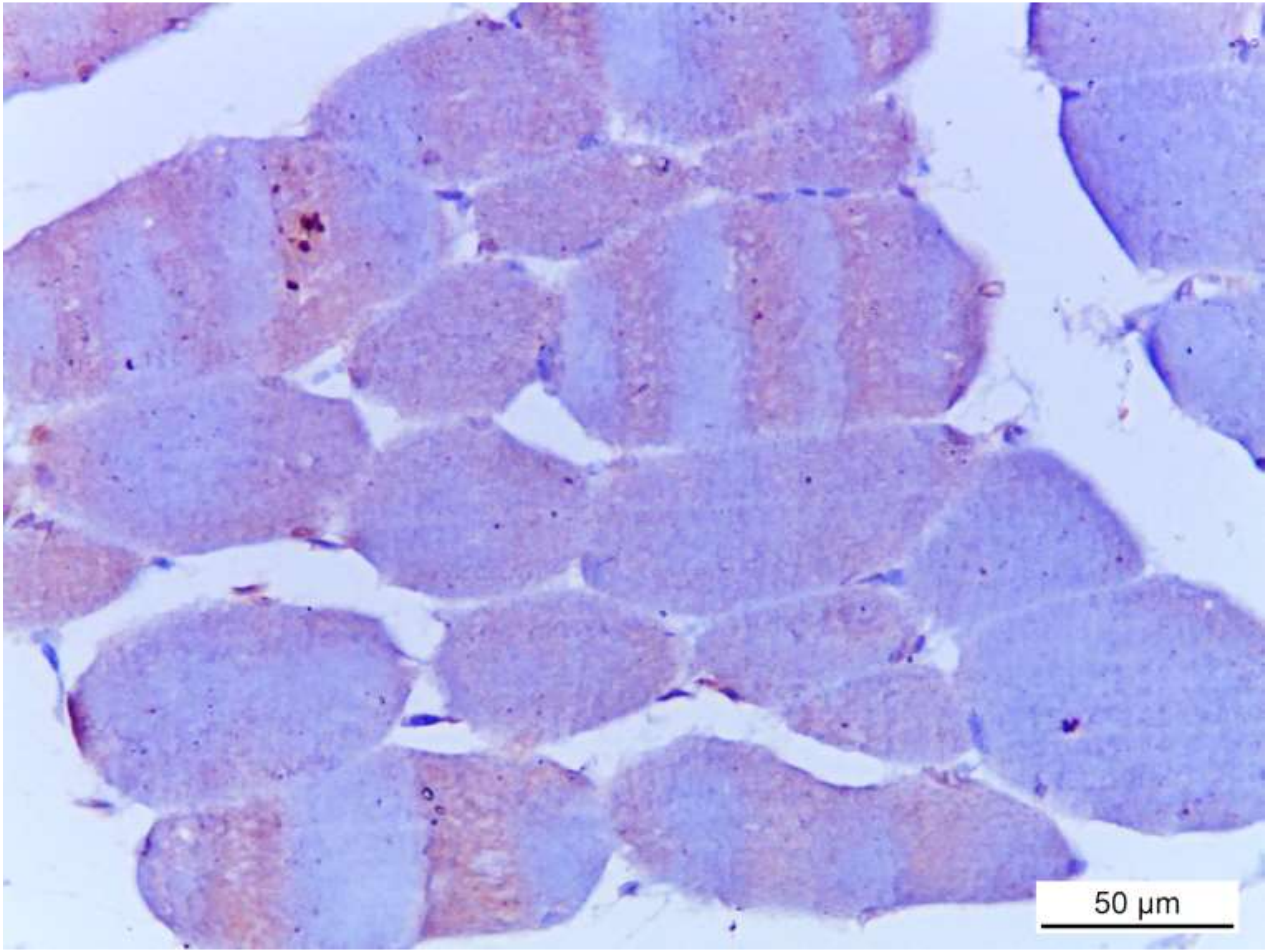
50 µm

Micrograph of transverse section from gastrocnemius muscle of a diabetic rat treated with Metformin (group III) demonstrating a significant reduction in NF-kB immunostaining (moderate posivity) in the sarcoplasm of muscle fibers .

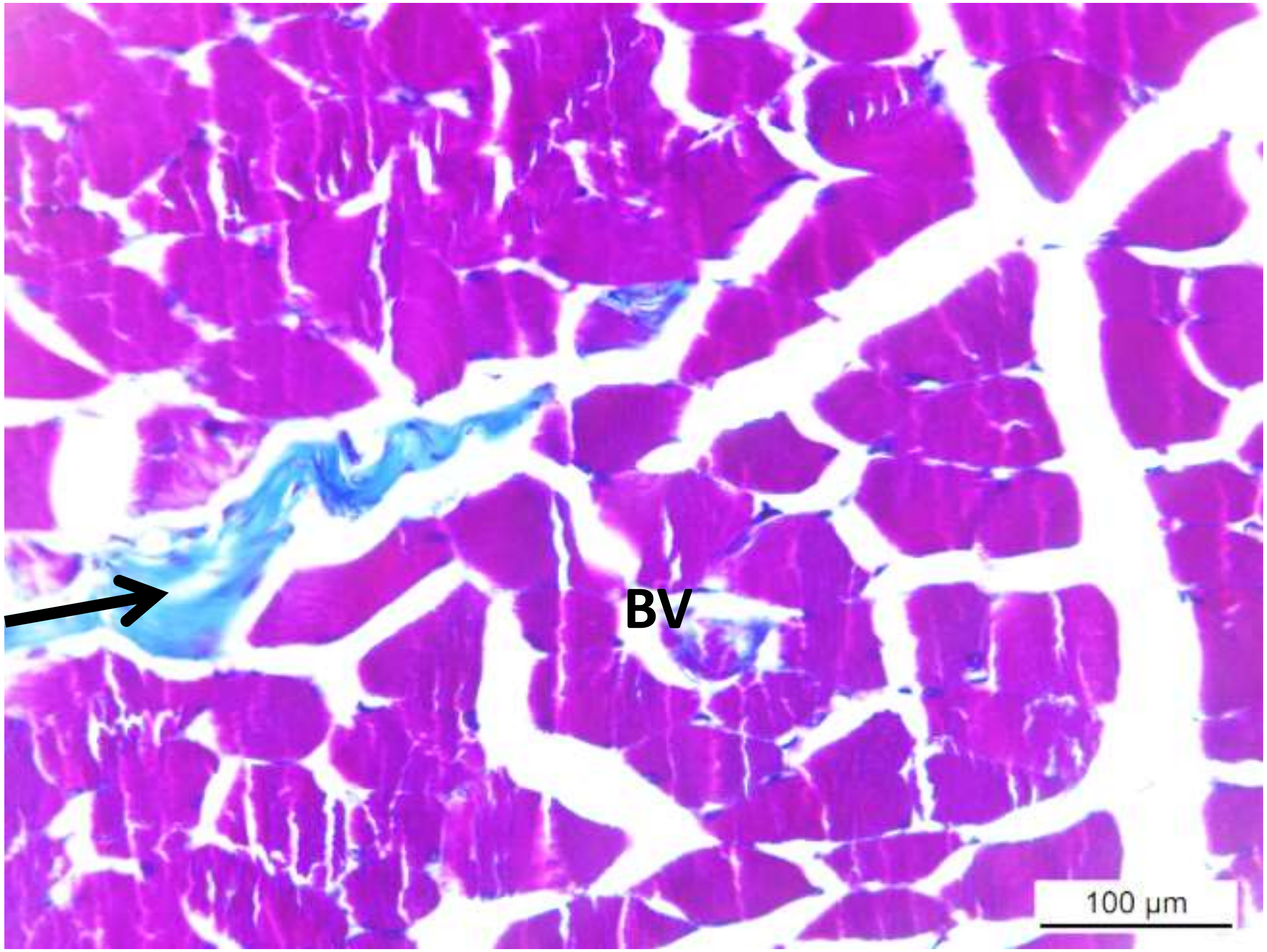
BV
100 µm

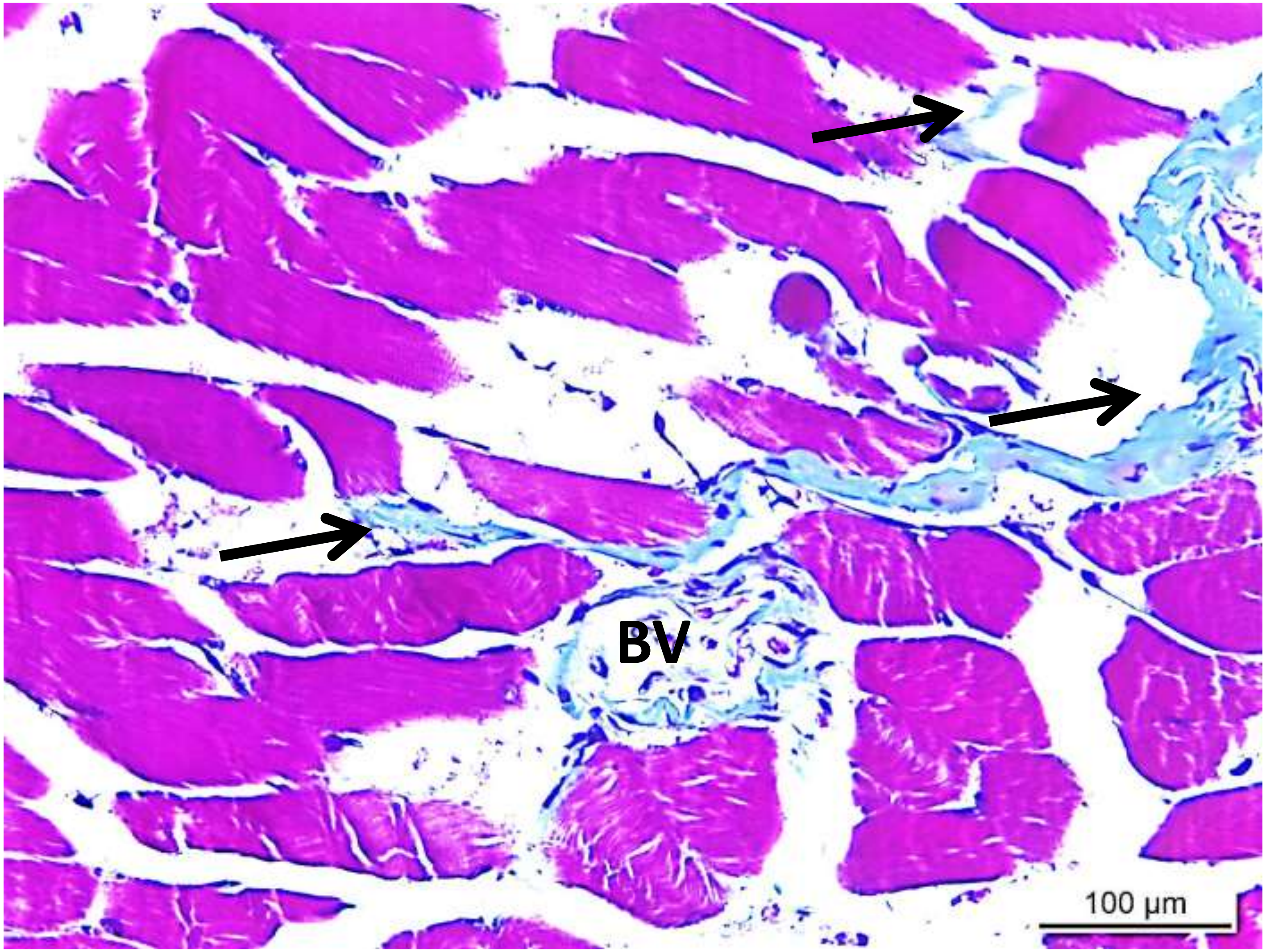
BV
100 µm

Micrograph of transverse section of gastrocnemius muscle of group III (diabetic rats treated with metformin) showing moderate distribution of collagen fibers between the muscle bundles (arrow). around congested blood vessels (BV).

Telmisartan

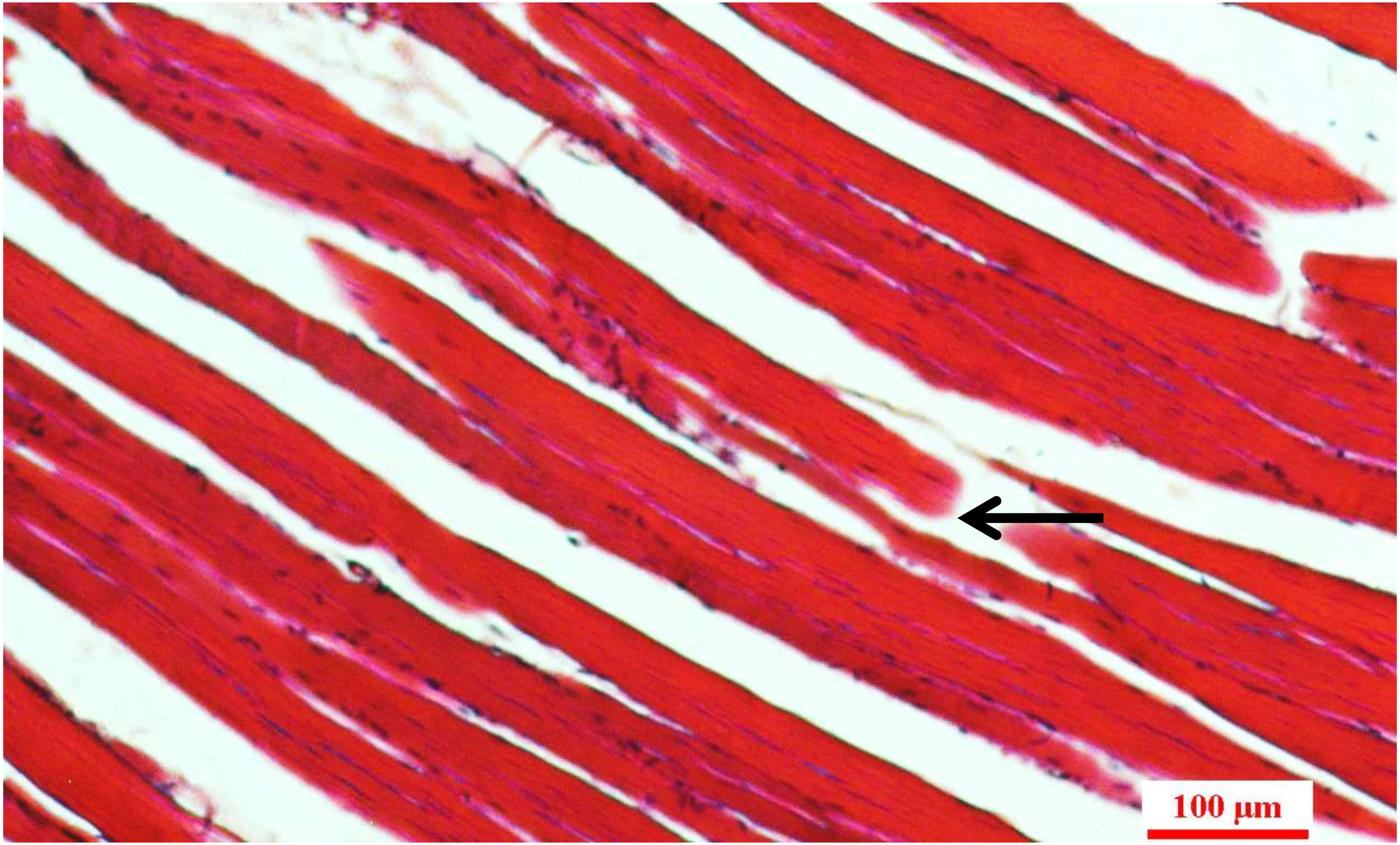
100 µm

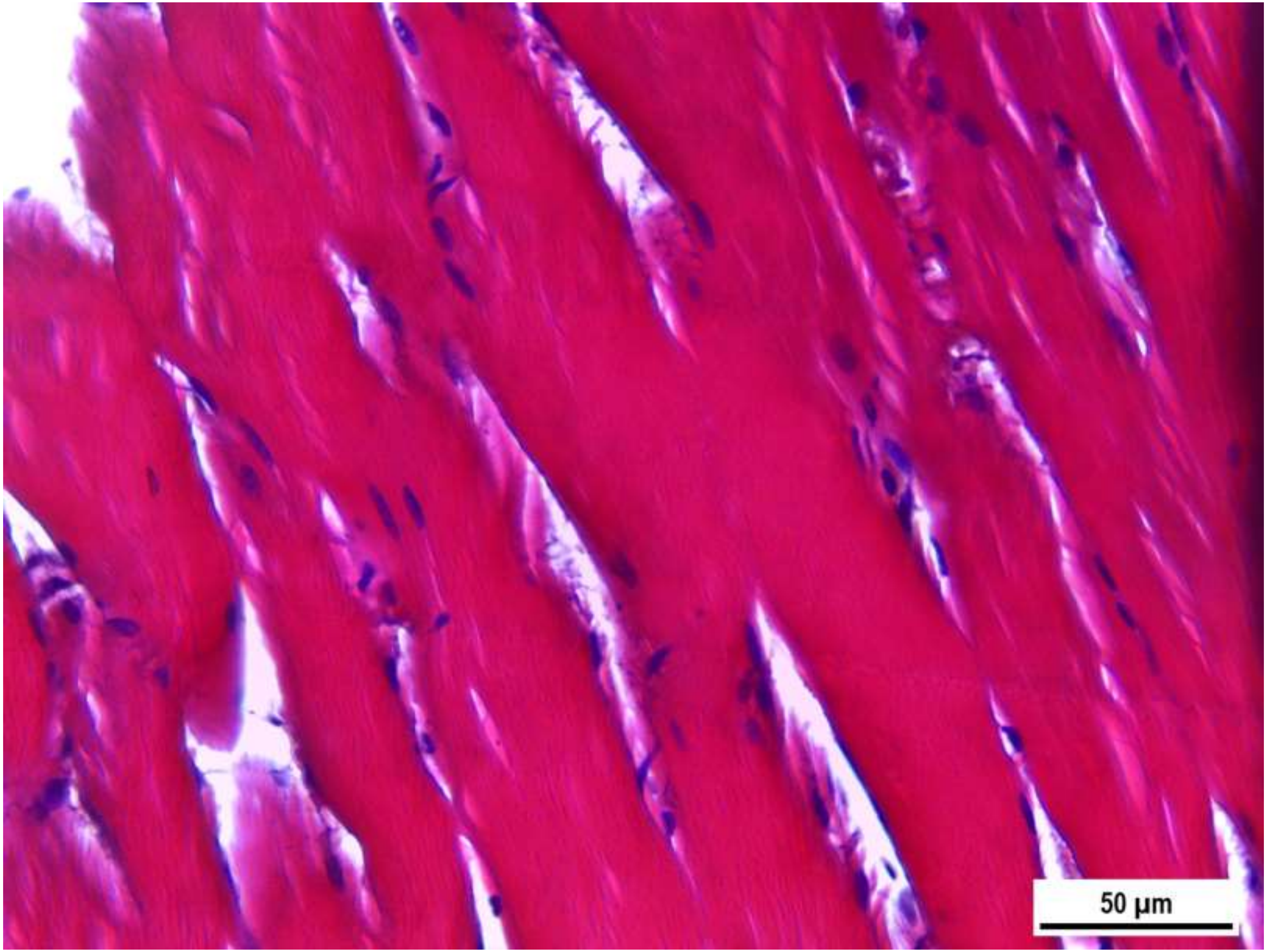
50 µm

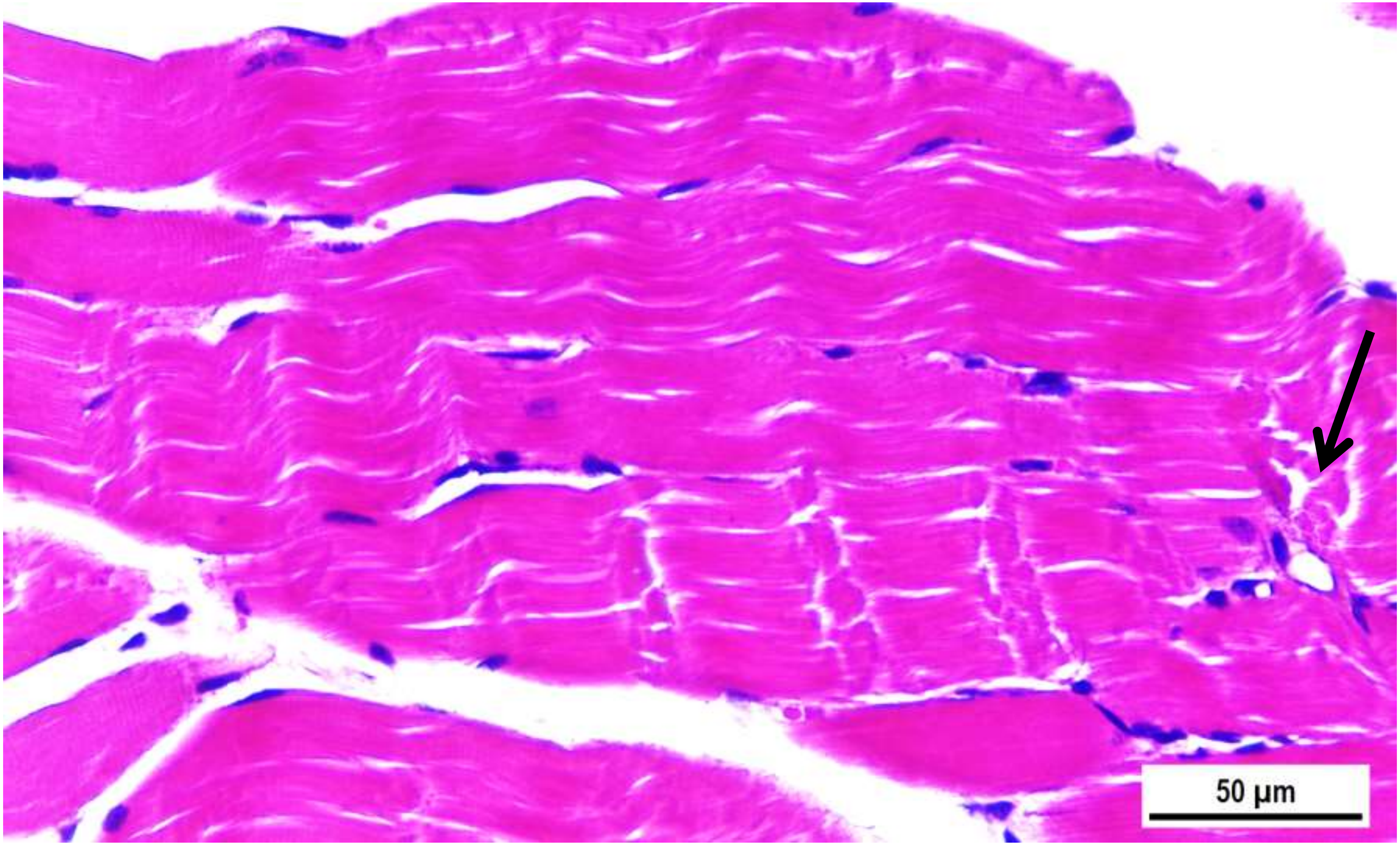
50 µm

Micrograph of longitudinal section from gastrocnemius muscle of a diabetic rat treated with Telmisartan (group IV) showing ameliorative effect exhibited histological picture nearly similar to its control group. elongated cylindrical muscle fibers with acidophilic sarcoplasm and transverse striations Splitting of some fibers were also observed **(arrow).**

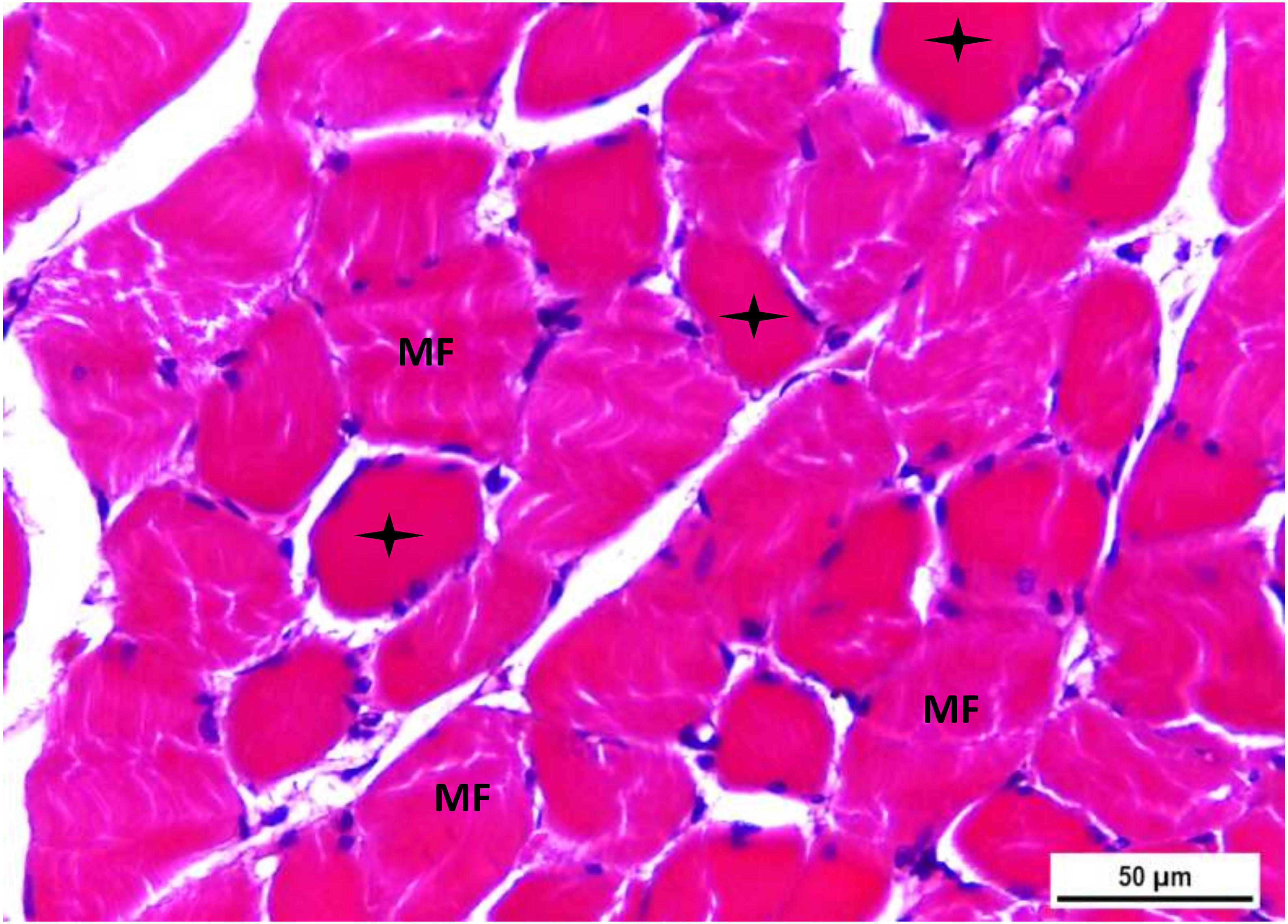

MF
MF
MF
50 µm

Micrograph of transverse section from gastrocnemius muscle of a diabetic rat treated with Telmisartan (group IV) ameliorative effect exhibited histological picture nearly similar to its control group. the muscle fibers appeared polyhedral with flattening of adjacent cells and peripheral location of nuclei **(MF).** Loss of striation in some fibers were also observed **(star).**

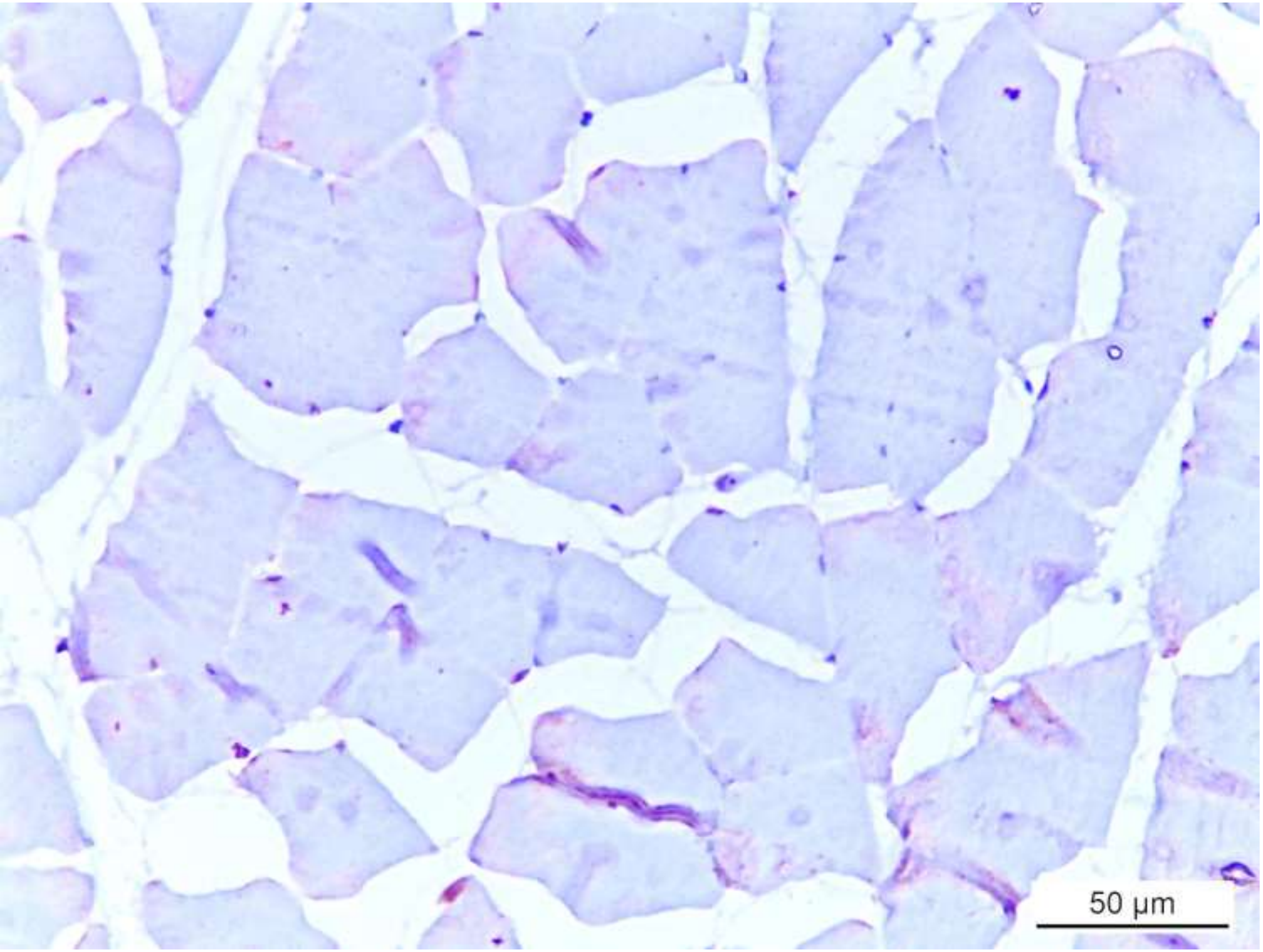
50 µm

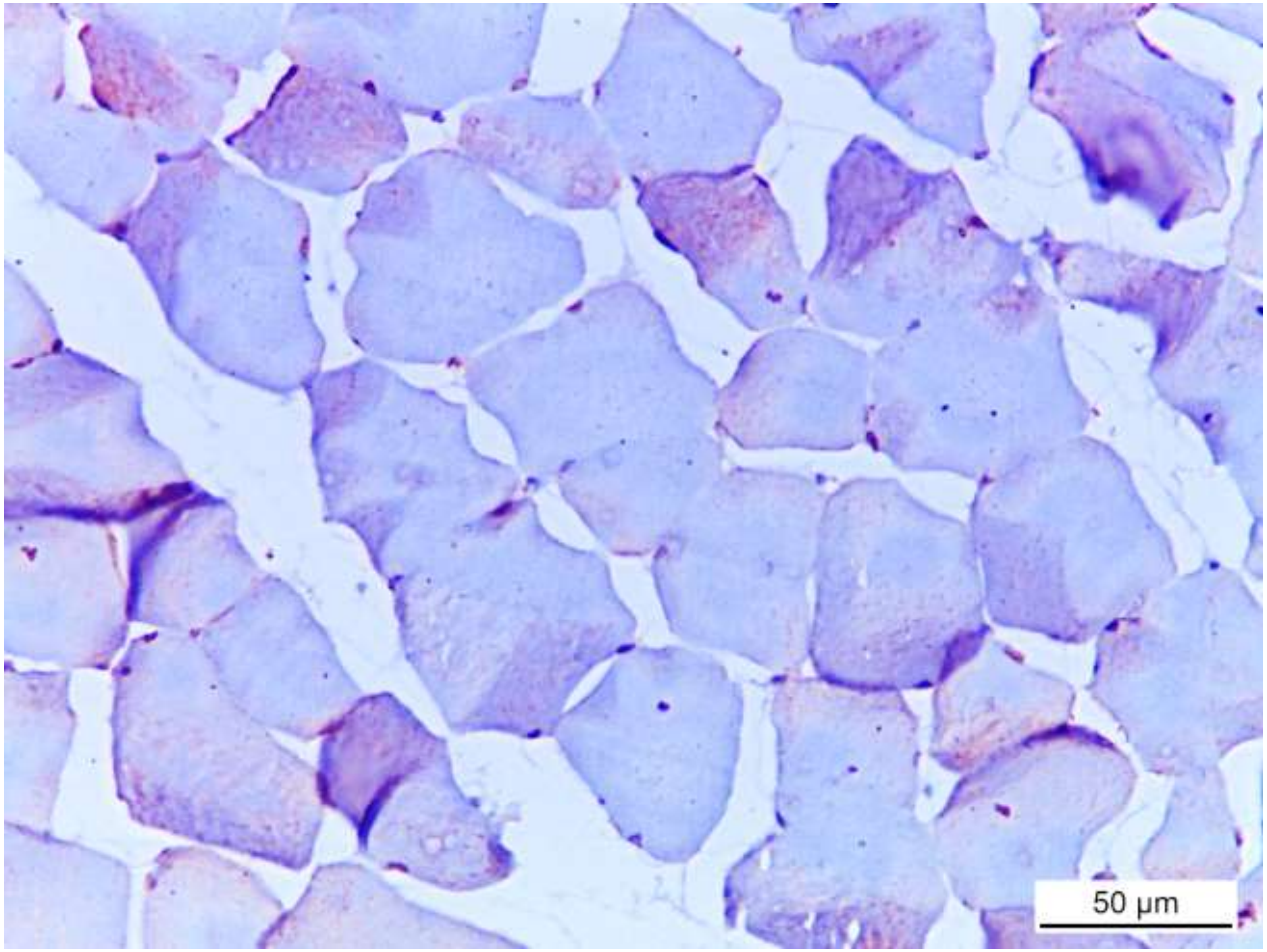
50 µm

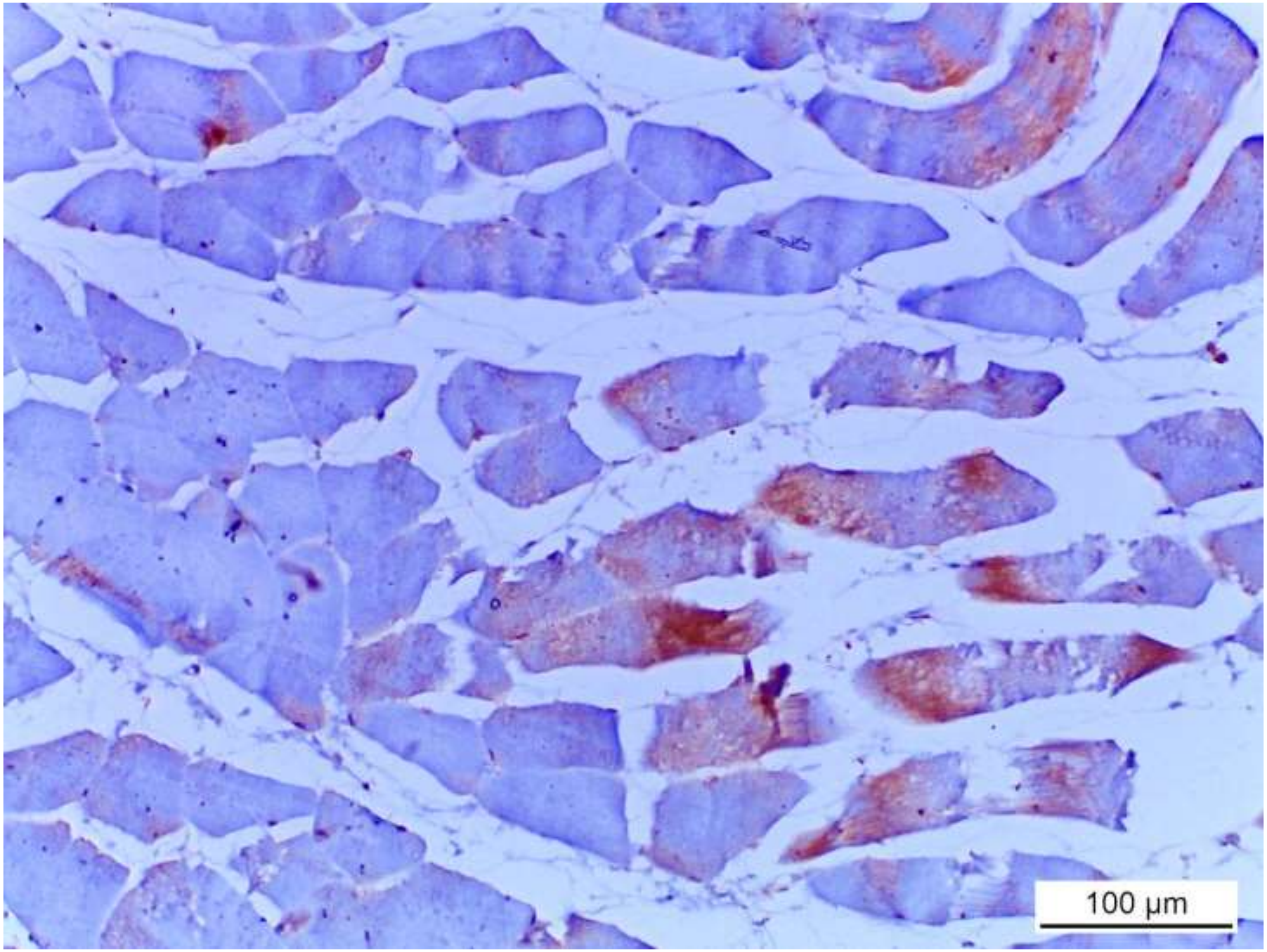
100 µm

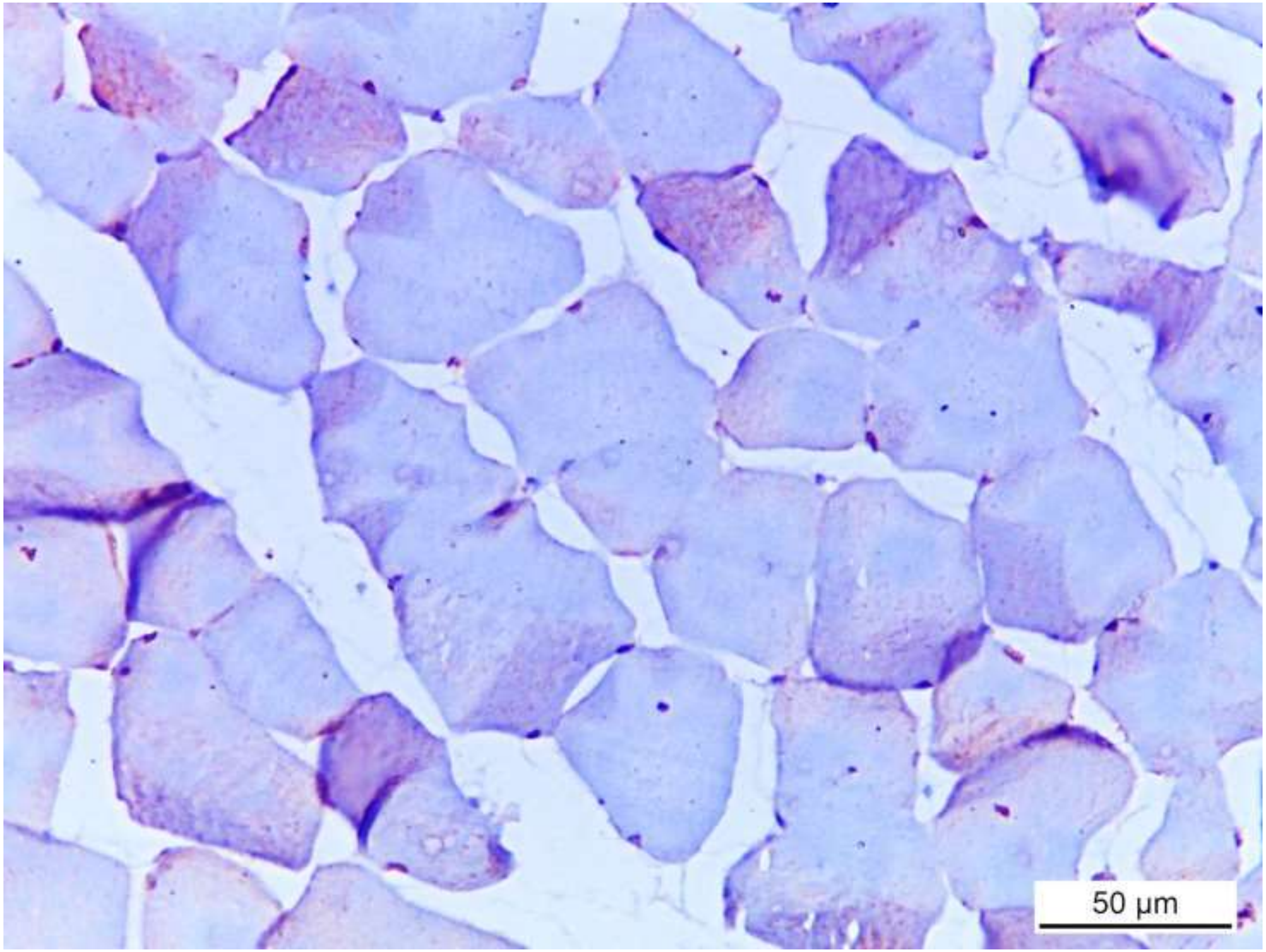
50 µm

Micrograph of transverse section from gastrocnemius muscle of a diabetic rat treated with Telmisartan(group IV) demonstrating no expression of NF-Kb as in the control group except for mild expression in some sections(brown color).

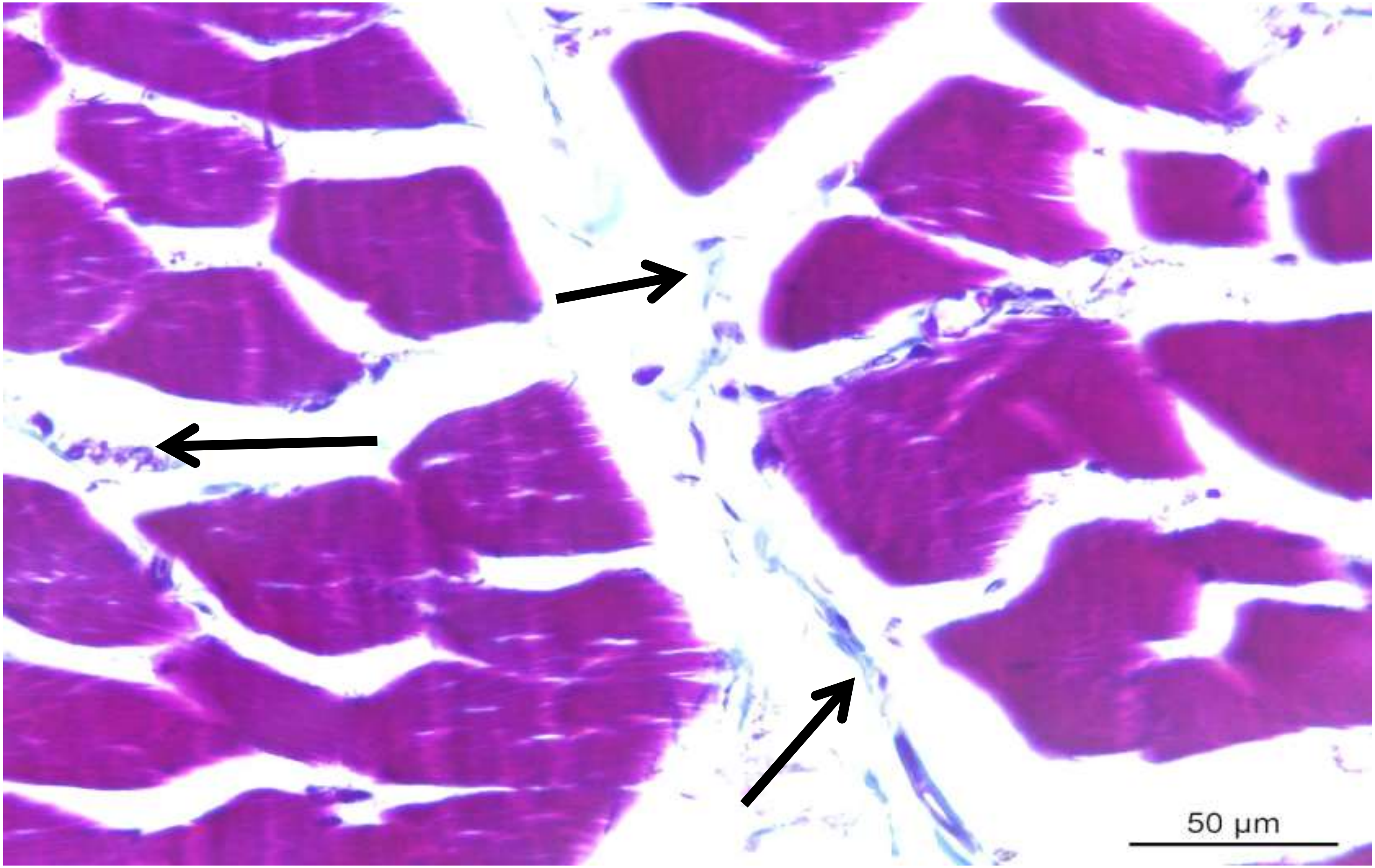
50 µm

Micrograph of transverse section of gastrocnemius muscle of group IV (diabetic rat treated with Telmisartan) showing minimal collagen fibers distribution in between the muscle bundles (arrow).
